# Multi-omics analysis reveals attenuation of cellular stress by empagliflozin in high glucose-treated human cardiomyocytes

**DOI:** 10.1186/s12967-023-04537-1

**Published:** 2023-09-23

**Authors:** Lucia Scisciola, Ugo Chianese, Vicky Caponigro, Manuela Giovanna Basilicata, Emanuela Salviati, Lucia Altucci, Pietro Campiglia, Giuseppe Paolisso, Michelangela Barbieri, Rosaria Benedetti, Eduardo Sommella

**Affiliations:** 1 Department of Advanced Medical and Surgical Sciences, University of Campania “Luigi Vanvitelli”, Naples, Italy; 2 Department of Precision Medicine, University of Campania “Luigi Vanvitelli”, Naples, Italy; 3 Department of Pharmacy, University of Salerno, Fisciano, SA Italy; 4 Biogem, Molecular Biology and Genetics Research Institute, Ariano Irpino, Italy; 5 IEOS CNR, Naples, Italy; 6 Azienda Ospedaliera Universitaria “Luigi Vanvitelli”, Medical Epigenetics Program, Naples, Italy; 7 UniCamillus, International Medical University, Rome, Italy

**Keywords:** Human cardiomyocytes, High glucose, Metabolomics, SGLT2i, Type-2-diabetes mellitus

## Abstract

**Background:**

Sodium–glucose cotransporter 2 (SGLT2) inhibitors constitute the gold standard treatment for type 2 diabetes mellitus (T2DM). Among them, empagliflozin (EMPA) has shown beneficial effects against heart failure. Because cardiovascular diseases (mainly diabetic cardiomyopathy) are the leading cause of death in diabetic patients, the use of EMPA could be, simultaneously, cardioprotective and antidiabetic, reducing the risk of death from cardiovascular causes and decreasing the risk of hospitalization for heart failure in T2DM patients. Interestingly, recent studies have shown that EMPA has positive benefits for people with and without diabetes. This finding broadens the scope of EMPA function beyond glucose regulation alone to include a more intricate metabolic process that is, in part, still unknown. Similarly, this significantly increases the number of people with heart diseases who may be eligible for EMPA treatment.

**Methods:**

This study aimed to clarify the metabolic effect of EMPA on the human myocardial cell model by using orthogonal metabolomics, lipidomics, and proteomics approaches. The untargeted and multivariate analysis mimicked the fasting blood sugar level of T2DM patients (hyperglycemia: HG) and in the average blood sugar range (normal glucose: NG), with and without the addition of EMPA.

**Results:**

Results highlighted that EMPA was able to modulate and partially restore the levels of multiple metabolites associated with cellular stress, which were dysregulated in the HG conditions, such as nicotinamide mononucleotide, glucose-6-phosphate, lactic acid, FA 22:6 as well as nucleotide sugars and purine/pyrimidines. Additionally, EMPA regulated the levels of several lipid sub-classes, in particular dihydroceramide and triacylglycerols, which tend to accumulate in HG conditions resulting in lipotoxicity. Finally, EMPA counteracted the dysregulation of endoplasmic reticulum-derived proteins involved in cellular stress management.

**Conclusions:**

These results could suggest an effect of EMPA on different metabolic routes, tending to rescue cardiomyocyte metabolic status towards a healthy phenotype.

**Graphical Abstract:**

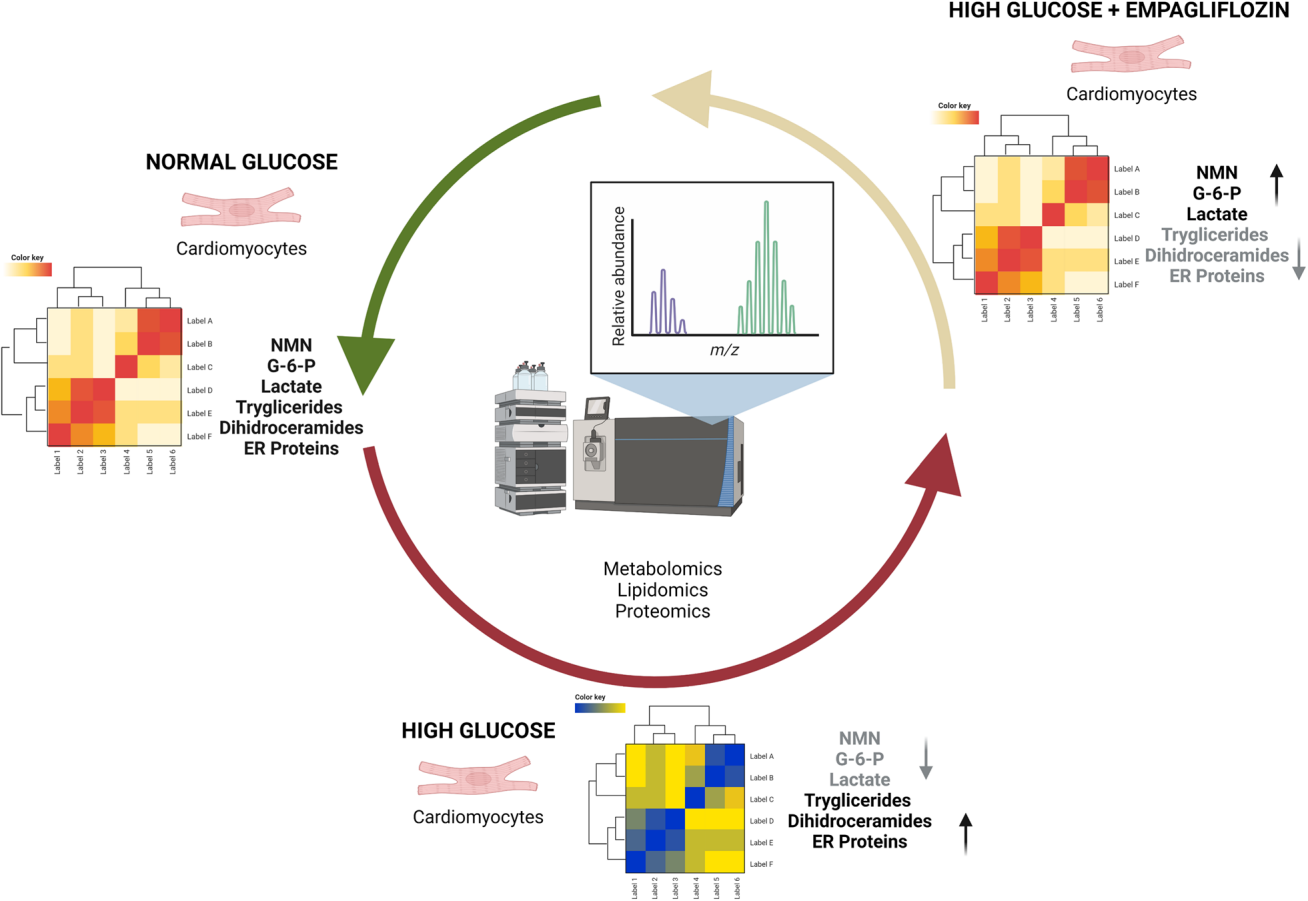

**Supplementary Information:**

The online version contains supplementary material available at 10.1186/s12967-023-04537-1.

## Background

Diabetic cardiomyopathy (DCM) is a common cardiovascular complication in diabetic patients characterized by fibrosis, stiffness, and hypertrophy that induce adverse cardiac structure and performance with consequent diastolic and systolic dysfunction [1]. In severe conditions, DCM can evolve into heart failure, an alteration of cardiac structure/function, inducing an inadequate cardiac output, and an increased left ventricular filling pressure [2, 3]. The DCM pathophysiology has been well-studied, and several mechanisms have been demonstrated to clarify how diabetes promotes cellular dysfunction [1, 4]. Specifically, the high glucose levels enhance the production of advanced glycation end products (AGEs), leading to cardiovascular injury via crosslinking of extracellular matrix molecules [5]. In addition, neglected insulin secretion or insulin resistance promotes a metabolic shift whereby fatty acid intake and β-oxidation are enhanced, trying to provide sufficient ATP production. Nevertheless, β-oxidation is not able to metabolize all incoming fatty acids. Therefore, it results in intracellular lipid accumulation, lipotoxicity, and mitochondrial dysfunction with the production of reactive oxygen species (ROS) and reactive nitrogen species (RNS) [6]. These effects induce cardiomyocyte death, cardiac hypertrophy and inflammation, and a progressive pro-fibrotic response causing extracellular matrix (ECM) remodeling and fibrosis [3, 7]. Over time these changes in cardiac cellular metabolism result in functional cardiac failure that can initially lead to heart failure with preserved ejection fraction and then result in heart failure with reduced ejection fraction Therefore, being able to better identify the mechanisms underlying these metabolic alterations allows us to understand how it is possible to be able to prevent the development of heart failure in the course of diabetic cardiomyopathy. Cardiac metabolic alterations mainly induce pathophysiological changes and have been studied by several multi-omics studies [8–10]. In this regard, multi-omics analyses performed in type 2 diabetes mellitus (T2DM) db/db mice to identify alterations in cardiac function showed that acyl-carnitine, sphingolipid, ceramides, diacylglycerol, and triacylglycerides were more expressed metabolites and associated with impaired mitochondrial function [11]. Moreover, in the myocardium of diabetic rats, metabolome analysis revealed that the level of arachidonic acid was significantly increased, suggesting its pivotal role in the development and progression of DCM [12]. In addition, lipidomic analysis of diabetic hearts demonstrated that lipid metabolites of the cellular membranes, such as cardiolipin lipid class, were altered, suggesting that membrane composition changes influenced cardiac function [13]. Sodium-glucose cotransporter-2 inhibitors (SGLT2i) are, among anti-glycemic drugs, the only class that effectively reduces the risk of cardiovascular death and the occurrence of heart failure in patients with type 2 diabetes [14]. Although the cardio-protective mechanisms of SGLT2i are not yet completely understood, it has been proposed that they could be independent of glycemic status and attributable to their direct action on the failing heart [15–17]. In particular, SGLT2i restores cellular homeostasis by incrementing ATP synthesis by promoting mitochondrial health and alleviating the cytosolic deficiency in ferrous iron [18]. Moreover, SGLT2i inhibits glucose transporter 1, thereby diminishing the accumulation of toxic metabolites and promoting long-chain fatty acid oxidation [19]. Thus, “in vitro” results and “in vivo” evidence suggested that SGLT2i exerted cardio-protective effects changing cardiac metabolism and restoring cardiac metabolic flexibility [20]. Nevertheless, the molecular changes associated with SGLT2i treatment remain unclear, and the detailed picture of cellular response could help unravel metabolic reprogramming following treatment. Specifically, among SGLT2i, Empagliflozin (EMPA) has been demonstrated to be the “first in class drug” for the cardioprotective role in T2DM and No-T2DM patients. More recently, the “EMPA-REG OUTCOME” trial provided evidence for a lower rate of the primary composite cardiovascular outcome and death in T2DM patients without increasing the frequency of hypoglycemic adverse events even when combined with insulin [21, 22]. However, as with other SGLT2i, EMPA has shown beneficial cardiac effects also in no-diabetic hearts [23]. Although the precise mechanism is far from understood, EMPA probably rebalances aberrant nutrient transport and metabolic signatures causative of (diabetic and non-diabetic) cardiomyopathy [24]. Therefore, in the present study, a combined mass-spectrometry-based multi-omics approach was carried out by using as a model human ventricular cardiac myoblasts AC16 exposed to hyperglycemia for 2 days and co-treated with EMPA. Following unsupervised and supervised multivariate analysis, several molecular pathways emerged as key points, revealing a complex metabolic response to EMPA and suggesting a cardiac metabolic reprogramming to restore cellular homeostasis, resulting in a cardioprotective effect.

## Methods

### Cell culture

AC16 human cardiomyocyte cell lines were purchased from EMD Millipore (cod. SCC109). Following the manufacturer’s instructions, the cell line was tested and authenticated for mycoplasma contamination, which was negative. Cells were cultured in Dulbecco’s Modified Eagle's Medium (DMEM)/F12 (cod. AL215A, Microgem) containing 12.5% fetal bovine serum (FBS) (cod. ECS0180L, Euroclone), 1% antibiotics penicillin–streptomycin (cod. ECB3001D, Euroclone), and 1% of l-glutamine (cod. ECB3000D, Euroclone). The cell line was maintained in the incubator at 37 °C and 5% CO_2_. The cells were grown between 4 and 6 passages, and experiments were performed six times. AC16 were exposed to 33 mmol/L D-glucose (cod. G8644, EMD Millipore) for 2 days and treated with EMPA at a concentration of 0.5 µM (cod. S8022, BI 10773, Selleckchem) according to a previous protocol [25, 26]. Normal glucose (NG), considered the control, are cells exposed to normal glucose concentration (5.5 mmol/L) and cultured for 2 days.

### Chemicals

LC–MS-grade Water (H_2_O) acetonitrile (ACN), methanol (CH_3_OH), isopropanol (IPA), 1-butanol (BuOH), methyl tert-butyl ether (MTBE), and additives formic acid (HCOOH), acetic acid (CH_3_COOH), ammonium formate (HCOONH_4_) and ammonium acetate (CH_3_COONH_4_), were all purchased from VWR (Milan, Italy). Deuterated and authentic lipid standards were purchased by Avanti Polar Lipids (Alabaster, AL, USA). Unless stated otherwise, other reagents were all purchased by Merck.

### Metabolome, lipidome, and proteome extraction

Metabolites were extracted: cell pellets were thawed on ice, and 225 µL of ice-cold MeOH containing a mix of deuterated standards were added and vortexed for 10 s. Subsequently, 750 µL of cold MTBE was transferred to the tube, and the solution was continuously agitated in a thermomixer (Eppendorf, Milan, Italy) for 10 min, 300 rpm at 4 °C. Then, 188 µL of H_2_O were added, and samples were put on a vortex for 20 s and centrifuged at 14,680 rpm for 10 min at 4 °C to induce phase separation. The upper MTBE layer and the lower MeOH/H_2_O were separately collected and evaporated using a SpeedVac (Savant, Thermo Scientific, Milan, Italy) and then solubilized before injection in UHPLC-HRMS. As previously reported, the protein pellet was alkylated, reduced and digested with trypsin.

### Metabolome, lipidome, and proteome analysis

Metabolome analyses were performed on a Thermo Ultimate RS 3000 coupled online to a Q-Exactive hybrid quadrupole Orbitrap mass spectrometer (Thermo Fisher Scientific, Bremen, Germany) equipped with a heated electrospray ionization probe (HESI II). The MS was calibrated by Thermo calmix (Pierce™ calibration solutions in both polarities. Lipidome analysis was performed on an Ultimate RS 3000 UHPLC (Thermo Fisher), coupled online to a TimsTOF Pro Quadrupole Time of Flight (Q-TOF) (Bruker Daltonics, Bremen, Germany) equipped with an Apollo II electrospray ionization (ESI) probe. Proteomics analysis used a nanoLC coupled to a tribrid Orbitrap Lumos (Thermo Fisher Scientific, Bremen). Methods were performed as described previously [27–29]. Detailed mass spectrometry conditions and raw data alignment, filtering, and annotation parameters are described in (Additional file 1). For the assessment of repeatability and instrument stability over time, a QC strategy was applied. A quality control (QC) sample was prepared by pooling the same aliquot (10 µL) from each sample. Samples were injected in randomized order, and blank samples were injected regularly and used to assess carryover and exclude background signals.

### Data analysis

The filtered dataset was pre-processed and analyzed using Matlab R2022b (The MathWorks Inc, Natick, MA, USA). Multivariate data analysis was performed using in‐house developed routines and standard Matlab functions. The data analysis was carried out independently on each data set, including low-level (i.e.) data fusion. Data fusion is a chemometric tool that integrates different sources of information (multi-blocks) to extract, simultaneously, the maximum of the information from the various omics approaches and optimize model performance in terms of robustness, consistency, and accuracy. The same biological sample was extracted and analyzed in this experiment using different omics methods. The original pre-processed data (low level) with the same sampling mode were concatenated row by row.

### Data pre-processing

The data were pre-processed independently for the three modalities in the first step. While the total ion sum in Matlab normalized Lipidomics and Metabolomics datasets. Proteomics data sets were normalized by the total sum of abundance values over all the peptides identified in a sample. Then, the three datasets were treated with the same approach. The missing values and zeros were replaced with one-fifth of the minimum value recorded in the data set for that molecule. Logarithm values were then calculated using a base of 10. Before other chemometric modeling, the data were scaled by autoscaling, in which each variable was first centered by removing its average from the data and then scaled by dividing by its standard deviation. Ultimately, each pre-processed block was normalized before multi-block approaches, removing the scale or rank differences, using Frobenius’ norm.

### Exploratory tools: principal component analysis (PCA) and SUM-PCA

After column autoscaling, principal component analysis (PCA) was performed on the data sets. PCA is an unsupervised statistical technique that enables data reduction and visualization, representing the variance of the data matrix on different orthogonal variables (“Principal components” or PCs) that contain the maximum variance. The PCs define a new space to represent the samples (‘‘scores’’) with underlying similarities and dissimilarities. The observed differences can be interpreted by looking at the "loadings" plots, i.e., the cosine of the angle between PC and the original variables. To visualize the effectiveness of preliminary discrimination, the Hotelling's (T^2^) confidence ellipses for each class were added to the score plots. The T^2^ confidence ellipses were calculated independently for each class, with the confidence level set at 95%. SUM-PCA is the simplest multiblock analysis that applies PCA to the fused data block. Each block is modeled with identical super scores (T_sup_) but specific block loadings (P_b_) and residuals (E_b_). T_sup_ represents the best summary of all measured block characteristics and can be considered the consensus score. The regression of the consensus scores on the combined block score matrix expresses the contribution of each block to the consensus (block weight matrix (W))**.** W provides information about each block’s contribution to consensus for each principal component (T_sup_ = TW).

### Chemometric classification models: partial least squares-discriminant analysis (PLS-DA)

Partial Least Squares-Discriminant Analysis (PLS-DA*)* is a supervised classification algorithm widely used on mass spectrometry data sets. The core of the algorithm PLS-DA is based on PLS regression applied to a binary coded categorical variable Y for classification purposes. Given the matrix X whose rows contain the independent spectra, PLS-DA defines the best relationship between X and the categorical dummy response. The categorical information can be expressed as a binary matrix Y (coded as 0 and 1) describing class membership. Indeed, for the 4 classes, each spectrum belonging to the first class is coded as [1 0 0 0], the second class as [0 1 0 0], and so on. Finally, the PLS kernel is used to compute a regression model that relates the predictor matrix and Y. Classification is then based on linear discriminant analysis (LDA) applied to the predicted Y or the PLS values. PLS-DA has performed independently on each modality. Four replicates selected randomly (the same for each class) were included in the training set, and the remaining two were in the test set. The optimal number of latent variables (LV) was chosen to minimize the misclassification error and maximize the accuracy during the cross-validation (leave one out per class). Each model was evaluated according to the confusion matrices, which provide graphical details about the type of errors. For each class, two parameters are used to identify the correctness of the class predictions: True Positive (TP), i.e., the number of correctly classified samples, and True Negative (TN), the sum of samples wrongly classified for that specific class. On the other hand, the terms False Positive (FP) (the sum of the other class members classified in our class) and False Negative (FN) (the sum of the samples not part or not classified in our class) refer to incorrect predictions. Then, for each class, *Sensitivity*, *Specificity,* and A*ccuracy* were calculated. Finally, the spectral variables that contributed most to discrimination were checked against the indices of the importance of the variables in the projection (VIP). VIPs scores were compared with the p-values obtained by N-way analysis of variance (ANOVA).

## Results

### Multi-omics data analysis

**Fig. 1:**
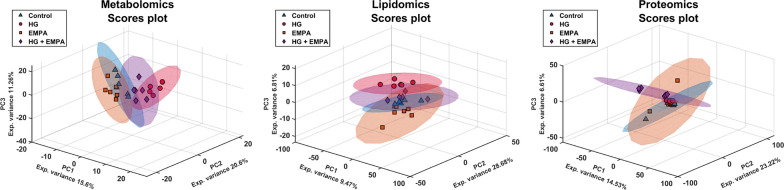
PCA scores plots of Metabolomics, Lipidomics, and Proteomics. PCA scores plots have a confidence ellipsis (95%) for each class. The datasets were pre-processed independently for the three modalities: (1) normalized (2) the missing values and zeros were replaced with one-fifth of the minimum value recorded in the data set for that molecule, (3) logarithm values of the base of 10, and (4) autoscaling

**Fig. 2:**
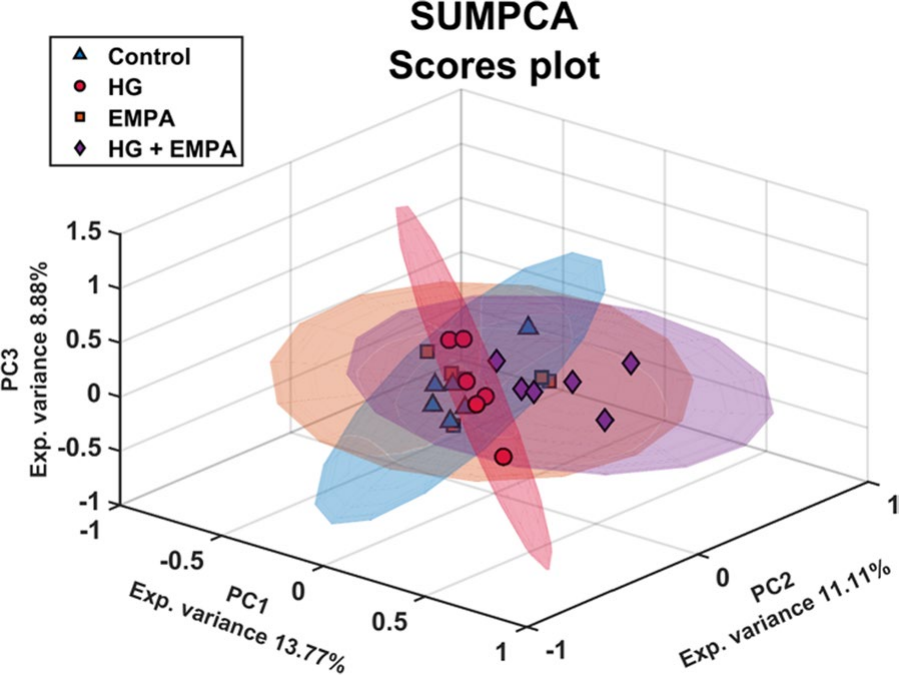
SUMPCA of the three omics datasets

Following mass spectrometry analysis, a dataset containing 810 annotated metabolites (MSI level = 2) and 1700 proteins (FDR < 0.01) was used for multivariate analysis (Additional file 1: Tables S1, S2, S3). Given the importance of simultaneously comparing the four cellular conditions, we started with PCA as the first exploratory tool to reveal patterns in the data and relationships between groups. PCA was independently performed on the pre-processed and auto-scaled data set for all modalities. The scores plot for each omic are reported in Fig. 1. A clear difference between the cells treated with high glucose (HG) against control and EMPA-treated cells is evident from metabolomics and lipidomics data. On the other hand, proteomics showed a higher biological variation has a more significant impact; thus, the trend observed in metabolomics and lipidomics was highly affected by this factor. In both scenarios, cells treated with HG and EMPA show behavior intermediate between the two groups. To integrate the information obtained by the three omics techniques, a SUMPCA was performed for the three datasets. Figure 2 underlines that control and EMPA-treated cells have similar behavior, while a greater separation can be observed compared to HG-treated cells.

### Extraction of relevant biological features

Subsequently, a PLS-DA model was performed independently for each data set to identify biologically essential features for treatment differences.

**Fig. 3:**
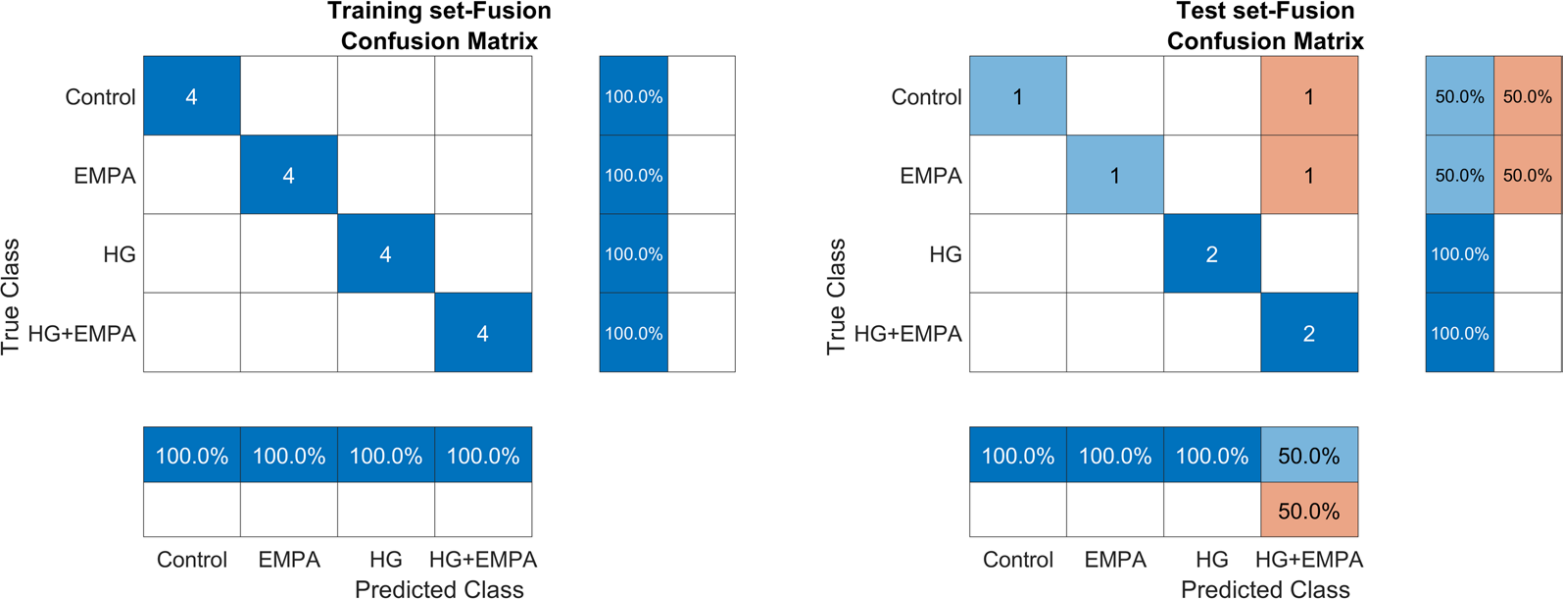
Graphical representation of confusion matrices obtained from PLS-DA model of the fused modality. The reported disorder matrices refer to PLS-DA performance in training and test phases

Looking at the performances (Additional file 1: Tables S4, S6) and the confusion matrices (Fig. 3) obtained for the test set, it can be concluded that cells treated with HG were never misclassified, indicating that they have specific molecular changes that are not common with control and EMPA-treated cells.

In contrast, the remaining three classes (control, EMPA, and HG + EMPA) were misclassified. This indicates high biological similarity among them, likely attributed to the effect of EMPA, which tends to restore the cellular metabolic status altered by HG to a metabolic state closer to control and EMPA alone treated cells.

From the analysis of the VIP scores obtained from PLS-DA, it was possible to extrapolate the most important molecules for classification, and the top 20 compounds for each omic were reported in Additional file 1: Figure S1, S2, S3.

### Metabolome alterations in HG-treated AC-16 cells and EMPA effect

**Fig. 4:**
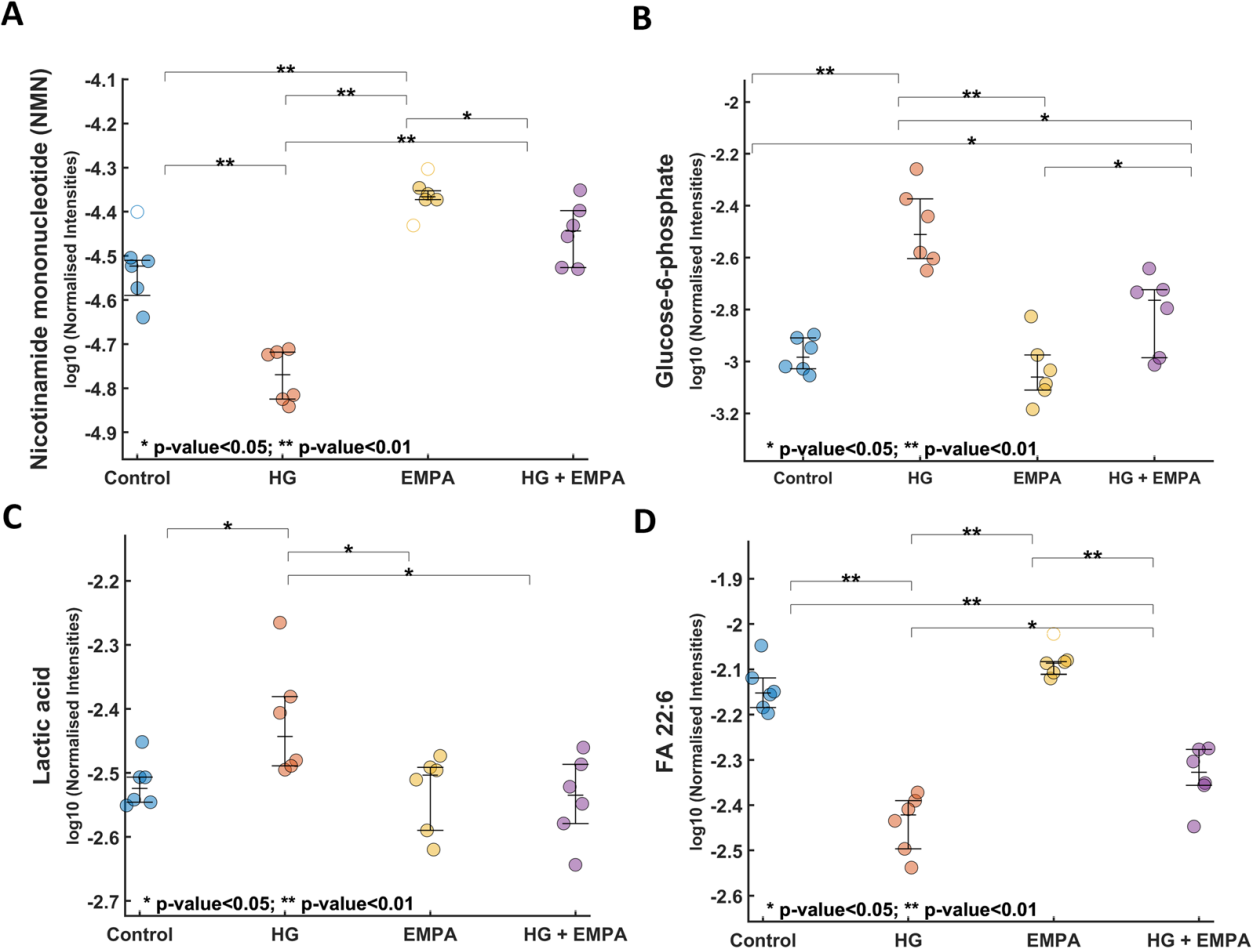
Dot-plots of nicotinamide mononucleotide, Glucose-6-phosphate, Lactic acid, FA 22:6. **A**–**D** The reported values refer to normalized data

**Fig. 5:**
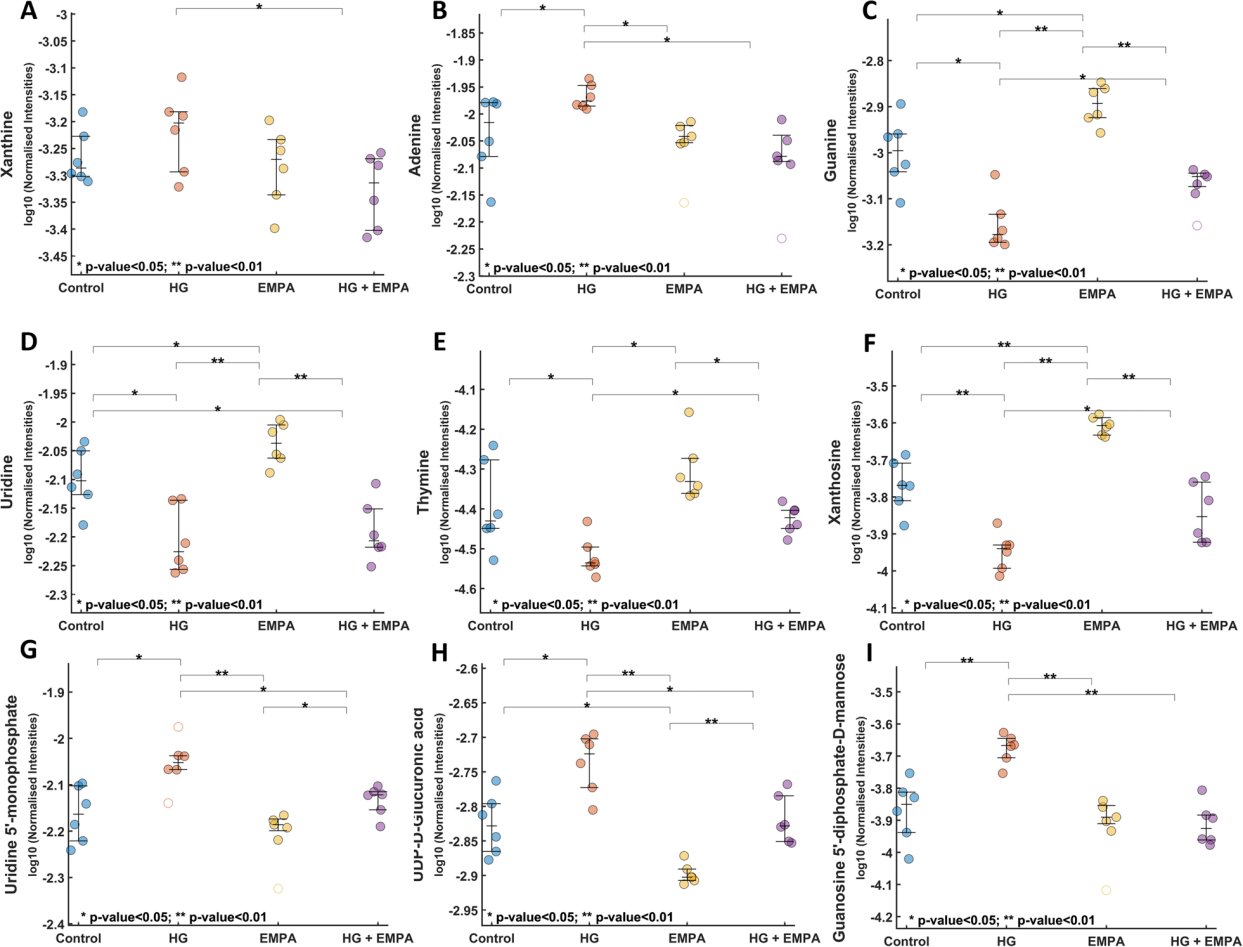
Dot-plots of xanthine, adenine, guanine, uridine, thymine, xanthosine, uridine 5′—monophosphate, UDP-D-glucuronic acid, and guanosine 5′—diphosphate-D-mannose. **A**–**I** The reported values refer to normalized data

Subsequently, we focused on significantly modulated compounds (p < 0.05) in comparing HG and HG + EMPA groups from ANOVA analysis. Concerning polar metabolome, nicotinamide mononucleotide (NMN, HMDB0000229) and glucose-6-phosphate (G6P, HMDB0001401) showed an opposite trend during HG condition (Fig. 4a, b), NMN level is reduced compared to the control, while G6P is significantly increased. Notably, EMPA treatment restores the levels of both metabolites, resulting in a return to the control condition and a reduction in lactate levels (HMDB0000190) (Fig. 4c). Long-chain fatty acids (LCFA), such as FA 22:6, FA 22:4, and 18:2, were notably reduced following the HG condition. In this case, the treatment with EMPA could significantly raise only the level of FA 22:6 (DHA, HMDB0002183) Fig. 4d. Among purines, HG increased the levels of purines xanthine (HMDB0000292) and adenine (HMDB0000034) while reduced guanine (HMDB0000132) as well as pyrimidine uridine (HMDB0000296) and thymine (HMDB0000262). Among nucleosides and nucleotides, xanthosine (HMDB0000299) was reduced, while on the contrary, nucleotide Uridine 5′—monophosphate (HMDB0000288) and the nucleotide sugars UDP-D-Glucuronic acid (HMDB0000935), and Guanosine 5′—diphosphate-D-mannose (HMDB0001163) had an opposite trend. Notably, EMPA treatment partially restored the level of all metabolites towards control (Fig. 5a–i).

### Lipidome remodeling in HG-treated AC-16 cells and EMPA influence

**Fig. 6:**
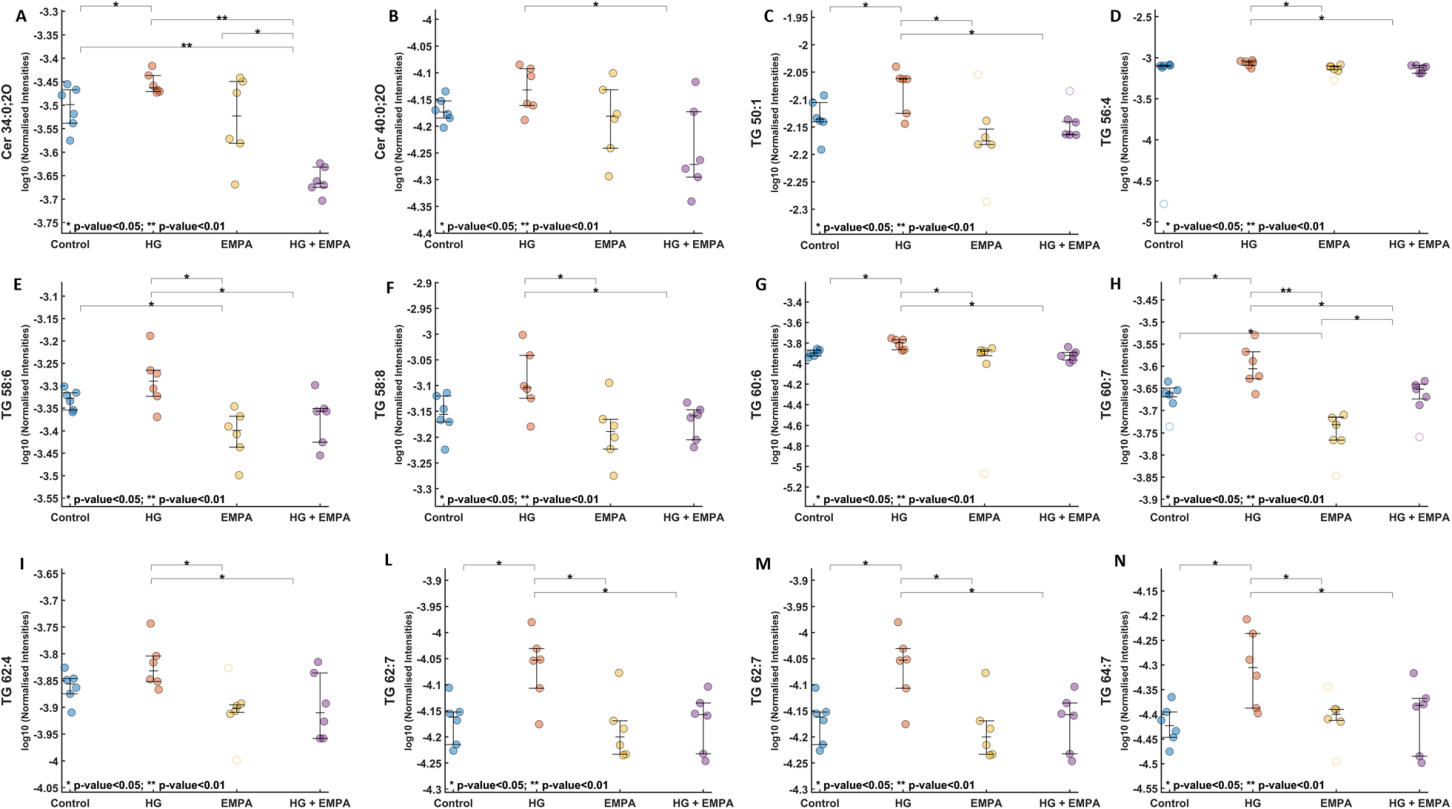
Dot-plots of Cer 34:0;O2, Cer 40:0;O2, TG 50:1, TG 56:4, TG 58:6, TG 58:8, TG 60:6, TG 60:7, TG 62:4, TG 62:7, TG 62:8, TG 64:7. A-N: The reported values refer to normalized data

Lipidome analysis showed that numerous lipid sub-classes were modulated following HG or EMPA treatment, such as Ceramides (Cer), Glycerophospholipids (PC, LPC, LPE, LPC-O, LPE-O, PG, and PI), Cholesterol Esters (CE), Triacylglycerols (TG). Among the significant lipids in both VIP and ANOVA results, sphingolipids such as two dihydro-ceramides emerged, namely Cer 34:0; O2 (HMDB0011760) and Cer 40:0;O2 (HMDB0011760), which were increased in HG conditions compared to control, while EMPA treatment was able to reduce their levels significantly. In addition, the levels of multiple TGs, with different chain lengths and several double bonds, were increased in HG group (TG 50:1, TG 56:4, TG 58:6, TG 58:8, TG 60:6, TG 60:7, TG 62:4, TG 62:7, TG 62:8, TG 64:7), interestingly, they were all reduced to HG conditions and reduced following EMPA treatment (Fig. 6a–n).

### Metabolic pathways associated with EMPA treatment in AC16 cells

**Fig. 7:**
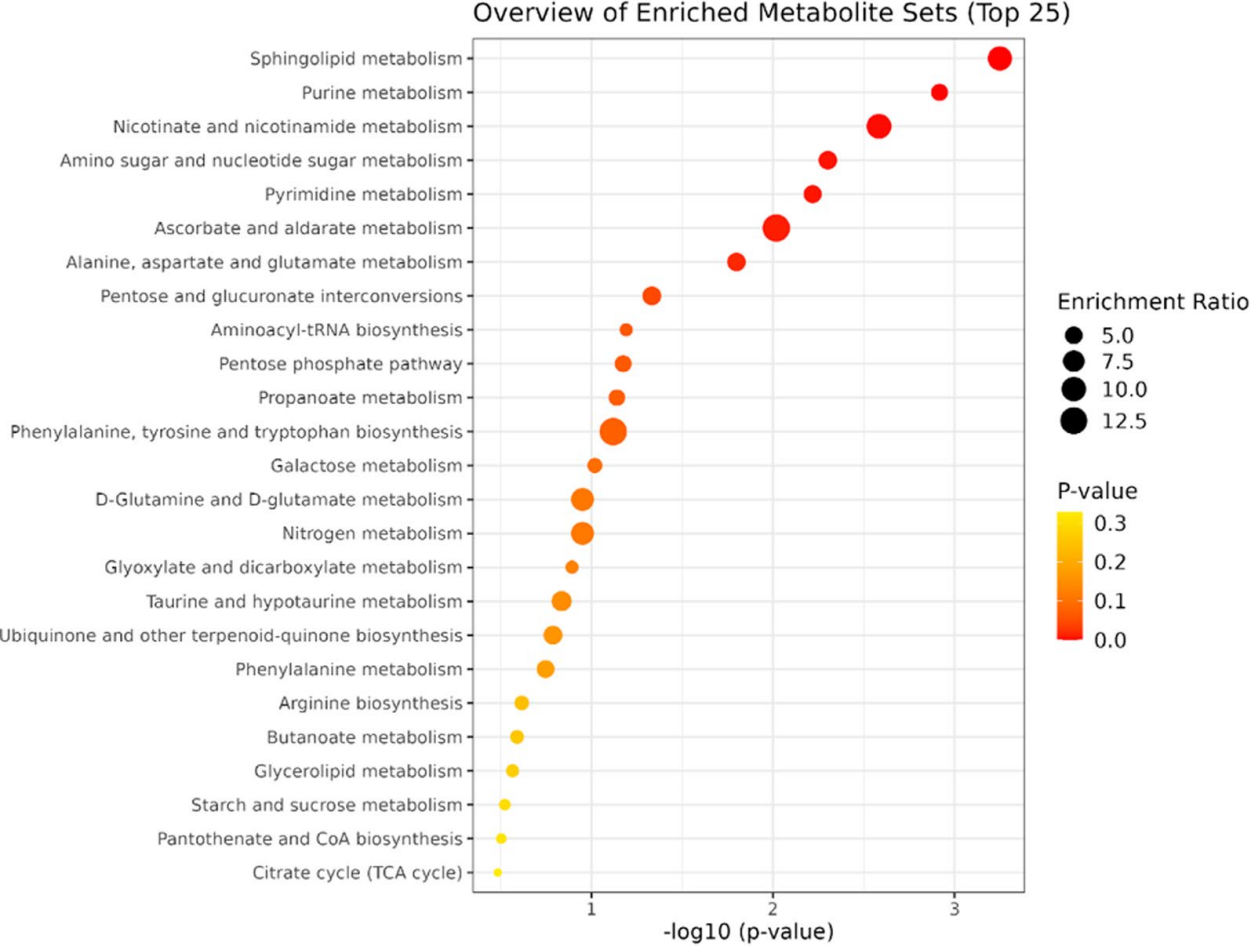
KEGG enrichment. The scatterplot shows the altered pathways of metabolites and lipids following EMPA treatment

Kyoto Encyclopedia of Genes and Genomes (KEGG) enrichment analysis of altered metabolites was conducted to clarify the correlation between metabolite changes and lipids with EMPA administration. The significantly modulated metabolites and lipids (p < 0.05) between HG and HG + EMPA treatments were used to build a KEGG over-representation analysis by selecting the hypergeometric test to evaluate if metabolites involved in a particular pathway are enriched to random hits (Fig. 7). Enrichment scatter plot showed that among the top five main significant pathways were: sphingolipids metabolism, purine and pyrimidine, nicotinate and nicotinamide metabolism, as well as nucleotide sugars metabolism, which underscores a profound metabolic reprogramming involving both cellular energy and lipid homeostasis.

### Significant variables for proteomics

**Fig. 8:**
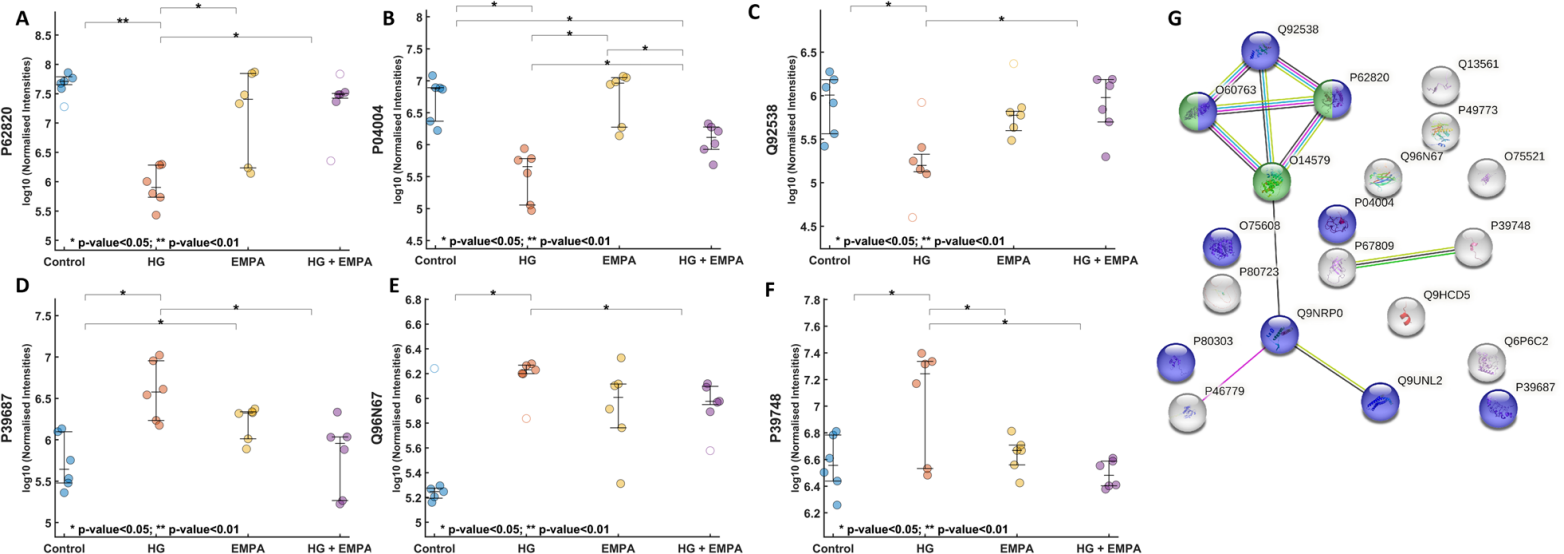
Ontology analysis. **A**–**G** Graphical representation of P62820 (Ras-related protein Rab-1A [OS = Homo sapiens]), P04004 (Vitronectin [OS = Homo sapiens]), Q92538 (Golgi-specific brefeldin A-resistance guanine nucleotide exchange factor 1 [OS = Homo sapiens]), Q96N67 (Dedicator of cytokinesis protein 7 [OS = Homo sapiens]), P39687 (Acidic leucine-rich nuclear phosphoprotein 32 family member A [OS = Homo sapiens]), P39748 (Flap endonuclease 1 [OS = Homo sapiens]). The reported values refer to normalized data. In the String pathway of VIP proteins, the blue nodes represent  ER derived proteins

Among the 20 significant VIP protein features extracted from the PLS-DA model, STRING and Gene Ontology analysis revealed as most enriched protein derivation in the endoplasmic reticulum (ER) and ER to golgi vesicle-mediated transport (p < 0.01). Notably, 9 out of 20 were components of ER and Golgi apparatus. Six proteins resulted in being significant (p < 0.05) from ANOVA comparison between HG and EMPA groups, namely P62820, P04004, P39748, Q96N67 Q92538, P39687, showing an opposite trend to HG following EMPA treatment (Fig. 8a–f). All selected proteins exhibit a marked change in expression after exposure to hyperglycemia (HG), with mean values of expression that differ from the control values. The treatment with EMPA alone or in conjunction with HG consistently displays the opposite pattern. EMPA can restore the expression levels of P62820, P04004, and Q92538, downregulated when high glucose is utilized as a stressor. On the other hand, following hyperglycemia treatment, P39687, Q96N67, and P39748 are overexpressed and are shut down by EMPA. However, it should be underlined that EMPA has a counterbalancing effect even if administered to cardiomyocytes without HG, proving its cardioprotective activity apart from the diabetic condition.

## Discussion

Our study in human cardiomyocytes, firstly demonstrates, by multi-omics analyses, that HG  treatment induces an alteration of metabolomic, lipidomic, and proteomic profiles compared to NG conditions and that EMPA treatment, by involving several molecular pathways, is able to restore the pre-HG condition (Graphical Abstract). In particular, EMPA was able to modulate and partially restore the levels of multiple metabolites associated with cellular stress, which are dysregulated in the HG conditions, such as nicotinamide mononucleotide, glucose-6-phosphate, lactic acid, FA 22:6 as well as nucleotide sugars and purine/pyrimidines. Additionally, EMPA regulated the levels of several lipid sub-classes, in particular dihydroceramide and triacylglycerols, which tend to accumulate in HG conditions resulting in lipotoxicity. Finally, EMPA counteracted the dysregulation of ER-derived proteins involved in cellular stress management.

Type 2 diabetes is a risk factor for heart failure (HF), and several clinical trials showed a positive correlation between hyperglycemia and an increased HF risk in patients with T2DM [30]. HF is regarded as an alteration of cardiac structure/function, resulting in inadequate cardiac output and increased left ventricular filling pressure [2, 31]. DCM, characterized by abnormal myocardial structure and performance, promotes cardiac fibrosis and stiffness, and therefore it is associated with diastolic dysfunction that often evolves into heart failure [4]. Several mechanisms have been demonstrated to clarify how diabetes promotes cardiac stiffness, hypertrophy, and fibrosis, leading to cardiac diastolic and systolic dysfunction and, therefore, HF development [7]. Mechanisms are related to impaired cardiac insulin metabolic signaling, mitochondria dysfunction, oxidative stress, AGEs, impairment of mitochondria Ca^2+^ handling, inflammation, activation of the renin–angiotensin–aldosterone system (RAAS), cardiomyocyte death, as well as microvascular dysfunction [4]. However, the central feature of HF is myocardial metabolic impairment, and NAD^+^-dependent pathways have a pivotal role in metabolism-driven disease progression [32, 33]. In our model, NMN, a key intermediate for NAD^+^ biosynthesis, was downregulated by high glucose condition and restored to basal concentration by EMPA treatment. NMN plays a fundamental role in replenishing the levels of NAD^+^ in the heart [34]. NAD^+^ is the primary coenzyme involved in fuel oxidation and oxidative phosphorylation. It is a substrate for enzymes involved in energy stress and oxidative stress response, and Sirtuin (SIRT) 1 is one of the most important enzymes [35]. In the murine and human failing heart biopsies, the observed reduction in NAD^+^ homeostasis and the activation of nicotinamide riboside kinase 2 (NMRK2 kinase), an enzyme involved in the phosphorylation of nicotinamide riboside precursor, was considered as events of DCM leading to heart failure [36].

Moreover, NAD^+^ redox imbalance is causal in systolic and diastolic dysfunction induced by metabolic stresses. NAD^+^ redox imbalance, via the increment of global protein acetylation and the phosphorylation of troponin-I (Tnl) at Serine 150 and the consequent impairment of energetics and oxidative stress, exacerbates contractile and relaxation function [37]. Thus, rescuing NAD^+^ metabolism by NMN could be a viable strategy to elevate NAD^+^ levels, mitigate NAD^+^ imbalance, and improve cardiac dysfunction in diabetic hearts [38].

So far, in the presence of high glucose concentrations, a depletion of NAD^+^ and SIRT1 is generated, which translates clinically into a reduced contractile capacity of the heart due to energy substrate deficiency which is associated with an increase in glucose-induced inflammatory aggression. Finally, hyperglycemia contributes to depleting the myocardium of energy substrates and to damage the cellular function of the cardiomyocyte.

Indeed, SGLT2i are, among anti-glycemic drugs, the only class that reduces the risk of HF in T2DM [39, 40]. Several trials have demonstrated that SGLT2i reduced the risk of HF hospitalizations in diabetic patients [41]. Experimental and clinical studies have described the beneficial effects of SGLT2i on restoring bioenergetic capacity and improving cardiac efficiency [42]. Interestingly, our results support the hypothesis that SGLT2i can exert beneficial effects in reversing cardiac damage by increasing the NAD ^+^/SIRT1 pathway in HF [3, 16] and are consistent with literature data demonstrating both the downregulation of SIRT1 under high glucose conditions, likely due to the activation of SGLT2 via GLUT2/importin-α1/ HNF- α1 [43], as well as the physical interactions between SIRT1-SGLT2 [44].

Moreover, in our model EMPA was also able to modulate and partially restore the levels of glucose-6-phosphate, lactic acid, FA 22:6 as well as nucleotide sugars and purine/pyrimidines. G6P is the first intermediate of glucose metabolism, which inside the cells is phosphorylated by hexokinase and covers a central role in energy metabolism. The cardiac phenotype is modified in heart failure due to glucose transporters GLUT1 and GLUT4 dysfunction [45]. The upregulation of GLUT1 and the downregulation of GLUT4 results in a suppression of glucose oxidation, consequently increasing the formation of cytotoxic glucose intermediates such as G6P and lactate [46]. The low levels of NMN and higher levels of G6P and lactate could suggest an impaired mitochondrial status, leading to deficient ATP synthesis resulting in cellular stress, which in turn is alleviated by EMPA treatment.

In diabetic rats, EMPA ameliorated the pronounced DCM reducing mitochondrial pleomorphic, impaired lipid metabolism, myocardial fibrosis, and associated diastolic and systolic functional impairment [12]. In HF conditions, increased glucose utilization can suppress LCFA metabolism and cytosol transport by downregulating their transporters [47]. In agreement, our results show that FA levels were reduced following HG treatment and that among FA, EMPA was able to increase only the level of FA 22:6, which is an essential ω-3 polyunsaturated fatty acid that has been positively associated with improved cardiovascular cellular metabolism; on the contrary, low levels of FA 22:6 were associated with insulin sensitivity [48]. Metabolomic profiling of the effects of dapagliflozin in heart failure with reduced ejection fraction was also evaluated in human plasma samples. Dapagliflozin increased ketone-related cluster, short-chain acylcarnitine, and medium-chain acylcarnitine compared with placebo. Increases in long-chain acylcarnitine, dicarboxylic-acylcarnitine, and aromatic amino acid metabolite clusters were associated with worse quality of life and increased NT-pro-BNP levels [49]. The dysregulation of purine metabolism is a hallmark of diabetes [50]. Our results revealed a complex modulation of purine/pyrimidines, and EMPA could revert the effects of HG, driving their levels like control. Previous metabolomics analyses performed on HUVEC cells and in patients treated with SGLT2 inhibitors have shown similar results, even with differences among different drugs such as canagliflozin, dapagliflozin, and  EMPA [51, 52].

We have previously demonstrated on human biopsy samples that diabetic cardiomyopathy is associated with lipid accumulation and lipotoxicity, and this phenomenon was reduced by concomitant therapy with SGLT2i [53]. It has been observed how SGLT2i acts, inducing significant lipid remodeling [54]. These aspects were evident in our model following HG and EMPA treatment. Ceramides, triacylglycerols, and generally glycerophospholipids (PC, PE, LPC, LPE) were the most modulated among the main lipid classes and sub-classes. Ceramides can be considered toxic lipid intermediates that increase ER stress, augmenting apoptosis. In particular, dihydroceramides are sphingolipids intermediates that can mediate oxidative and ER stress and have been recently positively linked to T2D and cardiovascular diseases (CVD) [55]. Strikingly these were among the top significant lipids increased by HG and reverted by EMPA. In addition, concerning sphingolipids, such as sphingoid bases, also dyhidrosphingosine, also known as sphinganine, was found elevated in HG conditions and reverted by EMPA (data not shown) which has been also associated to cardiac damage in human cardiomyocites [56].

Furthermore, many TG with different degrees of unsaturation of chain length showed higher levels in the HG condition. In this context, the accumulation of myocardial TGs levels has been associated with cardiac dysfunction such as cardiac steatosis, diabetes, and HF [57–59] and SGLT2i has already shown the ability to reduce plasma circulating TGs levels [60], and restore the level of TGs globally. On the other hand, a clear trend could not be evidenced for glycerophospholipids sub-classes by our study. So far, our data provide evidence that presence of EMPA in cardiac cell medium, promote a TG utilization in cardiac cell thus shifting the cardiomyocyte cell from a poorly to and more efficient ATP productive metabolism; in conclusion, EMPA promotes a positive reverse metabolic flexibility from glucose to lipid metabolism which per se is normally associate with more efficient cardiac functioning.

Proteomics results evidenced mainly a modulation and involvement of ER-associated proteins. In this regard, in addition to mitochondrial dysfunction, ER stress has been associated with diabetic cardiomyopathy and different anti-glycemic drugs classes have been reported to possess the ability to reduce ER stress, such as metformin, GLP-1 agonists as well as SGLT-2i [61–63]. Among statistically significant proteins, EMPA was able to revert the effect of HG-mediated reduction of the small GTPase Rab1A (RAB1A), which is involved in autophagy. Its downregulation has been associated with T2D progression and cardiac hypertrophy [64, 65], together with an effect on its potential regulator dedicator of cytokinesis (DOCK7) [66]. The beneficial cardioprotective effect of SGLT2 has also been attributed to autophagy regulation [67, 68]. In addition, EMPA was able to counteract the effects of HG condition, which induced the dysregulation of proteins involved in the mitochondrial DNA repair, such as Flap endonuclease 1 (FEN1), as well as proteins involved in cellular stress modulation such as acidic leucine-rich nuclear phosphoprotein 32 family member A (ANP32A) which was increased in HG condition and can promote apoptosis by the activation of caspase-9 (CASP9). Furthermore, golgi brefeldin A resistance factor 1 (GBF1), which is involved in mitochondrial homeostasis and dynamics, was reduced in HG cells. These findings agree with previous studies using knockdown models of both GBF1 and ANP32A associated with ER stress and cardiac hypertrophy [69, 70].

We acknowledge that results obtained only in vitro could be a potential limitation of our study. However, primary human cardiomyocytes are challenging to get, limited in numbers, and cannot be maintained in culture for more than a few days. Rodent cardiomyocytes have a structure comparable to the human heart, but there are mounting concerns that their function and gene expression differ. Prior research on diabetic cardiomyopathy broadly used animal models and human plasma samples. Still, in the first case, animal models do not accurately mimic the human condition, and in the second, only systemic effects, not cell-direct ones, should be shown in plasma. Because of all these evaluations, we decided to employ the AC16 cellular model in our research. As previously reported, these cells are ideal for in vitro study in both standard and pathological conditions since they have kept the genetics of primary cardiomyocytes [64]. Additionally, AC16 cells are simple to maintain, readily available in large commercial settings, and can be cultivated by standardized methods. These characteristics make it easier to maintain strict control over the chemical and physical environment, such as those employed in our investigation to examine the impact of hyperglycemia and/or the usage of EMPA.

A further particular aspect of our study is that an amount of 33 mmol of glucose is a very unusual plasma glucose levels, not comparable with the concept of hyperglycemia in diabetic patients especially if we consider that the effect of hyperglycemia in the diabetic patients is time-dependent and even lower but persisting concentrations of glucose for many months or years can damage cardiac cells. Indeed, 33 mmol is widely used in the laboratory to cause cell injury by a short-term glucose exposure and is the only option to mimic cardiac damage in an in vitro model.

Moreover, another potential limitation of our study might be identified in our choice not to incorporate a fourth group of cells pre-treated with EMPA before exposure to hyperglycemic conditions. Indeed, including such a group could provide additional insights into a "preventive" mechanism of EMPA action under these circumstances. Nevertheless, the study of the preventive effect of EMPA needs a more complex study design, specifically focused on the effect per se of the drug encompassing a dose–effect curve to highlight the more efficient dose and the toxic one.

In addition, the set of proposed molecular mechanisms derives from an exposure of AC16 cells to hyperglycemia and/or empagliflozin. However, even though the metabolic disturbance induced at the cellular level by hyperglycemia has been demonstrated with a cause-effect relationship, the induction of the studied metabolic pathways with omics requires more precise validation for a clear cause-effect determinism. Therefore, further studies will be necessary to differentiate the cellular perturbation effect of hyperglycemia or empagliflozin from the cascading consequences on other metabolic cycles.

## Conclusions

The present study combined omics analysis, revealing that SGLT2i treatment ameliorated HG-induced changes in crucial biomolecules. Even though our study provides only in vitro results, it should be pointed out that our data might be extended to human patients since it is widely known that the development of heart failure is mainly a cardio-metabolic problem deriving and combined to a reduced mechanic capacity of the heart to pump blood in the periphery in the due amount. Long-lasting hyperglycemia contributes to derange such a mechanism, inducing a reduced myocardial wall elasticity (as for heart failure with preserved ejection fraction as diabetic cardiomyopathy). So far, EMPA could prompt intracellular metabolic pathways, which could help the myocardial wall to increase ATP production and improve wall myocardial contraction in terms of power and elasticity. Therefore, our data enable us to understand the metabolic pathways that can lead to the development of diabetic heart disease and, at the same time, facilitate the identification of a therapeutic pathway useful for preventing its development.

### Supplementary Information

**Additional file 1: Table S1.** Annotated metabolites by HILIC-HRMS/MS**. Table S2.** Annotated lipids by RP-UHPLC-TIMS/MS. **Table S3.** Annotated proteins by nano-LC-HRMS. **Table S4.** Values of PLS-DA performances. It reports the values of accuracy, sensitivity and specificity for the training set. Model built with all the modality independently. 4 repetitions were used as a training set. **Table S5.** Values of PLS-DA performances. It reports the values of accuracy, sensitivity and specificity for the cross-validation (leave one replicate out). Model built with all the modality independently. **Table S6.** Values of PLS-DA performances. It reports the values of accuracy, sensitivity and specificity for the test set. Model built with all the modality independently. Two repetitions were used as an independent test set. **Figure S1.** Graphical representation of the 20 most important molecules for classification obtained from the analysis of the VIP scores of PLS-DA for the lipidomics dataset. **Figure S2.** Graphical representation of the 20 most important molecules for classification obtained from the analysis of the VIP scores of PLS-DA for the metabolomics dataset. **Figure S3.** Graphical representation of the 20 most important molecules for classification obtained from the analysis of the VIP scores of PLS-DA for the Proteomics dataset.

## Data Availability

The data used and/or analyzed during the current study are available from the corresponding author on reasonable request.

## Abbreviations

AGEs: Advanced glycation end products
ANP32A: Acidic leucine-rich nuclear phosphoprotein 32 family member A
CASP9: Caspase-9
CE: Cholesterol Esters
Cer: Ceramides
CVD: Cardiovascular diseases
DCM: Diabetic cardiomyopathy
DOCK7: Dedicator of cytokinesis
ECM: Extracellular matrix
EMPA: Empagliflozin
ER: Endoplasmic reticulum
FA: Fatty acids
FEN1: Flap endonuclease 1
G6P: Glucose-6-phosphate
GBF1: Golgi brefeldin A resistance factor 1
HF: Heart failure
KEGG: Kyoto Encyclopedia of Genes and Genomes
LCFA: Long-chain fatty acids
NMN: Nicotinamide mononucleotide
NMRK2: Kinase nicotinamide riboside kinase 2
PCA: Principal component analysis
RAAS: Renin–angiotensin–aldosterone system
RAB1A: GTPase Rab1A
RNS: Reactive nitrogen species
ROS: Reactive oxygen species
SGLT2i: Sodium-glucose cotransporter-2 inhibitors
Sirtuin: SIRT1
T2DM: Type 2 diabetes mellitus
TG: Triacylglycerols
Tnl: Troponin-I
