# Supplementary material for: Multi-omics analysis reveals attenuation of cellular stress by empagliflozin in high glucose-treated human cardiomyocytes
Source: J Transl Med. 2023 Sep 23;21:662. doi: 10.1186/s12967-023-04537-1 (PMC10518098; doi:10.1186/s12967-023-04537-1)
Supplement: Supplementary file 1 — Additional file 1: Table S1. Annotated metabolites by HILIC-HRMS/MS. Table S2. Annotated lipids by RP-UHPLC-TIMS/MS. Table S3. Annotated proteins by nano-LC-HRMS. Table S4. Values of PLS-DA performances. It reports the values of accuracy, sensitivity and specificity for the training set. Model built with all the modality independently. 4 repetitions were used as a training set. Table S5. Values of PLS-DA performances. It reports the values of accuracy, sensitivity and specificity for the cross-validation (leave one replicate out). Model built with all the modality independently. Table S6. Values of PLS-DA performances. It reports the values of accuracy, sensitivity and specificity for the test set. Model built with all the modality independently. Two repetitions were used as an independent test set. Figure S1. Graphical representation of the 20 most important molecules for classification obtained from the analysis of the VIP scores of PLS-DA for the lipidomics dataset. Figure S2. Graphical representation of the 20 most important molecules for classification obtained from the analysis of the VIP scores of PLS-DA for the metabolomics dataset. Figure S3. Graphical representation of the 20 most important molecules for classification obtained from the analysis of the VIP scores of PLS-DA for the Proteomics dataset. [file 12967_2023_4537_MOESM1_ESM.docx]

**Supplementary material for the manuscript:**

**Multi-omics analysis reveals attenuation of cellular stress by Empagliflozin in High Glucose-treated human cardiomyocytes.**

Lucia Scisciola^a1^, Ugo Chianese^b1^, Vicky Caponigro^c1^, Manuela Giovanna Basilicata^c^, Emanuela Salviati^c^, Lucia Altucci^b,d,e,f^, Pietro Campiglia^c^, Giuseppe Paolisso^a,g^, Michelangela Barbieri^a*^, Rosaria Benedetti^b, f#^, Eduardo Sommella^c#^

**Table S1:** Annotated metabolites by HILIC-HRMS/MS

| **Rt(min)** | **m/z** | **Metabolite annotation** | **Adduct** | **Molecular Formula** |
| --- | --- | --- | --- | --- |
| 7.416 | 159.07664 | (S)-DIHYDROOROTATE | [M+H]+ | C5H6N2O4 |
| 1.305 | 187.13345 | 10-Hydroxydecanoic acid | [M-H]- | C10H20O3 |
| 4.35 | 102.06647 | 1-AMINOCYCLOPROPANE-1-CARBOXYLATE | [M+H]+ | C4H7NO2 |
| 8.694 | 137.07066 | 1-Methylnicotinamide | [M]+ | C7H9N2O |
| 1.296 | 219.11301 | 1-Methyltryptophan | [M+H]+ | C12H14N2O2 |
| 13.243 | 413.26645 | 2-acetoxy-4-pentadecylbenzoic acid | [M+Na]+ | C24H38O4 |
| 1.191 | 213.96326 | 2-Benzothiazolesulfonic acid | [M-H]- | C7H5NO3S2 |
| 6.722 | 332.07574 | 2'-Deoxyadenosine-5'-monophosphate | [M+H]+ | C10H14N5O6P |
| 6.689 | 103.03896 | 3-HYDROXYBUTANOIC ACID | [M-H]- | C4H8O3 |
| 1.492 | 129.05458 | 4-Acetylbutyric acid | [M-H]- | C6H10O3 |
| 5.192 | 144.04449 | 4-Hydroxyquinoline | [M-H]- | C9H7NO |
| 8.775 | 160.13318 | 5-Aminovaleric acid betaine; CE10; CDLVFVFTRQPQFU-UHFFFAOYSA-N | [M+H]+ | C8H17NO2 |
| 7.901 | 118.08639 | 5-Aminovaleric acid; CE10; JJMDCOVWQOJGCB-UHFFFAOYSA-N | [M+H]+ | C5H11NO2 |
| 1.567 | 298.09625 | 5'-S-Methylthioadenosine; LC-ESI-QTOF; MS2; CE | [M+H]+ | C11H15N5O3S |
| 6.741 | 204.12291 | Acetylcarnitine | [M]+ | C9H18NO4 |
| 8.432 | 146.11763 | Acetylcholine; LC-ESI-QTOF; MS2; CE | [M]+ | C7H16NO2 |
| 1.549 | 134.04613 | Adenine | [M-H]- | C5H5N5 |
| 4.817 | 268.10339 | Adenosine | [M+H]+ | C10H13N5O4 |
| 6.904 | 428.03629 | Adenosine_Diphosphate; LC-ESI-QTOF; MS2; CE | [M+H]+ | C10H15N5O10P2 |
| 6.833 | 346.0556 | Adenosine-3-monophosphate | [M-H]- | C10H14N5O7P |
| 7.04 | 90.05542 | Alanine | [M+H]+ | C3H7NO2 |
| 7.005 | 160.06064 | ALPHA-AMINOADIPATE | [M-H]- | C6H11NO4 |
| 7.648 | 291.12961 | Argininosuccinic acid; CE10; KDZOASGQNOPSCU-WDSKDSINSA-N | [M+H]+ | C10H18N4O6 |
| 7.249 | 133.06082 | Asparagine; LC-ESI-QTOF; MS2; CE | [M+H]+ | C4H8N2O3 |
| 7.142 | 261.07281 | Asp-Glu | [M-H]- | C9H14N2O7 |
| 6.405 | 281.1127 | Asp-Phe | [M+H]+ | C13H16N2O5 |
| 6.447 | 187.09697 | Azelaic acid (Not validated); PlaSMA ID-221 | [M-H]- | C9H16O4 |
| 1.164 | 90.05539 | beta-Alanine | [M+H]+ | C3H7NO2 |
| 8.385 | 335.06424 | beta-Nicotinamide mononucleotide; LC-ESI-QTOF; MS2; CE | [M+H]+ | C11H15N2O8P |
| 6.002 | 232.15477 | Butyryl carnitine (isomer of 920); PlaSMA ID-919 | [M+H]+ | C11H21NO4 |
| 8.615 | 227.11423 | Carnosine | [M+H]+ | C9H14N4O3 |
| 0.824 | 369.35172 | Cholesterol | [M+H-H2O]- | C27H46O |
| 7.416 | 176.10289 | Citrulline; LC-ESI-QTOF; MS2; CE | [M+H]+ | C6H13N3O3 |
| 5.237 | 114.06628 | Creatinine | [M+H]+ | C4H7N3O |
| 5.904 | 211.14435 | Cyclo(proline-leucine) | [M+H]+ | C11H18N2O2 |
| 8.539 | 223.07483 | Cystathionine | [M+H]+ | C7H14N2O4S |
| 6.746 | 179.04883 | Cysteinylglycine; LC-ESI-QTOF; MS2; CE | [M+H]+ | C5H10N2O3S |
| 7.285 | 404.02628 | CYTIDINE 5'-DIPHOSPHATE | [M+H]+ | C9H15N3O11P2 |
| 7.498 | 489.11401 | Cytidine 5'-diphosphocholine; LC-ESI-QTOF; MS2; CE | [M+H]+ | C14H26N4O11P2 |
| 7.282 | 324.05872 | Cytidine 5'-monophosphate; LC-ESI-QTOF; MS2; CE | [M+H]+ | C9H14N3O8P |
| 5.127 | 112.05086 | Cytosine | [M+H]+ | C4H5N3O |
| 5.082 | 180.08826 | D-(+)-Glucosamine; LC-ESI-QTOF; MS2; CE | [M+H]+ | C6H13NO5 |
| 5.873 | 218.10301 | D-(+)-Pantothenic acid; LC-ESI-QTOF; MS2; CE | [M-H]- | C9H17NO5 |
| 6.98 | 162.07629 | D-2-Aminoadipic acid; LC-ESI-QTOF; MS2; CE | [M+H]+ | C6H11NO4 |
| 6.642 | 161.09215 | D-Ala-D-ala | [M+H]+ | C6H12N2O3 |
| 7.233 | 179.05534 | D-FRUCTOSE | [M-H]- | C6H12O6 |
| 6.793 | 193.035 | D-GLUCURONIC ACID | [M-H]- | C6H10O7 |
| 5.336 | 302.30484 | Dihydrosphingosine | [M+H]+ | C18H39NO2 |
| 5.779 | 181.05 | DL-3-(4-Hydroxyphenyl)lactic acid; LC-ESI-QTOF; MS2; CE | [M-H]- | C9H10O4 |
| 5.887 | 132.10204 | DL-Norleucine; CE10; LRQKBLKVPFOOQJ-UHFFFAOYSA-N | [M+H]+ | C6H13NO2 |
| 6.165 | 146.04482 | DL-threo-beta-Methylaspartic acid; LC-ESI-QTOF; MS2; CE | [M-H]- | C5H9NO4 |
| 0.988 | 327.23303 | Docosahexanoic acid | [M-H]- | C22H32O2 |
| 0.953 | 331.26431 | Docosatetraenoic acid | [M-H]- | C22H36O2 |
| 7.103 | 229.0114 | D-Ribose 5-phosphate | [M-H]- | C5H11O8P |
| 1.067 | 297.24374 | FA 18:1+1O; PlaSMA ID-585 | [M-H]- | C18H34O3 |
| 1.316 | 171.10181 | FA 9:1+1O; PlaSMA ID-163 | [M-H]- | C9H16O3 |
| 6.544 | 279.10092 | gamma-Glutamylmethionine | [M+H]+ | C10H18N2O5S |
| 7.227 | 259.02255 | Glucose-6-phosphate | [M-H]- | C6H13O9P |
| 7.168 | 147.07628 | Glutamine | [M+H]+ | C5H10N2O3 |
| 6.42 | 295.12851 | Glutamylphenylalanine (isomer of 1503); PlaSMA ID-1504 | [M+H]+ | C14H18N2O5 |
| 6.748 | 306.07614 | Glutathione | [M-H]- | C10H17N3O6S |
| 7.399 | 613.16089 | Glutathione (oxidized) | [M+H]+ | C20H32N6O12S2 |
| 6.78 | 247.09299 | Glu-Thr | [M-H]- | C9H16N2O6 |
| 6.537 | 105.01817 | GLYCERATE | [M-H]- | C3H6O4 |
| 7.379 | 76.03982 | GLYCINE | [M+H]+ | C2H5NO2 |
| 6.556 | 75.00758 | Glycolic acid | [M-H]- | C2H4O3 |
| 7.274 | 173.09241 | Glycyl-L-proline; LC-ESI-QTOF; MS2; CE | [M+H]+ | C7H12N2O3 |
| 5.798 | 150.04114 | Guanine | [M-H]- | C5H5N5O |
| 7.422 | 444.03137 | GUANOSINE 5'-DIPHOSPHATE | [M+H]+ | C10H15N5O11P2 |
| 7.076 | 604.07135 | Guanosine 5'-diphosphate-D-mannose | [M-H]- | C16H25N5O16P2 |
| 6.935 | 588.07666 | Guanosine 5'-diphospho-beta-L-fucose | [M-H]- | C16H25N5O15P2 |
| 7.354 | 362.05014 | Guanosine 5'-monophosphate; LC-ESI-QTOF; MS2; CE | [M-H]- | C10H14N5O8P |
| 6.028 | 282.08417 | Guanosine; LC-ESI-QTOF; MS2; CE | [M-H]- | C10H13N5O5 |
| 0.992 | 269.24844 | Heptadecanoic acid | [M-H]- | C17H34O2 |
| 5.315 | 178.05009 | HIPPURATE | [M-H]- | C9H9NO3 |
| 7.233 | 161.04453 | Hydroxymethylglutaric acid; PlaSMA ID-124 | [M-H]- | C6H10O5 |
| 7.047 | 132.07645 | Hydroxyproline | [M+H]+ | C5H9NO3 |
| 7.207 | 110.02724 | HYPOTAURINE | [M+H]+ | C2H7NO2S |
| 5.174 | 135.02998 | Hypoxanthine | [M-H]- | C5H4N4O |
| 5.885 | 146.06024 | Indole-3-carboxyaldehyde; LC-ESI-QTOF; MS2; CE | [M+H]+ | C9H7NO |
| 5.676 | 267.07343 | Inosine; LC-ESI-QTOF; MS2; CE | [M-H]- | C10H12N4O5 |
| 7.081 | 347.03934 | Inosine-5'-monophosphate; LC-ESI-QTOF; MS2; CE | [M-H]- | C10H13N4O8P |
| 7.127 | 260.16071 | Isoleucylglutamine; PlaSMA ID-1143 | [M+H]+ | C11H21N3O4 |
| 5.504 | 243.1711 | Isoleucylisoleucine; PlaSMA ID-377 | [M-H]- | C12H24N2O3 |
| 6.68 | 129.01813 | Itaconate | [M-H]- | C5H6O4 |
| 6.955 | 120.0657 | L-(-)-Threonine | [M+H]+ | C4H9NO3 |
| 2.078 | 104.0709 | L-2-Aminobutyric acid; LC-ESI-QTOF; MS2; CE | [M+H]+ | C4H9NO2 |
| 6.432 | 89.02319 | Lactic acid | [M-H]- | C3H6O3 |
| 6.906 | 132.02927 | L-ASPARTATE | [M-H]- | C4H7NO4 |
| 3.757 | 161.09213 | L-beta-Homolysine | [M+H]+ | C7H16N2O2 |
| 1.671 | 134.08122 | L-beta-Homothreonine; LC-ESI-QTOF; MS2; CE | [M+H]+ | C5H11NO3 |
| 7.596 | 162.11209 | L-Carnitine; LC-ESI-QTOF; MS2; CE | [M+H]+ | C7H15NO3 |
| 5.496 | 245.1859 | Leucylleucine; PlaSMA ID-1025 | [M+H]+ | C12H24N2O3 |
| 6.871 | 146.04489 | L-Glutamic acid; LC-ESI-QTOF; MS2; CE | [M-H]- | C5H9NO4 |
| 7.224 | 156.07671 | L-Histidine; LC-ESI-QTOF; MS2; CE | [M+H]+ | C6H9N3O2 |
| 0.983 | 279.23343 | Linoleic acid | [M-H]- | C18H32O2 |
| 5.487 | 520.34106 | LPC 18:2; PlaSMA ID-2753 | [M+H]+ | C26H50NO7P |
| 5.385 | 452.27917 | LPE 16:0; PlaSMA ID-1224 | [M-H]- | C21H44NO7P |
| 5.369 | 478.29498 | LPE 18:1; PlaSMA ID-1334 | [M-H]- | C23H46NO7P |
| 1.671 | 116.07078 | L-PROLINE | [M+H]+ | C5H9NO2 |
| 6.316 | 180.06581 | L-Tyrosine; LC-ESI-QTOF; MS2; CE | [M-H]- | C9H11NO3 |
| 6.264 | 145.0975 | Lysine | [M-H]- | C6H14N2O2 |
| 7.422 | 259.02255 | Mannose 1-phosphate | [M-H]- | C6H13O9P |
| 6.106 | 150.05835 | Methionine | [M+H]+ | C5H11NO2S |
| 7.102 | 166.05359 | Methioninesulfoxide | [M+H]+ | C5H11NO3S |
| 1.023 | 227.20149 | Myristic acid | [M-H]- | C14H28O2 |
| 6.444 | 198.08757 | N,N-Acetylhistidine; CE10; KBOJOGQFRVVWBH-ZETCQYMHSA-N | [M+H]+ | C8H11N3O3 |
| 13.054 | 203.15005 | N,N-Dimethylarginine | [M+H]+ | C8H18N4O2 |
| 5.324 | 230.2478 | N,N-Dimethyldodecylamine N-oxide | [M+H]+ | C14H31NO |
| 5.082 | 312.1301 | N2,N2-Dimethylguanosine | [M+H]+ | C12H17N5O5 |
| 7.09 | 217.12959 | N-Acetylarginine; CE10; SNEIUMQYRCDYCH-LURJTMIESA-N | [M+H]+ | C8H16N4O3 |
| 6.632 | 174.03973 | N-Acetylaspartic acid | [M-H]- | C6H9NO5 |
| 5.586 | 162.02202 | N-Acetylcysteine | [M-H]- | C5H9NO3S |
| 5.35 | 190.05383 | N-ACETYL-DL-METHIONINE | [M-H]- | C7H13NO3S |
| 6.374 | 146.0448 | N-ACETYL-DL-SERINE | [M-H]- | C5H9NO4 |
| 6.609 | 188.05583 | N-Acetylglutamate | [M-H]- | C7H11NO5 |
| 6.565 | 308.0983 | N-Acetylneuraminic acid; LC-ESI-QTOF; MS2; CE | [M-H]- | C11H19NO9 |
| 6.829 | 664.11768 | NAD+; LC-ESI-QTOF; MS2; CE | [M]+ | C21H28N7O14P2 |
| 5.398 | 176.03777 | N-Formyl-L-Methionine | [M-H]- | C6H11NO3S |
| 6.549 | 290.08887 | N-Fructosyl pyroglutamate; PlaSMA ID-534 | [M-H]- | C11H17NO8 |
| 1.427 | 123.05515 | Nicotinamide | [M+H]+ | C6H6N2O |
| 6.298 | 130.08643 | N-Methylproline | [M+H]+ | C6H11NO2 |
| 0.863 | 282.27893 | Oleamide | [M+H]+ | C18H35NO |
| 13.044 | 281.24881 | Oleic acid | [M-H]- | C18H34O2 |
| 1.005 | 255.23262 | Palmitic acid (NMR) | [M-H]- | C16H32O2 |
| 5.419 | 400.34338 | Palmitoylcarnitine | [M+H]+ | C23H45NO4 |
| 1.456 | 206.13884 | Panthenol | [M+H]+ | C9H19NO4 |
| 5.874 | 220.11766 | Pantothenic acid | [M+H]+ | C9H17NO5 |
| 1.014 | 241.21677 | Pentadecanoic acid | [M-H]- | C15H30O2 |
| 5.779 | 166.08606 | Phenylalanine; PlaSMA ID-359 | [M+H]+ | C9H11NO2 |
| 10.521 | 184.07314 | Phosphocholine; LC-ESI-QTOF; MS2; CE | [M]+ | C5H15NO4P |
| 7.239 | 166.97415 | Phosphoenolpyruvic acid; LC-ESI-QTOF; MS2; CE | [M-H]- | C3H5O6P |
| 1.724 | 137.02332 | P-HYDROXYBENZOIC ACID | [M-H]- | C7H6O3 |
| 6.974 | 97.02869 | Propionic acid | [M+Na]+ | C3H6O2 |
| 2.004 | 166.05003 | Pyridoxal; LC-ESI-QTOF; MS2; CE | [M-H]- | C8H9NO3 |
| 6.869 | 130.04984 | L-Pyroglutamic acid; CE10; ODHCTXKNWHHXJC-VKHMYHEASA-N | [M+H]+ | C5H7NO3 |
| 7.169 | 130.04987 | Pyroglutamic acid (not validated, isomer of 89); PlaSMA ID-88 | [M+H]+ | C5H7NO3 |
| 5.364 | 87.00758 | Pyruvic acid | [M-H]- | C3H4O3 |
| 1.096 | 164.0739 | R-3-Amino-5-(methylthio)pentanoic acid; LC-ESI-QTOF; MS2; CE | [M+H]+ | C6H13NO2S |
| 6.662 | 385.12823 | S-Adenosyl-homocysteine | [M+H]+ | C14H20N6O5S |
| 7.351 | 104.03412 | Serine | [M-H]- | C3H7NO3 |
| 6.167 | 219.13387 | Serylleucine; PlaSMA ID-819 | [M+H]+ | C9H18N2O4 |
| 7.792 | 258.10971 | sn-Glycero-3-phosphocholine; LC-ESI-QTOF; MS2; CE | [M]+ | C8H21NO6P |
| 5.212 | 300.2901 | Sphingosine | [M+H]+ | C18H37NO2 |
| 6.701 | 117.01822 | SUCCINIC ACID | [M-H]- | C4H6O4 |
| 12.979 | 189.1348 | Targinine | [M+H]+ | C7H16N4O2 |
| 6.908 | 124.00639 | TAURINE | [M-H]- | C2H7NO3S |
| 6.221 | 265.11133 | Thiamine; LC-ESI-QTOF; MS2; CE | [M]+ | C12H17N4OS |
| 6.554 | 135.02878 | Threonic acid | [M-H]- | C4H8O5 |
| 6.809 | 401.01517 | Thymidine 5'-diphosphate; LC-ESI-QTOF; MS2; CE | [M-H]- | C10H16N2O11P2 |
| 6.687 | 321.04971 | Thymidine-5'-monophosphate; LC-ESI-QTOF; MS2; CE | [M-H]- | C10H15N2O8P |
| 1.754 | 127.05025 | Thymine; LC-ESI-QTOF; MS2; CE | [M+H]+ | C5H6N2O2 |
| 0.976 | 281.24884 | Trans-Vaccenic acid | [M-H]- | C18H34O2 |
| 1.031 | 213.18546 | Tridecanoic acid | [M-H]- | C13H26O2 |
| 7.708 | 76.07625 | Trimethylamine N-oxide; CE30; UYPYRKYUKCHHIB-UHFFFAOYSA-N | [M+H]+ | C3H9NO |
| 5.894 | 203.08177 | Tryptophan; LC-ESI-ITFT; MS2; HCD; CE 70.0 eV; [M-H]- | [M-H]- | C11H12N2O2 |
| 6.738 | 565.04889 | UDP-D-glucose; LC-ESI-QTOF; MS2; CE | [M-H]- | C15H24N2O17P2 |
| 6.894 | 579.02588 | UDP-D-Glucuronic acid; LC-ESI-QTOF; MS2; CE | [M-H]- | C15H22N2O18P2 |
| 6.703 | 535.039 | UDP-xylose; LC-ESI-QTOF; MS2; CE | [M-H]- | C14H22N2O16P2 |
| 1.053 | 185.15419 | Undecanoic acid | [M-H]- | C11H22O2 |
| 5.226 | 111.01881 | URACIL | [M-H]- | C4H4N2O2 |
| 6.468 | 167.02016 | URATE | [M-H]- | C5H4N4O3 |
| 5.228 | 243.06244 | Uridine | [M-H]- | C9H12N2O6 |
| 7.001 | 405.00989 | Uridine 5_-(trihydrogen diphosphate) | [M+H]+ | C9H14N2O12P2 |
| 7.055 | 402.99484 | Uridine 5'-diphosphate | [M-H]- | C9H14N2O12P2 |
| 6.643 | 606.0755 | Uridine 5'-diphospho-N-acetylglucosamine; LC-ESI-QTOF; MS2; CE | [M-H]- | C17H27N3O17P2 |
| 6.983 | 323.02832 | Uridine 5'-monophosphate; LC-ESI-QTOF; MS2; CE | [M-H]- | C9H13N2O9P |
| 6.635 | 608.08868 | Uridine-5-diphosphoacetylgalactosamine | [M+H]+ | C17H27N3O17P2 |
| 6.348 | 139.05034 | Urocanic acid; LC-ESI-QTOF; MS2; CE | [M+H]+ | C6H6N2O2 |
| 6.43 | 118.08635 | Valine; LC-ESI-ITFT; MS2; CE 5.0 eV; [M+H]+ | [M+H]+ | C5H11NO2 |
| 5.335 | 151.02502 | Xanthine; LC-ESI-QTOF; MS2; CE | [M-H]- | C5H4N4O2 |
| 6.431 | 283.06827 | Xanthosine; LC-ESI-QTOF; MS2; CE | [M-H]- | C10H12N4O6 |

**Table S2:** Annotated lipids by RP-UHPLC-TIMS/MS

| **RT [min]** | **m/z meas.** | **Lipid annotation** | **Adduct** | **Molecular Formula** | **CCS (Å²)** |
| --- | --- | --- | --- | --- | --- |
| 3.29 | 642.6176 | CE 16:0 | [M+NH4]+ | C43H76O2 | 283.8 |
| 3.22 | 640.60257 | CE 16:1 | [M+NH4]+ | C43H74O2 | 283.2 |
| 3.35 | 670.64783 | CE 18:0 | [M+NH4]+ | C45H80O2 | 289 |
| 3.29 | 668.63239 | CE 18:1 | [M+NH4]+, [M+Na]+ | C45H78O2 | 287.1 |
| 3.31 | 682.64953 | CE 19:1 | [M+NH4]+ | C46H80O2 | 291 |
| 3.35 | 696.66247 | CE 20:1 | [M+NH4]+ | C47H82O2 | 293.6 |
| 3.24 | 692.63418 | CE 20:3 | [M+NH4]+ | C47H78O2 | 294.1 |
| 3.19 | 690.61776 | CE 20:4 | [M+NH4]+ | C47H76O2 | 292.5 |
| 3.4 | 724.69767 | CE 22:1 | [M+NH4]+ | C49H86O2 | 300.3 |
| 3.35 | 722.67932 | CE 22:2 | [M+NH4]+ | C49H84O2 | 300.2 |
| 3.3 | 720.66519 | CE 22:3 | [M+NH4]+ | C49H82O2 | 301.6 |
| 3.25 | 718.64852 | CE 22:4 | [M+NH4]+ | C49H80O2 | 298.8 |
| 3.44 | 752.72688 | CE 24:1 | [M+NH4]+, [M+Na]+ | C51H90O2 | 307.2 |
| 2.05 | 540.53492 | Cer 34:0;2O | [M+H]+ | C34H69NO3 | 259.3 |
| 1.97 | 538.51931 | Cer 34:1;2O | [M+H]+, [M+H-H2O]+ | C34H67NO3 | 257 |
| 1.75 | 536.50385 | Cer 34:2;2O | [M+H]+ | C34H65NO3 | 252.1 |
| 2.21 | 566.55228 | Cer 36:1;2O | [M+H]+, [M+H-H2O]+ | C36H71NO3 | 263.6 |
| 2.56 | 608.59837 | Cer 39:1;2O | [M+H]+ | C39H77NO3 | 272.7 |
| 2.73 | 624.62919 | Cer 40:0;2O | [M+H]+ | C40H81NO3 | 278.7 |
| 2.67 | 622.613 | Cer 40:1;2O | [M+H]+ | C40H79NO3 | 276.3 |
| 2.44 | 620.59848 | Cer 40:2;2O | [M+H]+ | C40H77NO3 | 272.8 |
| 2.75 | 636.62841 | Cer 41:1;2O | [M+H]+ | C41H81NO3 | 279.7 |
| 2.83 | 650.64465 | Cer 42:1;2O | [M+H]+ | C42H83NO3 | 282.6 |
| 2.66 | 648.62916 | Cer 42:2;2O | [M+H]+ | C42H81NO3 | 279.7 |
| 2.47 | 646.61212 | Cer 42:3;2O | [M+H]+ | C42H79NO3 | 275.9 |
| 2.89 | 664.65898 | Cer 43:1;2O | [M+H]+ | C43H85NO3 | 285.4 |
| 2.94 | 678.67504 | Cer 44:1;2O | [M+H]+ | C44H87NO3 | 288.5 |
| 2.81 | 676.65951 | Cer 44:2;2O | [M+H]+ | C44H85NO3 | 286.9 |
| 2.67 | 674.6428 | Cer 44:3;2O | [M+H]+ | C44H83NO3 | 282.6 |
| 2.16 | 558.50925 | DG 30:0 | [M+NH4]+, [M+H-H2O]+, [M+Na]+ | C33H64O5 | 254.4 |
| 2.39 | 586.53982 | DG 32:0 | [M+NH4]+, [M+Na]+, [M+H]+, [M+H-H2O]+, [M+K]+ | C35H68O5 | 260.7 |
| 2.2 | 584.52452 | DG 32:1 | [M+NH4]+, [M+K]+, [M+Na]+, [M+H]+, [M+H-H2O]+ | C35H66O5 | 257.2 |
| 2.01 | 582.50876 | DG 32:2 | [M+NH4]+ | C35H64O5 | 254.2 |
| 2.3 | 598.54065 | DG 33:1 | [M+NH4]+, [M+K]+, [M+Na]+, [M+H]+ | C36H68O5 | 261.3 |
| 2.62 | 614.57118 | DG 34:0 | [M+NH4]+, [M+K]+, [M+H]+, [M+H-H2O]+ | C37H72O5 | 267.6 |
| 2.41 | 612.55555 | DG 34:1 | [M+NH4]+, [M+H]+, [M+K]+, [M+Na]+, [M+H-H2O]+ | C37H70O5 | 265 |
| 2.23 | 610.53978 | DG 34:2 | [M+NH4]+, [M+H]+, [M+K]+, [M+Na]+ | C37H68O5 | 259.8 |
| 2.52 | 626.57107 | DG 35:1 | [M+NH4]+, [M+H]+, [M+Na]+, [M+K]+ | C38H72O5 | 268 |
| 2.33 | 624.55606 | DG 35:2 | [M+NH4]+, [M+K]+ | C38H70O5 | 264 |
| 2.63 | 640.58748 | DG 36:1 | [M+NH4]+, [M+K]+, [M+H]+, [M+Na]+ | C39H74O5 | 271 |
| 2.44 | 638.57119 | DG 36:2 | [M+NH4]+, [M+Na]+, [M+K]+, [M+H]+ | C39H72O5 | 267.5 |
| 2.26 | 636.55557 | DG 36:3 | [M+NH4]+, [M+Na]+, [M+K]+ | C39H70O5 | 264.1 |
| 2.18 | 634.54047 | DG 36:4 | [M+NH4]+ | C39H68O5 | 265.5 |
| 2.54 | 652.58741 | DG 37:2 | [M+NH4]+, [M+Na]+, [M+K]+ | C40H74O5 | 270.1 |
| 2.8 | 668.6186 | DG 38:1 | [M+NH4]+, [M+K]+, [M+Na]+ | C41H78O5 | 277.8 |
| 2.66 | 666.60196 | DG 38:2 | [M+NH4]+ | C41H76O5 | 274.3 |
| 2.46 | 664.58567 | DG 38:3_A | [M+NH4]+ | C41H74O5 | 270.8 |
| 2.58 | 664.58661 | DG 38:3_B | [M+NH4]+, [M+Na]+, [M+K]+, [M+H]+ | C41H74O5 | 274.9 |
| 2.42 | 662.57106 | DG 38:4 | [M+NH4]+, [M+Na]+ | C41H72O5 | 272.4 |
| 2.2 | 660.55477 | DG 38:5 | [M+NH4]+, [M+K]+, [M+Na]+ | C41H70O5 | 266.9 |
| 2.11 | 658.53952 | DG 38:6 | [M+NH4]+ | C41H68O5 | 269.3 |
| 2.93 | 696.65003 | DG 40:1 | [M+NH4]+, [M+Na]+, [M+K]+ | C43H82O5 | 284.1 |
| 2.42 | 688.58625 | DG 40:5 | [M+NH4]+ | C43H74O5 | 276.9 |
| 2.34 | 686.56919 | DG 40:6_A | [M+NH4]+ | C43H72O5 | 275.7 |
| 2.21 | 686.57084 | DG 40:6_B | [M+NH4]+, [M+K]+ | C43H72O5 | 272.2 |
| 3.02 | 724.67998 | DG 42:1 | [M+NH4]+, [M+Na]+, [M+K]+ | C45H86O5 | 290.1 |
| 2.92 | 722.66555 | DG 42:2 | [M+NH4]+, [M+Na]+, [M+K]+ | C45H84O5 | 286.4 |
| 3.11 | 752.71164 | DG 44:1 | [M+NH4]+ | C47H90O5 | 295.6 |
| 3.02 | 750.69588 | DG 44:2 | [M+NH4]+, [M+Na]+ | C47H88O5 | 292.7 |
| 2.93 | 748.67988 | DG 44:3 | [M+NH4]+ | C47H86O5 | 291.4 |
| 2.32 | 644.56019 | DG O-38:6 | [M+NH4]+, [M+Na]+, [M+H-H2O]+, [M+H]+, [M+K]+ | C41H70O4 | 268.3 |
| 2.62 | 674.60596 | DG O-40:5 | [M+NH4]+, [M+Na]+ | C43H76O4 | 276.4 |
| 2.47 | 672.59138 | DG O-40:6_A | [M+NH4]+, [M+Na]+ | C43H74O4 | 274.9 |
| 2.55 | 672.59156 | DG O-40:6_B | [M+NH4]+, [M+Na]+, [M+H]+ | C43H74O4 | 275.1 |
| 2.32 | 670.57525 | DG O-40:7 | [M+NH4]+, [M+K]+, [M+Na]+ | C43H72O4 | 270 |
| 0.89 | 537.48964 | FAHFA 34:0 | [M-H]- | C34H66O4 | 246.8 |
| 0.92 | 563.50513 | FAHFA 36:1 | [M-H]- | C36H68O4 | 250.6 |
| 1.29 | 1151.70507 | GM3 34:1;2O | [M-H]- | C57H104N2O21 | 338.1 |
| 2.11 | 1263.82913 | GM3 42:1;2O | [M-H]- | C65H120N2O21 | 349.5 |
| 1.41 | 834.59515 | Hex2Cer 32:1;2O | [M+H]+ | C44H83NO13 | 298.9 |
| 1.64 | 862.62449 | Hex2Cer 34:1;2O | [M+H]+ | C46H87NO13 | 304.5 |
| 2.34 | 946.71835 | Hex2Cer 40:1;2O | [M+H]+, [M+Na]+ | C52H99NO13 | 319.6 |
| 2.45 | 960.73444 | Hex2Cer 41:1;2O | [M+H]+, [M+Na]+ | C53H101NO13 | 322.3 |
| 2.56 | 974.74962 | Hex2Cer 42:1;2O | [M+H]+, [M+Na]+, [M+K]+, [M+H-H2O]+ | C54H103NO13 | 324.6 |
| 2.65 | 988.76523 | Hex2Cer 43:1;2O | [M+H]+ | C55H105NO13 | 327.1 |
| 2.74 | 1002.78148 | Hex2Cer 44:1;2O | [M+H]+ | C56H107NO13 | 329.8 |
| 1.58 | 1024.6782 | Hex3Cer 34:1;2O | [M+H]+, [M+K]+, [M+Na]+ | C52H97NO18 | 322.7 |
| 2.28 | 1108.7725 | Hex3Cer 40:1;2O | [M+H]+, [M+Na]+ | C58H109NO18 | 337.8 |
| 2.49 | 1136.8032 | Hex3Cer 42:1;2O | [M+H]+, [M+Na]+, [M+K]+ | C60H113NO18 | 342.4 |
| 2.69 | 1164.83527 | Hex3Cer 44:1;2O | [M+H]+ | C62H117NO18 | 346.9 |
| 1.73 | 700.57207 | HexCer 34:1;2O | [M+H]+ | C40H77NO8 | 281.8 |
| 2.44 | 784.6651 | HexCer 40:1;2O | [M+H]+, [M+Na]+ | C46H89NO8 | 298.8 |
| 2.65 | 812.69651 | HexCer 42:1;2O_A | [M+H]+, [M+Na]+, [M+H-H2O]+ | C48H93NO8 | 304.2 |
| 2.51 | 812.69731 | HexCer 42:1;2O_B | [M+H]+ | C48H93NO8 | 305.8 |
| 0.38 | 468.3089 | LPC 14:0 | [M+H]+ | C22H46NO7P | 225.3 |
| 0.43 | 482.32481 | LPC 15:0 | [M+H]+ | C23H48NO7P | 228.8 |
| 0.49 | 496.33998 | LPC 16:0 | [M+H]+, [M+Na]+, [M+K]+ | C24H50NO7P | 232.1 |
| 0.39 | 494.32415 | LPC 16:1 | [M+H]+, [M+Na]+ | C24H48NO7P | 228.7 |
| 0.56 | 510.3554 | LPC 17:0 | [M+H]+ | C25H52NO7P | 235.6 |
| 0.44 | 508.34029 | LPC 17:1 | [M+H]+ | C25H50NO7P | 232 |
| 0.66 | 524.3712 | LPC 18:0 | [M+H]+, [M+Na]+ | C26H54NO7P | 239.9 |
| 0.48 | 580.36251 | LPC 18:1_A | [M+CH3COO]- | C26H52NO7P | 246.1 |
| 0.5 | 522.35561 | LPC 18:1_B | [M+H]+, [M+Na]+, [M+K]+ | C26H52NO7P | 235.9 |
| 0.42 | 520.33916 | LPC 18:2 | [M+H]+ | C26H50NO7P | 230.9 |
| 0.58 | 536.37209 | LPC 19:1 | [M+H]+ | C27H54NO7P | 237.9 |
| 0.68 | 550.38673 | LPC 20:1 | [M+H]+ | C28H56NO7P | 242.1 |
| 0.54 | 548.36985 | LPC 20:2 | [M+H]+ | C28H54NO7P | 238.1 |
| 0.4 | 544.33975 | LPC 20:4 | [M+H]+ | C28H50NO7P | 233.4 |
| 1.16 | 580.43329 | LPC 22:0 | [M+H]+ | C30H62NO7P | 254 |
| 0.58 | 574.38738 | LPC 22:3 | [M+H]+ | C30H56NO7P | 242.4 |
| 0.39 | 568.3395 | LPC 22:6 | [M+H]+ | C30H50NO7P | 235.5 |
| 1.42 | 608.46552 | LPC 24:0 | [M+H]+, [M+Na]+ | C32H66NO7P | 260.5 |
| 1.18 | 606.45018 | LPC 24:1 | [M+H]+ | C32H64NO7P | 255.7 |
| 1.2 | 632.46699 | LPC 26:2 | [M+H]+ | C34H66NO7P | 259.2 |
| 0.57 | 482.36032 | LPC O-16:0 | [M+H]+ | C24H52NO6P | 233 |
| 0.54 | 480.34501 | LPC O-16:1 | [M+H]+ | C24H50NO6P | 228 |
| 0.78 | 510.39171 | LPC O-18:0 | [M+H]+ | C26H56NO6P | 240.8 |
| 0.59 | 508.37627 | LPC O-18:1 | [M+H]+ | C26H54NO6P | 235.1 |
| 0.5 | 454.29265 | LPE 16:0 | [M+H]+ | C21H44NO7P | 216.1 |
| 0.67 | 482.32455 | LPE 18:0 | [M+H]+ | C23H48NO7P | 224 |
| 0.52 | 480.30881 | LPE 18:1_A | [M+H]+, [M+Na]+ | C23H46NO7P | 217.7 |
| 0.49 | 478.29447 | LPE 18:1_B | [M-H]- | C23H46NO7P | 215.4 |
| 0.37 | 502.29443 | LPE 20:4 | [M+H]+ | C25H44NO7P | 218.1 |
| 0.39 | 526.29254 | LPE 22:6 | [M+H]+ | C27H44NO7P | 221 |
| 1.43 | 566.41788 | LPE 24:0 | [M+H]+ | C29H60NO7P | 245.2 |
| 1.19 | 564.40336 | LPE 24:1 | [M+H]+ | C29H58NO7P | 240.6 |
| 1.43 | 592.43311 | LPE 26:1 | [M+H]+, [M+Na]+ | C31H62NO7P | 247.3 |
| 0.56 | 438.29876 | LPE O-16:1_A | [M+H]+, [M+Na]+ | C21H44NO6P | 214.3 |
| 0.54 | 436.28389 | LPE O-16:1_B | [M-H]- | C21H44NO6P | 209.6 |
| 0.76 | 466.32952 | LPE O-18:1_A | [M+H]+ | C23H48NO6P | 222.6 |
| 0.73 | 464.31535 | LPE O-18:1_B | [M-H]- | C23H48NO6P | 216.7 |
| 0.58 | 464.31469 | LPE O-18:2_A | [M+H]+, [M+Na]+ | C23H46NO6P | 216.4 |
| 0.55 | 462.29938 | LPE O-18:2_B | [M-H]- | C23H46NO6P | 214.1 |
| 1.06 | 494.36079 | LPE O-20:1 | [M+H]+ | C25H52NO6P | 230.7 |
| 2.31 | 758.57122 | LPE-N (FA) 37:1 | [M-H]- | C42H82NO8P | 278.7 |
| 2.44 | 822.60265 | LPE-N (FA) 42:4 | [M-H]- | C47H86NO8P | 289.7 |
| 1.99 | 792.55728 | LPE-N (FA) 50:5 | [M-H]- | C45H80NO8P | 283.1 |
| 0.49 | 524.33482 | PC 17:0 | [M+H]+ | C25H50NO8P | 238.6 |
| 0.53 | 538.35077 | PC 18:0 | [M+H]+ | C26H52NO8P | 238.8 |
| 1.23 | 650.47512 | PC 26:0 | [M+H]+ | C34H68NO8P | 268.9 |
| 1.33 | 664.49199 | PC 27:0 | [M+H]+ | C35H70NO8P | 271.9 |
| 1.44 | 678.50724 | PC 28:0 | [M+H]+ | C36H72NO8P | 274.8 |
| 1.27 | 676.49077 | PC 28:1 | [M+H]+ | C36H70NO8P | 271.8 |
| 1.55 | 692.52231 | PC 29:0 | [M+H]+, [M+Na]+ | C37H74NO8P | 277.4 |
| 1.4 | 690.50754 | PC 29:1 | [M+H]+ | C37H72NO8P | 275.6 |
| 1.64 | 764.54483 | PC 30:0_A | [M+CH3COO]- | C38H76NO8P | 285.3 |
| 1.67 | 706.53851 | PC 30:0_B | [M+H]+ | C38H76NO8P | 280.7 |
| 1.46 | 762.5288 | PC 30:1_A | [M+CH3COO]- | C38H74NO8P | 283.7 |
| 1.48 | 704.52285 | PC 30:1_B | [M+H]+ | C38H74NO8P | 277.9 |
| 1.4 | 702.50633 | PC 30:2 | [M+H]+, [M+K]+, [M+Na]+ | C38H72NO8P | 276.7 |
| 1.26 | 700.49405 | PC 30:3 | [M+H]+ | C38H70NO8P | 274.6 |
| 1.79 | 720.55339 | PC 31:0 | [M+H]+ | C39H78NO8P | 283.5 |
| 1.59 | 718.53828 | PC 31:1 | [M+H]+, [M+Na]+ | C39H76NO8P | 280.5 |
| 1.46 | 716.52348 | PC 31:2 | [M+H]+ | C39H74NO8P | 279.9 |
| 1.88 | 792.57625 | PC 32:0_A | [M+CH3COO]- | C40H80NO8P | 290.8 |
| 1.89 | 734.56975 | PC 32:0_B | [M+H]+, [M+Na]+ | C40H80NO8P | 286.6 |
| 1.7 | 732.55424 | PC 32:1 | [M+H]+ | C40H78NO8P | 284 |
| 1.5 | 788.5442 | PC 32:2_A | [M+CH3COO]- | C40H76NO8P | 288.2 |
| 1.53 | 730.53861 | PC 32:2_B | [M+H]+ | C40H76NO8P | 281.4 |
| 1.44 | 728.52153 | PC 32:3 | [M+H]+ | C40H74NO8P | 280.2 |
| 1.31 | 726.50715 | PC 32:4 | [M+H]+ | C40H72NO8P | 277.9 |
| 1.79 | 804.57412 | PC 33:1_A | [M+CH3COO]- | C41H80NO8P | 292.1 |
| 1.82 | 746.56896 | PC 33:1_B | [M+H]+ | C41H80NO8P | 286.8 |
| 1.62 | 744.55384 | PC 33:2 | [M+H]+ | C41H78NO8P | 283.3 |
| 1.51 | 742.53766 | PC 33:3 | [M+H]+ | C41H76NO8P | 283.5 |
| 2.12 | 820.60685 | PC 34:0_A | [M+CH3COO]- | C42H84NO8P | 295.9 |
| 2.14 | 762.60036 | PC 34:0_B | [M+H]+, [M+K]+ | C42H84NO8P | 292.9 |
| 1.91 | 818.59254 | PC 34:1_A | [M+CH3COO]- | C42H82NO8P | 295.6 |
| 1.93 | 760.58518 | PC 34:1_B | [M+H]+ | C42H82NO8P | 290.4 |
| 1.73 | 816.57625 | PC 34:2_A | [M+CH3COO]- | C42H80NO8P | 293.9 |
| 1.73 | 758.56977 | PC 34:2_B | [M+H]+ | C42H80NO8P | 287.5 |
| 1.54 | 814.55981 | PC 34:3_A | [M+CH3COO]- | C42H78NO8P | 292.3 |
| 1.58 | 756.55314 | PC 34:3_B | [M+H]+ | C42H78NO8P | 284.9 |
| 1.49 | 754.53697 | PC 34:4 | [M+H]+, [M+Na]+ | C42H76NO8P | 284.2 |
| 1.35 | 752.52133 | PC 34:5 | [M+H]+, [M+Na]+ | C42H74NO8P | 281.3 |
| 1.22 | 750.50668 | PC 34:6 | [M+H]+ | C42H72NO8P | 279.3 |
| 2.22 | 776.6151 | PC 35:0 | [M+H]+ | C43H86NO8P | 295.5 |
| 2.04 | 774.59977 | PC 35:1 | [M+H]+ | C43H84NO8P | 293.4 |
| 1.85 | 772.58511 | PC 35:2 | [M+H]+ | C43H82NO8P | 290.4 |
| 1.68 | 770.56846 | PC 35:3 | [M+H]+ | C43H80NO8P | 288.7 |
| 1.59 | 768.55228 | PC 35:4 | [M+H]+ | C43H78NO8P | 286.8 |
| 1.45 | 766.54121 | PC 35:5 | [M+H]+ | C43H76NO8P | 285.1 |
| 2.38 | 790.62885 | PC 36:0 | [M+H]+ | C44H88NO8P | 298.2 |
| 2.17 | 788.6165 | PC 36:1_A | [M+H]+ | C44H86NO8P | 295.7 |
| 2.14 | 846.62334 | PC 36:1_B | [M+CH3COO]- | C44H86NO8P | 300.5 |
| 1.93 | 844.60769 | PC 36:2_A | [M+CH3COO]- | C44H84NO8P | 299.9 |
| 1.96 | 786.6009 | PC 36:2_B | [M+H]+ | C44H84NO8P | 293.8 |
| 1.76 | 842.59056 | PC 36:3_A | [M+CH3COO]- | C44H82NO8P | 297.8 |
| 1.79 | 784.58441 | PC 36:3_B | [M+H]+ | C44H82NO8P | 291.3 |
| 1.61 | 782.56808 | PC 36:4_A | [M+H]+ | C44H80NO8P | 289.4 |
| 1.71 | 782.56771 | PC 36:4_B | [M+H]+, [M+Na]+ | C44H80NO8P | 290.3 |
| 1.69 | 840.57571 | PC 36:4_C | [M+CH3COO]- | C44H80NO8P | 297.8 |
| 1.52 | 838.55809 | PC 36:5_A | [M+CH3COO]- | C44H78NO8P | 295.3 |
| 1.55 | 780.55285 | PC 36:5_B | [M+H]+ | C44H78NO8P | 287.5 |
| 1.39 | 836.54473 | PC 36:6_A | [M+CH3COO]- | C44H76NO8P | 293.6 |
| 1.43 | 778.53739 | PC 36:6_B | [M+H]+, [M+Na]+ | C44H76NO8P | 286.3 |
| 1.3 | 776.52477 | PC 36:7 | [M+H]+ | C44H74NO8P | 283.7 |
| 2.27 | 802.63146 | PC 37:1 | [M+H]+ | C45H88NO8P | 298.9 |
| 2.06 | 800.61631 | PC 37:2 | [M+H]+ | C45H86NO8P | 296.5 |
| 1.63 | 794.5683 | PC 37:5 | [M+H]+ | C45H80NO8P | 291 |
| 1.53 | 792.55285 | PC 37:6 | [M+H]+ | C45H78NO8P | 289.1 |
| 2.6 | 818.66152 | PC 38:0 | [M+H]+ | C46H92NO8P | 303.5 |
| 2.38 | 816.64678 | PC 38:1 | [M+H]+, [M+Na]+, [M+K]+ | C46H90NO8P | 300.6 |
| 2.15 | 872.63859 | PC 38:2_A | [M+CH3COO]- | C46H88NO8P | 305.2 |
| 2.18 | 814.63161 | PC 38:2_B | [M+H]+, [M+Na]+, [M+K]+ | C46H88NO8P | 299.5 |
| 1.98 | 870.622 | PC 38:3_A | [M+CH3COO]- | C46H86NO8P | 303.1 |
| 2 | 812.61541 | PC 38:3_B | [M+H]+ | C46H86NO8P | 297.2 |
| 1.82 | 868.60711 | PC 38:4_A | [M+CH3COO]- | C46H84NO8P | 302.4 |
| 1.85 | 810.59991 | PC 38:4_B | [M+H]+, [M+Na]+ | C46H84NO8P | 294.9 |
| 1.73 | 808.58414 | PC 38:5_A | [M+H]+, [M+Na]+ | C46H82NO8P | 293.3 |
| 1.71 | 866.59074 | PC 38:5_B | [M+CH3COO]- | C46H82NO8P | 301.7 |
| 1.65 | 806.56902 | PC 38:6 | [M+H]+ | C46H80NO8P | 292.3 |
| 1.46 | 804.55295 | PC 38:7_A | [M+H]+ | C46H78NO8P | 289.6 |
| 1.44 | 862.55719 | PC 38:7_B | [M+CH3COO]- | C46H78NO8P | 298.6 |
| 2.49 | 830.66247 | PC 39:1 | [M+H]+ | C47H92NO8P | 303.9 |
| 2.3 | 828.64708 | PC 39:2 | [M+H]+ | C47H90NO8P | 302.3 |
| 2.11 | 826.63158 | PC 39:3 | [M+H]+ | C47H88NO8P | 300.2 |
| 1.72 | 820.58368 | PC 39:6 | [M+H]+ | C47H82NO8P | 294.9 |
| 1.56 | 818.57136 | PC 39:7 | [M+H]+ | C47H80NO8P | 292.8 |
| 2.78 | 846.69405 | PC 40:0 | [M+H]+ | C48H96NO8P | 309.8 |
| 2.59 | 844.6779 | PC 40:1 | [M+H]+, [M+K]+, [M+Na]+ | C48H94NO8P | 306.2 |
| 2.39 | 842.66255 | PC 40:2 | [M+H]+, [M+K]+, [M+Na]+ | C48H92NO8P | 304.4 |
| 2.23 | 840.64728 | PC 40:3 | [M+H]+, [M+Na]+ | C48H90NO8P | 303.3 |
| 2.07 | 838.63088 | PC 40:4 | [M+H]+ | C48H88NO8P | 300.9 |
| 1.75 | 834.60135 | PC 40:6 | [M+H]+ | C48H84NO8P | 296.3 |
| 1.64 | 890.59099 | PC 40:7_A | [M+CH3COO]- | C48H82NO8P | 303.8 |
| 1.67 | 832.58438 | PC 40:7_B | [M+H]+ | C48H82NO8P | 295.5 |
| 1.52 | 830.56836 | PC 40:8 | [M+H]+, [M+K]+ | C48H80NO8P | 293.1 |
| 1.39 | 828.55104 | PC 40:9 | [M+H]+ | C48H78NO8P | 292.1 |
| 2.85 | 860.71417 | PC 41:0 | [M+H]+ | C49H98NO8P | 312.2 |
| 2.69 | 858.69404 | PC 41:1 | [M+H]+ | C49H96NO8P | 310 |
| 2.51 | 856.68037 | PC 41:2 | [M+H]+ | C49H94NO8P | 308.4 |
| 2.32 | 854.66329 | PC 41:3 | [M+H]+ | C49H92NO8P | 305.7 |
| 1.78 | 846.60049 | PC 41:7 | [M+H]+ | C49H84NO8P | 298.6 |
| 2.91 | 874.72504 | PC 42:0 | [M+H]+ | C50H100NO8P | 315.3 |
| 2.78 | 872.70987 | PC 42:1 | [M+H]+, [M+Na]+, [M+K]+ | C50H98NO8P | 312.8 |
| 1.46 | 854.56836 | PC 42:10 | [M+H]+, [M+K]+ | C50H80NO8P | 295.7 |
| 1.32 | 852.552 | PC 42:11 | [M+H]+ | C50H78NO8P | 294.1 |
| 2.6 | 870.6936 | PC 42:2 | [M+H]+, [M+Na]+, [M+K]+ | C50H96NO8P | 311.3 |
| 2.43 | 868.67826 | PC 42:3 | [M+H]+ | C50H94NO8P | 309.3 |
| 2.27 | 866.66249 | PC 42:4 | [M+H]+, [M+Na]+ | C50H92NO8P | 307.2 |
| 2.13 | 864.64643 | PC 42:5 | [M+H]+, [M+Na]+ | C50H90NO8P | 305.1 |
| 1.99 | 862.63168 | PC 42:6 | [M+H]+ | C50H88NO8P | 303.3 |
| 1.82 | 860.61543 | PC 42:7 | [M+H]+ | C50H86NO8P | 301.1 |
| 1.7 | 858.59932 | PC 42:8 | [M+H]+ | C50H84NO8P | 299 |
| 2.85 | 886.72565 | PC 43:1 | [M+H]+ | C51H100NO8P | 315.4 |
| 2.7 | 884.70974 | PC 43:2 | [M+H]+ | C51H98NO8P | 313.3 |
| 2.52 | 882.69501 | PC 43:3 | [M+H]+ | C51H96NO8P | 311.7 |
| 3.02 | 902.75919 | PC 44:0 | [M+H]+ | C52H104NO8P | 321 |
| 2.91 | 900.74056 | PC 44:1 | [M+H]+ | C52H102NO8P | 318.3 |
| 1.48 | 880.58238 | PC 44:11 | [M+H]+ | C52H82NO8P | 298.9 |
| 1.41 | 878.56817 | PC 44:12 | [M+H]+ | C52H80NO8P | 298.5 |
| 2.78 | 898.72572 | PC 44:2 | [M+H]+, [M+K]+, [M+Na]+ | C52H100NO8P | 316.5 |
| 2.62 | 896.70946 | PC 44:3 | [M+H]+ | C52H98NO8P | 314.6 |
| 2.47 | 894.6936 | PC 44:4 | [M+H]+, [M+Na]+ | C52H96NO8P | 312.9 |
| 2.33 | 892.67795 | PC 44:5 | [M+H]+, [M+Na]+ | C52H94NO8P | 310.8 |
| 2.14 | 890.66257 | PC 44:6 | [M+H]+ | C52H92NO8P | 308.7 |
| 1.99 | 888.64729 | PC 44:7 | [M+H]+ | C52H90NO8P | 306.6 |
| 1.86 | 886.63125 | PC 44:8 | [M+H]+ | C52H88NO8P | 304.7 |
| 2.96 | 914.76057 | PC 45:1 | [M+H]+ | C53H104NO8P | 321.2 |
| 2.85 | 912.74207 | PC 45:2 | [M+H]+ | C53H102NO8P | 318.4 |
| 3.02 | 928.7718 | PC 46:1 | [M+H]+ | C54H106NO8P | 323.7 |
| 2.91 | 926.75714 | PC 46:2 | [M+H]+ | C54H104NO8P | 321.5 |
| 2.79 | 924.73868 | PC 46:3 | [M+H]+ | C54H102NO8P | 320.1 |
| 2.61 | 920.70881 | PC 46:5 | [M+H]+, [M+Na]+ | C54H98NO8P | 316.3 |
| 2.6 | 918.69776 | PC 46:6_A | [M+H]+ | C54H96NO8P | 316.2 |
| 2.38 | 918.69336 | PC 46:6_B | [M+H]+ | C54H96NO8P | 314.4 |
| 2.19 | 916.67828 | PC 46:7 | [M+H]+ | C54H94NO8P | 312 |
| 2.09 | 914.66294 | PC 46:8 | [M+H]+ | C54H92NO8P | 311 |
| 2.92 | 952.76946 | PC 48:3 | [M+H]+ | C56H106NO8P | 324.6 |
| 2.86 | 950.75336 | PC 48:4 | [M+H]+ | C56H104NO8P | 323 |
| 2.78 | 948.73813 | PC 48:5 | [M+H]+ | C56H102NO8P | 322.1 |
| 2.39 | 944.70875 | PC 48:7_A | [M+H]+ | C56H98NO8P | 318.2 |
| 2.55 | 944.70969 | PC 48:7_B | [M+H]+ | C56H98NO8P | 318.5 |
| 2.34 | 942.69355 | PC 48:8 | [M+H]+ | C56H96NO8P | 316.8 |
| 2.72 | 974.75723 | PC 50:6 | [M+H]+ | C58H104NO8P | 325.7 |
| 2.44 | 970.72616 | PC 50:8 | [M+H]+ | C58H100NO8P | 322.4 |
| 2.56 | 1048.77364 | PC 56:11 | [M+H]+ | C64H106NO8P | 334.6 |
| 1.36 | 636.49669 | PC O-26:0 | [M+H]+ | C34H70NO7P | 272.1 |
| 1.41 | 634.48054 | PC O-26:1 | [M+H]+, [M+Na]+ | C34H68NO7P | 262.2 |
| 1.55 | 662.51103 | PC O-28:1 | [M+H]+ | C36H72NO7P | 273.3 |
| 1.44 | 660.49819 | PC O-28:2 | [M+H]+ | C36H70NO7P | 265.6 |
| 1.59 | 688.52761 | PC O-30:2 | [M+H]+ | C38H74NO7P | 276.5 |
| 2.06 | 720.59009 | PC O-32:0 | [M+H]+, [M+K]+, [M+Na]+ | C40H82NO7P | 288.4 |
| 1.86 | 718.57397 | PC O-32:1 | [M+H]+, [M+Na]+ | C40H80NO7P | 285.6 |
| 1.27 | 710.51123 | PC O-32:5 | [M+H]+ | C40H72NO7P | 277.3 |
| 2.16 | 734.60432 | PC O-33:0 | [M+H]+ | C41H84NO7P | 290.6 |
| 1.97 | 732.58915 | PC O-33:1 | [M+H]+ | C41H82NO7P | 287.8 |
| 2.3 | 748.62112 | PC O-34:0 | [M+H]+ | C42H86NO7P | 294.5 |
| 2.23 | 804.61259 | PC O-34:1_A | [M+CH3COO]- | C42H84NO7P | 295.7 |
| 2.07 | 746.6059 | PC O-34:1_B | [M+H]+ | C42H84NO7P | 291.7 |
| 1.88 | 744.58949 | PC O-34:2_A | [M+H]+ | C42H82NO7P | 289.7 |
| 2.04 | 744.58962 | PC O-34:2_B | [M+H]+ | C42H82NO7P | 289.1 |
| 2.37 | 762.63547 | PC O-35:0 | [M+H]+ | C43H88NO7P | 296.6 |
| 2.2 | 760.62067 | PC O-35:1 | [M+H]+ | C43H86NO7P | 293.8 |
| 2.15 | 758.60596 | PC O-35:2 | [M+H]+ | C43H84NO7P | 291.1 |
| 2.54 | 776.65148 | PC O-36:0 | [M+H]+ | C44H90NO7P | 300 |
| 2.32 | 774.63656 | PC O-36:1 | [M+H]+ | C44H88NO7P | 298 |
| 2.28 | 772.62068 | PC O-36:2 | [M+H]+, [M+Na]+ | C44H86NO7P | 295.7 |
| 1.7 | 766.57148 | PC O-36:5 | [M+H]+ | C44H80NO7P | 289.6 |
| 1.66 | 764.55819 | PC O-36:6 | [M+H]+ | C44H78NO7P | 286.8 |
| 1.91 | 762.54105 | PC O-36:7 | [M+H]+ | C44H76NO7P | 281.6 |
| 2.64 | 846.65786 | PC O-37:1 | [M+CH3COO]- | C45H90NO7P | 300.7 |
| 1.88 | 780.58854 | PC O-37:5 | [M+H]+ | C45H82NO7P | 292.4 |
| 2.26 | 776.5572 | PC O-37:7 | [M+H]+ | C45H78NO7P | 290.4 |
| 2.74 | 804.68346 | PC O-38:0 | [M+H]+ | C46H94NO7P | 305.8 |
| 2.55 | 802.66809 | PC O-38:1 | [M+H]+ | C46H92NO7P | 303.4 |
| 2.5 | 800.65185 | PC O-38:2_A | [M+H]+ | C46H90NO7P | 300.7 |
| 2.35 | 800.65206 | PC O-38:2_B | [M+H]+ | C46H90NO7P | 300.7 |
| 2.2 | 798.63575 | PC O-38:3 | [M+H]+ | C46H88NO7P | 298.9 |
| 2.08 | 796.62038 | PC O-38:4 | [M+H]+ | C46H86NO7P | 297.2 |
| 1.79 | 792.58898 | PC O-38:6 | [M+H]+ | C46H82NO7P | 294.3 |
| 1.88 | 806.60721 | PC O-39:6 | [M+H]+ | C47H84NO7P | 296.9 |
| 2.46 | 804.58615 | PC O-39:7 | [M+H]+ | C47H82NO7P | 296.9 |
| 2.88 | 832.71459 | PC O-40:0 | [M+H]+ | C48H98NO7P | 311.2 |
| 2.69 | 888.71158 | PC O-40:1_A | [M+CH3COO]- | C48H96NO7P | 309.9 |
| 2.73 | 830.69936 | PC O-40:1_B | [M+H]+ | C48H96NO7P | 309 |
| 2.55 | 828.68395 | PC O-40:2 | [M+H]+ | C48H94NO7P | 307 |
| 2.39 | 826.66711 | PC O-40:3 | [M+H]+ | C48H92NO7P | 303.5 |
| 2.22 | 824.65256 | PC O-40:4 | [M+H]+, [M+Na]+ | C48H90NO7P | 302.5 |
| 2.07 | 880.64266 | PC O-40:5 | [M+CH3COO]- | C48H88NO7P | 305.1 |
| 2 | 878.62805 | PC O-40:6_A | [M+CH3COO]- | C48H86NO7P | 304.4 |
| 2.02 | 820.62078 | PC O-40:6_B | [M+H]+ | C48H86NO7P | 300.2 |
| 1.78 | 876.61124 | PC O-40:7 | [M+CH3COO]- | C48H84NO7P | 303.4 |
| 1.77 | 816.58844 | PC O-40:8 | [M+H]+ | C48H82NO7P | 295.3 |
| 2.88 | 858.73008 | PC O-42:1 | [M+H]+ | C50H100NO7P | 314.2 |
| 2.74 | 856.71478 | PC O-42:2 | [M+H]+ | C50H98NO7P | 312.4 |
| 2.53 | 852.68308 | PC O-42:4 | [M+H]+ | C50H94NO7P | 309.4 |
| 2.32 | 850.66634 | PC O-42:5 | [M+H]+ | C50H92NO7P | 306.9 |
| 2.16 | 848.65239 | PC O-42:6 | [M+H]+ | C50H90NO7P | 305.2 |
| 2.55 | 878.69644 | PC O-44:5 | [M+H]+ | C52H96NO7P | 312.6 |
| 2.47 | 876.68207 | PC O-44:6 | [M+H]+ | C52H94NO7P | 311.7 |
| 2.63 | 904.71562 | PC O-46:6 | [M+H]+ | C54H98NO7P | 316.7 |
| 2.98 | 924.77911 | PC O-47:3 | [M+H]+ | C55H106NO7P | 319.6 |
| 1.7 | 662.4798 | PE 30:0 | [M-H]- | C35H70NO8P | 257.8 |
| 1.53 | 662.4764 | PE 30:1 | [M+H]+ | C35H68NO8P | 264.8 |
| 1.97 | 692.52212 | PE 32:0_A | [M+H]+ | C37H74NO8P | 275.1 |
| 1.94 | 690.50863 | PE 32:0_B | [M-H]- | C37H74NO8P | 266.2 |
| 1.76 | 690.50731 | PE 32:1 | [M+H]+ | C37H72NO8P | 270.8 |
| 1.57 | 688.49126 | PE 32:2_A | [M+H]+ | C37H70NO8P | 268.1 |
| 1.54 | 686.47882 | PE 32:2_B | [M-H]- | C37H70NO8P | 260 |
| 1.88 | 704.52236 | PE 33:1_A | [M+H]+ | C38H74NO8P | 273.7 |
| 1.85 | 702.50932 | PE 33:1_B | [M-H]- | C38H74NO8P | 264.6 |
| 2.21 | 720.55355 | PE 34:0_A | [M+H]+ | C39H78NO8P | 281.1 |
| 2.18 | 718.53925 | PE 34:0_B | [M-H]- | C39H78NO8P | 270.8 |
| 1.99 | 718.53714 | PE 34:1_A | [M+H]+, [M+Na]+ | C39H76NO8P | 276.9 |
| 1.97 | 716.52374 | PE 34:1_B | [M-H]- | C39H76NO8P | 269.4 |
| 1.76 | 714.50822 | PE 34:2_A | [M-H]- | C39H74NO8P | 266.5 |
| 1.79 | 716.52246 | PE 34:2_B | [M+H]+, [M+Na]+ | C39H74NO8P | 273.4 |
| 1.63 | 714.50659 | PE 34:3_A | [M+H]+ | C39H72NO8P | 271.2 |
| 1.69 | 712.49282 | PE 34:3_B | [M-H]- | C39H72NO8P | 265.5 |
| 1.59 | 712.49344 | PE 34:3_C | [M-H]- | C39H72NO8P | 264.8 |
| 1.54 | 712.49097 | PE 34:4 | [M+H]+ | C39H70NO8P | 270.9 |
| 1.42 | 710.47336 | PE 34:5 | [M+H]+ | C39H68NO8P | 268.7 |
| 2.08 | 730.53958 | PE 35:1_A | [M-H]- | C40H78NO8P | 272.2 |
| 2.12 | 732.55296 | PE 35:1_B | [M+H]+ | C40H78NO8P | 280.6 |
| 1.9 | 730.53806 | PE 35:2_A | [M+H]+ | C40H76NO8P | 277 |
| 1.88 | 728.52392 | PE 35:2_B | [M-H]- | C40H76NO8P | 268.9 |
| 2.22 | 746.56952 | PE 36:1_A | [M+H]+, [M+K]+, [M+Na]+ | C41H80NO8P | 283.5 |
| 2.2 | 744.55571 | PE 36:1_B | [M-H]- | C41H80NO8P | 275.9 |
| 1.81 | 740.52333 | PE 36:3 | [M-H]- | C41H76NO8P | 271 |
| 1.74 | 738.50825 | PE 36:4 | [M-H]- | C41H74NO8P | 270.9 |
| 1.58 | 738.50669 | PE 36:5_A | [M+H]+ | C41H72NO8P | 273.5 |
| 1.54 | 736.49535 | PE 36:5_B | [M-H]- | C41H72NO8P | 268.2 |
| 1.44 | 736.49186 | PE 36:6 | [M+H]+ | C41H70NO8P | 270.9 |
| 2.1 | 756.55538 | PE 37:2 | [M-H]-, [M-H-H2O]- | C42H80NO8P | 276.4 |
| 2.45 | 774.60049 | PE 38:1_A | [M+H]+, [M+Na]+ | C43H84NO8P | 289.8 |
| 2.42 | 772.58662 | PE 38:1_B | [M-H]- | C43H84NO8P | 281.7 |
| 2.21 | 770.57049 | PE 38:2_A | [M-H]-, [M-H-H2O]- | C43H82NO8P | 281.1 |
| 2.23 | 772.5849 | PE 38:2_B | [M+H]+, [M+H-H2O]+ | C43H82NO8P | 287.4 |
| 2.12 | 770.56962 | PE 38:3_A | [M+H]+ | C43H80NO8P | 285.6 |
| 2.1 | 768.55473 | PE 38:3_B | [M-H]- | C43H80NO8P | 279.3 |
| 1.89 | 768.55218 | PE 38:4_A | [M+H]+ | C43H78NO8P | 281.7 |
| 2.01 | 768.55347 | PE 38:4_B | [M+H]+ | C43H78NO8P | 284.5 |
| 1.99 | 766.53941 | PE 38:4_C | [M-H]- | C43H78NO8P | 278.8 |
| 1.79 | 766.5375 | PE 38:5_A | [M+H]+ | C43H76NO8P | 280.3 |
| 1.77 | 764.52395 | PE 38:5_B | [M-H]- | C43H76NO8P | 275.8 |
| 1.66 | 764.52351 | PE 38:6_A | [M+H]+ | C43H74NO8P | 277.8 |
| 1.69 | 762.50866 | PE 38:6_B | [M-H]- | C43H74NO8P | 275.7 |
| 1.51 | 762.50777 | PE 38:7_A | [M+H]+, [M+Na]+ | C43H72NO8P | 276 |
| 1.48 | 760.49664 | PE 38:7_B | [M-H]- | C43H72NO8P | 272.4 |
| 2.32 | 784.58674 | PE 39:2 | [M-H]- | C44H84NO8P | 282.6 |
| 2.21 | 782.57041 | PE 39:3 | [M-H]- | C44H82NO8P | 283.3 |
| 2.78 | 802.63434 | PE 40:0 | [M-H]- | C45H90NO8P | 288.9 |
| 2.66 | 802.63161 | PE 40:1_A | [M+H]+, [M+Na]+ | C45H88NO8P | 295.9 |
| 2.64 | 800.61794 | PE 40:1_B | [M-H]- | C45H88NO8P | 289.2 |
| 2.45 | 800.61619 | PE 40:2_A | [M+H]+, [M+K]+ | C45H86NO8P | 293.5 |
| 2.43 | 798.60169 | PE 40:2_B | [M-H]- | C45H86NO8P | 286.1 |
| 2.29 | 796.58633 | PE 40:3_A | [M-H]- | C45H84NO8P | 284.9 |
| 2.33 | 798.60288 | PE 40:3_B | [M+H]+ | C45H84NO8P | 292.6 |
| 2.17 | 796.58541 | PE 40:4_A | [M+H]+ | C45H82NO8P | 289.7 |
| 2.15 | 794.57078 | PE 40:4_B | [M-H]- | C45H82NO8P | 283.1 |
| 2.01 | 794.56926 | PE 40:5 | [M+H]+ | C45H80NO8P | 287.6 |
| 1.95 | 792.55696 | PE 40:6_A | [M+H]+ | C45H78NO8P | 285.8 |
| 1.92 | 790.54121 | PE 40:6_B | [M-H]- | C45H78NO8P | 281.2 |
| 1.73 | 790.53854 | PE 40:7_A | [M+H]+ | C45H76NO8P | 282.9 |
| 1.7 | 788.52476 | PE 40:7_B | [M-H]- | C45H76NO8P | 280 |
| 1.57 | 788.52284 | PE 40:8 | [M+H]+ | C45H74NO8P | 280.7 |
| 2.74 | 816.64586 | PE 41:1 | [M+H]+, [M+Na]+ | C46H90NO8P | 299 |
| 2.8 | 828.64855 | PE 42:1 | [M-H]- | C47H92NO8P | 294.9 |
| 2.63 | 826.63259 | PE 42:2_A | [M-H]- | C47H90NO8P | 293.2 |
| 2.65 | 828.6473 | PE 42:2_B | [M+H]+ | C47H90NO8P | 299.2 |
| 2.47 | 826.63181 | PE 42:3_A | [M+H]+ | C47H88NO8P | 297.6 |
| 2.45 | 824.6172 | PE 42:3_B | [M-H]- | C47H88NO8P | 290.8 |
| 2.33 | 824.61502 | PE 42:4 | [M+H]+ | C47H86NO8P | 296.2 |
| 2.09 | 820.58697 | PE 42:6_A | [M+H]+ | C47H82NO8P | 293.6 |
| 2.06 | 818.57411 | PE 42:6_B | [M-H]- | C47H82NO8P | 286.8 |
| 2.88 | 844.67697 | PE 43:1_A | [M+H]+, [M+Na]+ | C48H94NO8P | 304.6 |
| 2.86 | 842.66587 | PE 43:1_B | [M-H]- | C48H94NO8P | 296.2 |
| 2.73 | 842.66315 | PE 43:2_A | [M+H]+ | C48H92NO8P | 302 |
| 2.71 | 840.64839 | PE 43:2_B | [M-H]- | C48H92NO8P | 294.6 |
| 2.93 | 858.69381 | PE 44:1_A | [M+H]+, [M+Na]+ | C49H96NO8P | 307.6 |
| 2.92 | 856.6799 | PE 44:1_B | [M-H]- | C49H96NO8P | 298.9 |
| 2.79 | 854.6638 | PE 44:2 | [M-H]- | C49H94NO8P | 298.2 |
| 2.64 | 852.64806 | PE 44:3 | [M-H]- | C49H92NO8P | 297.6 |
| 2.63 | 852.64789 | PE 44:4_A | [M+H]+ | C49H90NO8P | 302 |
| 2.64 | 850.63361 | PE 44:4_B | [M-H]- | C49H90NO8P | 296.5 |
| 2.45 | 850.63139 | PE 44:5 | [M+H]+ | C49H88NO8P | 299.6 |
| 2.25 | 848.61584 | PE 44:6 | [M+H]+ | C49H86NO8P | 298.5 |
| 2.05 | 846.60072 | PE 44:7 | [M+H]+ | C49H84NO8P | 297.4 |
| 2.91 | 882.69439 | PE 46:2 | [M-H]- | C51H98NO8P | 303.3 |
| 2.63 | 876.64722 | PE 46:5 | [M-H]- | C51H92NO8P | 300.2 |
| 2.09 | 676.52758 | PE O-32:1_A | [M+H]+ | C37H74NO7P | 273.1 |
| 2.07 | 674.51213 | PE O-32:1_B | [M-H]- | C37H74NO7P | 263.3 |
| 2.33 | 704.55951 | PE O-34:1_A | [M+H]+, [M+Na]+ | C39H78NO7P | 279.6 |
| 2.3 | 702.54055 | PE O-34:1_B | [M-H]- | C39H78NO7P | 270 |
| 2.12 | 702.54241 | PE O-34:2_A | [M+H]+ | C39H76NO7P | 275.5 |
| 2.08 | 700.52823 | PE O-34:2_B | [M-H]- | C39H76NO7P | 267.9 |
| 1.9 | 698.51129 | PE O-34:3 | [M-H]- | C39H74NO7P | 266.1 |
| 2.22 | 716.55779 | PE O-35:2 | [M+H]+ | C40H78NO7P | 278.5 |
| 1.61 | 712.52585 | PE O-35:4_A | [M+H]+ | C40H74NO7P | 279.1 |
| 1.92 | 710.5131 | PE O-35:4_B | [M-H]- | C40H74NO7P | 268.7 |
| 2.32 | 728.55975 | PE O-36:2_A | [M-H]- | C41H80NO7P | 274.4 |
| 2.34 | 730.57362 | PE O-36:2_B | [M+H]+, [M+Na]+ | C41H80NO7P | 282 |
| 2.14 | 728.55752 | PE O-36:3_A | [M+H]+ | C41H78NO7P | 279.4 |
| 2.11 | 726.54295 | PE O-36:3_B | [M-H]- | C41H78NO7P | 272.4 |
| 2.04 | 724.52894 | PE O-36:4 | [M-H]- | C41H76NO7P | 271.5 |
| 1.88 | 724.52708 | PE O-36:5_A | [M+H]+, [M+Na]+ | C41H74NO7P | 274.9 |
| 1.86 | 722.51321 | PE O-36:5_B | [M-H]- | C41H74NO7P | 270.5 |
| 1.69 | 720.49786 | PE O-36:6 | [M-H]- | C41H72NO7P | 269.6 |
| 1.75 | 740.55835 | PE O-37:4 | [M+H]+ | C42H78NO7P | 284 |
| 1.95 | 736.52924 | PE O-37:5 | [M-H]- | C42H76NO7P | 274 |
| 1.69 | 732.49973 | PE O-37:7 | [M-H]- | C42H72NO7P | 272.5 |
| 2.56 | 758.60543 | PE O-38:2_A | [M+H]+, [M+Na]+ | C43H84NO7P | 288.4 |
| 2.53 | 756.59124 | PE O-38:2_B | [M-H]- | C43H84NO7P | 280.4 |
| 2.37 | 756.58887 | PE O-38:3_A | [M+H]+ | C43H82NO7P | 285.5 |
| 2.34 | 754.57556 | PE O-38:3_B | [M-H]- | C43H82NO7P | 279 |
| 2.12 | 752.55832 | PE O-38:5_A | [M+H]+, [M+Na]+ | C43H78NO7P | 282 |
| 2.09 | 750.54431 | PE O-38:5_B | [M-H]- | C43H78NO7P | 276.9 |
| 1.89 | 750.54293 | PE O-38:6_A | [M+H]+ | C43H76NO7P | 279.2 |
| 1.87 | 748.52815 | PE O-38:6_B | [M-H]- | C43H76NO7P | 275.2 |
| 1.79 | 746.51314 | PE O-38:7_A | [M-H]- | C43H74NO7P | 273.9 |
| 1.7 | 746.51308 | PE O-38:7_B | [M-H]- | C43H74NO7P | 274 |
| 2.3 | 766.57421 | PE O-39:4 | [M-H]- | C44H82NO7P | 281.1 |
| 2.11 | 764.56251 | PE O-39:5 | [M-H]- | C44H80NO7P | 279.7 |
| 2.72 | 784.62199 | PE O-40:2 | [M-H]- | C45H88NO7P | 286.6 |
| 2.58 | 784.61981 | PE O-40:3_A | [M+H]+ | C45H86NO7P | 292.2 |
| 2.54 | 782.60704 | PE O-40:3_B | [M-H]- | C45H86NO7P | 284.7 |
| 2.45 | 782.60429 | PE O-40:4_A | [M+H]+ | C45H84NO7P | 291 |
| 2.43 | 780.59127 | PE O-40:4_B | [M-H]- | C45H84NO7P | 284.1 |
| 2.28 | 780.58858 | PE O-40:5_A | [M+H]+, [M+Na]+ | C45H82NO7P | 288.6 |
| 2.25 | 778.57573 | PE O-40:5_B | [M-H]- | C45H82NO7P | 282.5 |
| 2.05 | 776.55842 | PE O-40:7_A | [M+H]+ | C45H78NO7P | 284.7 |
| 2.03 | 774.54462 | PE O-40:7_B | [M-H]- | C45H78NO7P | 280.8 |
| 1.83 | 774.54265 | PE O-40:8_A | [M+H]+ | C45H76NO7P | 281.7 |
| 1.81 | 772.52903 | PE O-40:8_B | [M-H]- | C45H76NO7P | 278.6 |
| 2.85 | 812.65063 | PE O-42:2 | [M-H]- | C47H92NO7P | 292.3 |
| 2.35 | 806.6025 | PE O-42:6 | [M+H]+ | C47H84NO7P | 292.9 |
| 2.17 | 802.57867 | PE O-42:7_A | [M-H]- | C47H82NO7P | 286.2 |
| 2.25 | 802.57508 | PE O-42:7_B | [M-H]- | C47H82NO7P | 286.6 |
| 1.98 | 802.57418 | PE O-42:8_A | [M+H]+ | C47H80NO7P | 288.3 |
| 1.95 | 800.55894 | PE O-42:8_B | [M-H]- | C47H80NO7P | 284.6 |
| 1.42 | 719.48726 | PG 32:1 | [M-H]- | C38H73O10P | 268.6 |
| 1.61 | 747.51885 | PG 34:1 | [M-H]- | C40H77O10P | 274.8 |
| 1.3 | 745.50214 | PG 34:2 | [M-H]- | C40H75O10P | 277.3 |
| 1.83 | 775.54863 | PG 36:1 | [M-H]- | C42H81O10P | 281 |
| 1.67 | 773.53468 | PG 36:2_A | [M-H]- | C42H79O10P | 280.1 |
| 1.49 | 773.53279 | PG 36:2_B | [M-H]- | C42H79O10P | 282.9 |
| 1.36 | 771.51683 | PG 36:3 | [M-H]- | C42H77O10P | 282.1 |
| 1.53 | 799.54469 | PG 38:3 | [M-H]- | C44H81O10P | 287.9 |
| 1.36 | 797.53223 | PG 38:4 | [M-H]- | C44H79O10P | 289.1 |
| 1.3 | 795.51961 | PG 38:5 | [M-H]- | C44H77O10P | 285.4 |
| 1.27 | 819.51774 | PG 40:7 | [M-H]- | C46H77O10P | 289.1 |
| 1.15 | 817.50355 | PG 40:8 | [M-H]- | C46H75O10P | 288 |
| 1.31 | 845.532 | PG 42:8 | [M-H]- | C48H79O10P | 293.8 |
| 1.08 | 865.5025 | PG 44:12 | [M-H]- | C50H75O10P | 294.3 |
| 1.36 | 807.50349 | PI 32:1 | [M-H]- | C41H77O13P | 283.7 |
| 1.56 | 835.53379 | PI 34:1_A | [M-H]- | C43H81O13P | 289.3 |
| 1.64 | 854.5742 | PI 34:1_B | [M+NH4]+, [M+H]+ | C43H81O13P | 298.8 |
| 1.39 | 833.51842 | PI 34:2 | [M-H]- | C43H79O13P | 288.1 |
| 1.26 | 831.50411 | PI 34:3 | [M-H]- | C43H77O13P | 286.4 |
| 1.56 | 866.57822 | PI 35:2 | [M+NH4]+ | C44H81O13P | 298.6 |
| 1.78 | 863.56541 | PI 36:1_A | [M-H]- | C45H85O13P | 296.1 |
| 1.87 | 882.60603 | PI 36:1_B | [M+NH4]+, [M+H]+ | C45H85O13P | 304.1 |
| 1.58 | 861.55 | PI 36:2_A | [M-H]- | C45H83O13P | 294.1 |
| 1.67 | 880.59065 | PI 36:2_B | [M+NH4]+, [M+H]+ | C45H83O13P | 301 |
| 1.45 | 859.53294 | PI 36:3 | [M-H]- | C45H81O13P | 292.8 |
| 1.38 | 857.51874 | PI 36:4_A | [M-H]- | C45H79O13P | 291.8 |
| 1.45 | 876.55943 | PI 36:4_B | [M+NH4]+, [M+H]+, [M+Na]+, [M+K]+ | C45H79O13P | 299.2 |
| 1.54 | 890.57203 | PI 37:4 | [M+NH4]+ | C46H81O13P | 301.1 |
| 1.82 | 889.58151 | PI 38:2 | [M-H]- | C47H87O13P | 300.4 |
| 1.69 | 887.56555 | PI 38:3_A | [M-H]- | C47H85O13P | 300 |
| 1.83 | 906.60681 | PI 38:3_B | [M+NH4]+, [M+H]+ | C47H85O13P | 306.8 |
| 1.58 | 885.55015 | PI 38:4_A | [M-H]- | C47H83O13P | 298.4 |
| 1.67 | 904.59057 | PI 38:4_B | [M+NH4]+, [M+H]+ | C47H83O13P | 304.6 |
| 1.4 | 883.53451 | PI 38:5_A | [M-H]- | C47H81O13P | 296.3 |
| 1.48 | 902.57489 | PI 38:5_B | [M+NH4]+, [M+K]+, [M+H]+, [M+Na]+ | C47H81O13P | 301.9 |
| 1.28 | 881.51853 | PI 38:6_A | [M-H]- | C47H79O13P | 294.9 |
| 1.34 | 900.55843 | PI 38:6_B | [M+NH4]+, [M+Na]+, [M+H]+ | C47H79O13P | 300.4 |
| 1.79 | 901.57997 | PI 39:3_A | [M-H]- | C48H87O13P | 302.4 |
| 1.88 | 920.62197 | PI 39:3_B | [M+NH4]+ | C48H87O13P | 309 |
| 1.58 | 916.58984 | PI 39:5 | [M+NH4]+ | C48H83O13P | 304.8 |
| 2.28 | 938.66431 | PI 40:1 | [M+NH4]+ | C49H93O13P | 315.1 |
| 1.7 | 913.58084 | PI 40:4_A | [M-H]- | C49H87O13P | 304.3 |
| 1.83 | 932.62169 | PI 40:4_B | [M+NH4]+ | C49H87O13P | 310 |
| 1.68 | 911.56547 | PI 40:5 | [M-H]- | C49H85O13P | 303.3 |
| 1.53 | 909.55008 | PI 40:6_A | [M-H]- | C49H83O13P | 301.7 |
| 1.49 | 928.58836 | PI 40:6_B | [M+NH4]+ | C49H83O13P | 305.1 |
| 1.6 | 928.59091 | PI 40:6_C | [M+NH4]+, [M+Na]+, [M+H]+ | C49H83O13P | 307.5 |
| 1.35 | 907.53394 | PI 40:7_A | [M-H]- | C49H81O13P | 299.7 |
| 1.43 | 926.57372 | PI 40:7_B | [M+NH4]+, [M+H]+, [M+Na]+ | C49H81O13P | 304.9 |
| 1.51 | 869.55467 | PI O-38:5 | [M-H]- | C47H83O12P | 295.3 |
| 1.48 | 633.45044 | PMeOH 30:0 | [M-H]- | C34H67O8P | 251.3 |
| 1.7 | 687.49696 | PMeOH 34:1 | [M-H]- | C38H73O8P | 262.7 |
| 1.57 | 734.49861 | PS 32:0 | [M-H]- | C38H74NO10P | 274 |
| 1.4 | 732.48265 | PS 32:1 | [M-H]- | C38H72NO10P | 272 |
| 1.79 | 762.52978 | PS 34:0 | [M-H]- | C40H78NO10P | 280.9 |
| 1.6 | 760.51339 | PS 34:1 | [M-H]- | C40H76NO10P | 278.3 |
| 1.43 | 758.49923 | PS 34:2_A | [M-H]- | C40H74NO10P | 276.3 |
| 1.52 | 760.51091 | PS 34:2_B | [M+H]+ | C40H74NO10P | 281.5 |
| 1.82 | 788.54512 | PS 36:1 | [M-H]- | C42H80NO10P | 284.8 |
| 1.63 | 786.52911 | PS 36:2_A | [M-H]- | C42H78NO10P | 282 |
| 1.73 | 788.54321 | PS 36:2_B | [M+H]+ | C42H78NO10P | 287.5 |
| 1.49 | 784.51414 | PS 36:3 | [M-H]- | C42H76NO10P | 281 |
| 1.69 | 800.54577 | PS 37:2 | [M-H]- | C43H80NO10P | 285.6 |
| 2.15 | 818.59187 | PS 38:1_A | [M+H]+ | C44H84NO10P | 297 |
| 2.05 | 816.57623 | PS 38:1_B | [M-H]- | C44H84NO10P | 290 |
| 1.96 | 816.57484 | PS 38:2_A | [M+H]+ | C44H82NO10P | 294.1 |
| 1.84 | 814.56131 | PS 38:2_B | [M-H]- | C44H82NO10P | 289.8 |
| 1.81 | 814.5603 | PS 38:3_A | [M+H]+ | C44H80NO10P | 291.2 |
| 1.7 | 812.54314 | PS 38:3_B | [M-H]- | C44H80NO10P | 287.9 |
| 1.59 | 810.52968 | PS 38:4 | [M-H]- | C44H78NO10P | 285.7 |
| 2.26 | 844.60716 | PS 40:1_A | [M-H]- | C46H88NO10P | 296.4 |
| 2.38 | 846.62147 | PS 40:1_B | [M+H]+, [M+Na]+ | C46H88NO10P | 301.4 |
| 2.16 | 844.60724 | PS 40:2_A | [M+H]+ | C46H86NO10P | 300 |
| 2.05 | 842.59117 | PS 40:2_B | [M-H]-, [M+CH3]- | C46H86NO10P | 295.8 |
| 1.95 | 840.57485 | PS 40:3_A | [M-H]- | C46H84NO10P | 292.7 |
| 2.05 | 842.59115 | PS 40:3_B | [M+H]+ | C46H84NO10P | 298.3 |
| 1.77 | 838.5602 | PS 40:4_A | [M-H]- | C46H82NO10P | 291.6 |
| 1.88 | 840.57563 | PS 40:4_B | [M+H]+ | C46H82NO10P | 295.7 |
| 1.63 | 836.54611 | PS 40:5_A | [M-H]- | C46H80NO10P | 293.3 |
| 1.73 | 838.55809 | PS 40:5_B | [M+H]+ | C46H80NO10P | 292.6 |
| 1.66 | 836.54322 | PS 40:6 | [M+H]+ | C46H78NO10P | 291.9 |
| 1.46 | 834.52764 | PS 40:7 | [M+H]+, [M+Na]+ | C46H76NO10P | 288.8 |
| 2.27 | 858.61926 | PS 41:2 | [M+H]+ | C47H88NO10P | 302.1 |
| 2.59 | 874.65249 | PS 42:1_A | [M+H]+, [M+Na]+ | C48H92NO10P | 304.6 |
| 2.47 | 872.63965 | PS 42:1_B | [M-H]- | C48H92NO10P | 301.6 |
| 2.37 | 872.6384 | PS 42:2 | [M+H]+, [M+Na]+ | C48H90NO10P | 303.3 |
| 2.08 | 868.60888 | PS 42:3_A | [M-H]- | C48H88NO10P | 299.9 |
| 2.19 | 870.62256 | PS 42:3_B | [M+H]+ | C48H88NO10P | 302.6 |
| 2.47 | 886.65159 | PS 43:2 | [M+H]+ | C49H92NO10P | 308.2 |
| 2.59 | 900.66984 | PS 44:2_A | [M+H]+, [M+Na]+ | C50H94NO10P | 311.1 |
| 2.46 | 898.65229 | PS 44:2_B | [M-H]- | C50H94NO10P | 305.7 |
| 2.39 | 898.65005 | PS 44:3 | [M+H]+ | C50H92NO10P | 309.1 |
| 2.25 | 896.63554 | PS 44:4_A | [M+H]+ | C50H90NO10P | 306.3 |
| 2.48 | 894.61969 | PS 44:4_B | [M-H]- | C50H90NO10P | 301.6 |
| 1.85 | 788.58037 | PS O-37:1 | [M-H]- | C43H84NO9P | 290.5 |
| 1.19 | 647.51271 | SM 30:1;2O | [M+H]+ | C35H71N2O6P | 275.4 |
| 1.3 | 661.52814 | SM 31:1;2O | [M+H]+ | C36H73N2O6P | 278.4 |
| 1.5 | 677.55945 | SM 32:0;2O | [M+H]+ | C37H77N2O6P | 284.8 |
| 1.42 | 675.54409 | SM 32:1;2O | [M+H]+ | C37H75N2O6P | 281 |
| 1.35 | 691.54066 | SM 32:1;3O | [M+H]+ | C37H75N2O7P | 287.7 |
| 1.23 | 673.5263 | SM 32:2;2O | [M+H]+ | C37H73N2O6P | 277.2 |
| 1.53 | 689.5587 | SM 33:1;2O | [M+H]+, [M+Na]+ | C38H77N2O6P | 284 |
| 1.35 | 687.54237 | SM 33:2;2O | [M+H]+ | C38H75N2O6P | 280.4 |
| 1.74 | 705.58953 | SM 34:0;2O | [M+H]+ | C39H81N2O6P | 290 |
| 1.65 | 703.57518 | SM 34:1;2O | [M+H]+ | C39H79N2O6P | 286.6 |
| 1.58 | 719.56956 | SM 34:1;3O | [M+H]+ | C39H79N2O7P | 292.9 |
| 1.46 | 701.55955 | SM 34:2;2O | [M+H]+, [M+Na]+, [M+K]+ | C39H77N2O6P | 283.3 |
| 1.39 | 717.55411 | SM 34:2;3O | [M+H]+, [M+Na]+ | C39H77N2O7P | 289.9 |
| 1.77 | 717.59024 | SM 35:1;2O | [M+H]+ | C40H81N2O6P | 289.3 |
| 1.89 | 731.606 | SM 36:1;2O | [M+H]+ | C41H83N2O6P | 293.1 |
| 1.68 | 729.58928 | SM 36:2;2O | [M+H]+ | C41H81N2O6P | 289.2 |
| 2.15 | 759.63708 | SM 38:1;2O | [M+H]+ | C43H87N2O6P | 298.5 |
| 2.37 | 787.66807 | SM 40:1;2O | [M+H]+, [M+K]+, [M+Na]+ | C45H91N2O6P | 302.2 |
| 2.14 | 785.65245 | SM 40:2;2O | [M+H]+ | C45H89N2O6P | 300.9 |
| 2.5 | 801.68359 | SM 41:1;2O | [M+H]+ | C46H93N2O6P | 305.8 |
| 2.26 | 799.6676 | SM 41:2;2O | [M+H]+ | C46H91N2O6P | 303.2 |
| 1.81 | 797.65052 | SM 41:3;2O | [M+H]+ | C46H89N2O6P | 298.7 |
| 2.6 | 815.70002 | SM 42:1;2O | [M+H]+, [M+K]+, [M+Na]+ | C47H95N2O6P | 308.3 |
| 2.26 | 831.6938 | SM 42:1;3O | [M+H]+ | C47H95N2O7P | 309.2 |
| 2.37 | 813.68447 | SM 42:2;2O | [M+H]+ | C47H93N2O6P | 305.9 |
| 2.17 | 811.66781 | SM 42:3;2O | [M+H]+ | C47H91N2O6P | 303.2 |
| 2 | 809.64911 | SM 42:4;2O | [M+H]+ | C47H89N2O6P | 301.1 |
| 2.7 | 829.715 | SM 43:1;2O | [M+H]+ | C48H97N2O6P | 311.3 |
| 2.48 | 827.6992 | SM 43:2;2O | [M+H]+, [M+Na]+ | C48H95N2O6P | 309.2 |
| 2.29 | 825.68379 | SM 43:3;2O | [M+H]+ | C48H93N2O6P | 306.2 |
| 2.5 | 823.66823 | SM 43:4;2O | [M+H]+ | C48H91N2O6P | 305.9 |
| 2.8 | 843.73092 | SM 44:1;2O | [M+H]+, [M+Na]+ | C49H99N2O6P | 314.1 |
| 2.59 | 841.71459 | SM 44:2;2O | [M+H]+, [M+Na]+, [M+K]+ | C49H97N2O6P | 312.4 |
| 2.39 | 839.69818 | SM 44:3;2O | [M+H]+, [M+Na]+ | C49H95N2O6P | 309.3 |
| 2.22 | 837.68171 | SM 44:4;2O | [M+H]+ | C49H93N2O6P | 306.7 |
| 3.25 | 850.78459 | TG 50:1 | [M+NH4]+ [M+Na]+ [M+K]+ | C53H100O6 | 317.8 |
| 3.2 | 848.76909 | TG 50:2 | [M+NH4]+ [M+K]+ [M+Na]+ | C53H98O6 | 316.1 |
| 3.1 | 844.73781 | TG 50:4 | [M+NH4]+ [M+K]+[M+Na]+ | C53H94O6 | 312.8 |
| 3.2 | 874.78423 | TG 52:3 | [M+NH4]+ [M+Na]+ [M+K]+ | C55H100O6 | 319.6 |
| 3.1 | 870.75362 | TG 52:5 | [M+NH4]+ [M+Na]+ | C55H96O6 | 317 |
| 3 | 866.72268 | TG 52:7 | [M+NH4]+ | C55H92O6 | 314.3 |
| 3.27 | 890.81395 | TG 53:2 | [M+NH4]+ [M+K]+ | C56H104O6 | 324.6 |
| 3.22 | 888.79835 | TG 53:3 | [M+NH4]+ [M+K]+ [M+Na]+ | C56H102O6 | 322.7 |
| 3.17 | 898.78367 | TG 54:5 | [M+NH4]+ [M+Na]+ | C57H100O6 | 323.1 |
| 3.08 | 894.75122 | TG 54:7 | [M+NH4]+ [M+K]+ | C57H96O6 | 320.5 |
| 3.02 | 892.7379 | TG 54:8 | [M+NH4]+ [M+K]+ | C57H94O6 | 318.5 |
| 3.27 | 916.82945 | TG 55:3 | [M+NH4]+ [M+Na]+ | C58H106O6 | 328.2 |
| 3.24 | 914.81534 | TG 55:4 | [M+NH4]+ | C58H104O6 | 327.9 |
| 3.1 | 908.77091 | TG 55:7 | [M+NH4]+ | C58H98O6 | 324 |
| 3.26 | 928.82958 | TG 56:4 | [M+NH4]+ [M+K]+ | C59H106O6 | 329.4 |
| 3.15 | 922.78466 | TG 56:7 | [M+NH4]+ [M+Na]+ [M+K]+ | C59H100O6 | 326.4 |
| 3.09 | 920.76808 | TG 56:8 | [M+NH4]+ [M+K]+[M+Na]+ | C59H98O6 | 324.4 |
| 3.03 | 918.75322 | TG 56:9 | [M+NH4]+ | C59H96O6 | 322.3 |
| 3.28 | 942.84611 | TG 57:4 | [M+NH4]+ | C60H108O6 | 332.6 |
| 3.17 | 936.79992 | TG 57:7 | [M+NH4]+ | C60H102O6 | 328.9 |
| 3.12 | 934.78617 | TG 57:8 | [M+NH4]+ | C60H100O6 | 327.3 |
| 3 | 942.7557 | TG 58:11 | [M+NH4]+ | C61H96O6 | 324.8 |
| 3.22 | 952.83057 | TG 58:6 | [M+NH4]+ | C61H106O6 | 332.7 |
| 3.15 | 948.79978 | TG 58:8 | [M+NH4]+ [M+Na]+ [M+K]+ | C61H102O6 | 329.8 |
| 3.1 | 946.78214 | TG 58:9 | [M+NH4]+ [M+Na]+ [M+K]+ | C61H100O6 | 327.7 |
| 3.12 | 972.79968 | TG 60:10 | [M+NH4]+ [M+Na]+ | C63H102O6 | 331.9 |
| 3.07 | 970.78512 | TG 60:11 | [M+NH4]+ [M+Na]+ [M+K]+ | C63H100O6 | 330.5 |
| 3.31 | 982.87507 | TG 60:5 | [M+NH4]+ | C63H112O6 | 338.8 |
| 3.27 | 980.86254 | TG 60:6 | [M+NH4]+ | C63H110O6 | 338.3 |
| 3.23 | 978.8477 | TG 60:7 | [M+NH4]+ | C63H108O6 | 336.7 |
| 3.19 | 976.83119 | TG 60:8 | [M+NH4]+ | C63H106O6 | 334.8 |
| 3.16 | 974.8163 | TG 60:9 | [M+NH4]+ | C63H104O6 | 333.2 |
| 3.17 | 1000.83235 | TG 62:10 | [M+NH4]+ | C65H106O6 | 337.3 |
| 3.12 | 998.81524 | TG 62:11 | [M+NH4]+ | C65H104O6 | 335.8 |
| 3.37 | 1012.9253 | TG 62:4 | [M+NH4]+ [M+K]+ [M+Na]+ | C65H118O6 | 345.3 |
| 3.31 | 1008.89665 | TG 62:6 | [M+NH4]+ | C65H114O6 | 343.2 |
| 3.28 | 1006.87824 | TG 62:7 | [M+NH4]+ | C65H112O6 | 341.7 |
| 3.24 | 1004.86384 | TG 62:8 | [M+NH4]+ | C65H110O6 | 340.1 |
| 3.41 | 1040.95592 | TG 64:4 | [M+NH4]+ | C67H122O6 | 349.9 |
| 3.32 | 1034.91099 | TG 64:7 | [M+NH4]+ | C67H116O6 | 346.7 |
| 3.32 | 836.80448 | TG O-50:1 | [M+NH4]+ [M+Na]+ | C53H102O5 | 316.4 |
| 3.36 | 864.83548 | TG O-52:1 | [M+NH4]+ [M+Na]+ | C55H106O5 | 322.2 |

**Table S3:** Annotated proteins by nano-LC-HRMS

| **Protein FDR** | **Accession** | **Description** | **Coverage [%]** | **Gene Symbol** |
| --- | --- | --- | --- | --- |
| High | P35579 | Myosin-9 [OS=Homo sapiens] | 56 | MYH9 |
| High | P21333 | Filamin-A [OS=Homo sapiens] | 55 | FLNA |
| High | P08670 | Vimentin [OS=Homo sapiens] | 92 | VIM |
| High | Q9Y490 | Talin-1 [OS=Homo sapiens] | 41 | TLN1 |
| High | P49327 | Fatty acid synthase [OS=Homo sapiens] | 38 | FASN |
| High | P06733 | Alpha-enolase [OS=Homo sapiens] | 87 | ENO1 |
| High | P14618 | Pyruvate kinase PKM [OS=Homo sapiens] | 69 | PKM |
| High | P60709 | Actin, cytoplasmic 1 [OS=Homo sapiens] | 79 | ACTB |
| High | P13639 | Elongation factor 2 [OS=Homo sapiens] | 64 | EEF2 |
| High | Q00610 | Clathrin heavy chain 1 [OS=Homo sapiens] | 40 | CLTC |
| High | P07437 | Tubulin beta chain [OS=Homo sapiens] | 82 | TUBB |
| High | P08238 | Heat shock protein HSP 90-beta [OS=Homo sapiens] | 57 | HSP90AB1 |
| High | P07900 | Heat shock protein HSP 90-alpha [OS=Homo sapiens] | 51 | HSP90AA1 |
| High | P07355 | Annexin A2 [OS=Homo sapiens] | 73 | ANXA2 |
| High | P60174 | Triosephosphate isomerase [OS=Homo sapiens] | 82 | TPI1 |
| High | Q9BQE3 | Tubulin alpha-1C chain [OS=Homo sapiens] | 65 | TUBA1C |
| High | Q71U36 | Tubulin alpha-1A chain [OS=Homo sapiens] | 70 | TUBA1A |
| High | P68371 | Tubulin beta-4B chain [OS=Homo sapiens] | 79 | TUBB4B |
| High | P10809 | 60 kDa heat shock protein, mitochondrial [OS=Homo sapiens] | 60 | HSPD1 |
| High | P68104 | Elongation factor 1-alpha 1 [OS=Homo sapiens] | 64 | EEF1A1 |
| High | P04350 | Tubulin beta-4A chain [OS=Homo sapiens] | 71 | TUBB4A |
| High | Q14204 | Cytoplasmic dynein 1 heavy chain 1 [OS=Homo sapiens] | 15 | DYNC1H1 |
| High | P55072 | Transitional endoplasmic reticulum ATPase [OS=Homo sapiens] | 49 | VCP |
| High | P02751 | Fibronectin [OS=Homo sapiens] | 25 | FN1 |
| High | P04406 | Glyceraldehyde-3-phosphate dehydrogenase [OS=Homo sapiens] | 74 | GAPDH |
| High | O43707 | Alpha-actinin-4 [OS=Homo sapiens] | 55 | ACTN4 |
| High | P04075 | Fructose-bisphosphate aldolase A [OS=Homo sapiens] | 78 | ALDOA |
| High | Q9BVA1 | Tubulin beta-2B chain [OS=Homo sapiens] | 58 | TUBB2B |
| High | P11142 | Heat shock cognate 71 kDa protein [OS=Homo sapiens] | 54 | HSPA8 |
| High | P78527 | DNA-dependent protein kinase catalytic subunit [OS=Homo sapiens] | 14 | PRKDC |
| High | P19338 | Nucleolin [OS=Homo sapiens] | 39 | NCL |
| High | P68133 | Actin, alpha skeletal muscle [OS=Homo sapiens] | 42 | ACTA1 |
| High | P68366 | Tubulin alpha-4A chain [OS=Homo sapiens] | 50 | TUBA4A |
| High | P12814 | Alpha-actinin-1 [OS=Homo sapiens] | 45 | ACTN1 |
| High | P02545 | Prelamin-A/C [OS=Homo sapiens] | 45 | LMNA |
| High | P16403 | Histone H1.2 [OS=Homo sapiens] | 51 | H1-2 |
| High | P67936 | Tropomyosin alpha-4 chain [OS=Homo sapiens] | 67 | TPM4 |
| High | P00558 | Phosphoglycerate kinase 1 [OS=Homo sapiens] | 68 | PGK1 |
| High | Q5QNW6 | Histone H2B type 2-F [OS=Homo sapiens] | 73 | H2BC18 |
| High | Q6FI13 | Histone H2A type 2-A [OS=Homo sapiens] | 76 | H2AC18; H2AC19 |
| High | P22626 | Heterogeneous nuclear ribonucleoproteins A2/B1 [OS=Homo sapiens] | 50 | HNRNPA2B1 |
| High | P22314 | Ubiquitin-like modifier-activating enzyme 1 [OS=Homo sapiens] | 34 | UBA1 |
| High | Q7L7L0 | Histone H2A type 3 [OS=Homo sapiens] | 76 | H2AC25 |
| High | O60814 | Histone H2B type 1-K [OS=Homo sapiens] | 73 | H2BC12 |
| High | P07237 | Protein disulfide-isomerase [OS=Homo sapiens] | 56 | P4HB |
| High | Q16778 | Histone H2B type 2-E [OS=Homo sapiens] | 73 | H2BC21 |
| High | P10412 | Histone H1.4 [OS=Homo sapiens] | 45 | H1-4 |
| High | P16402 | Histone H1.3 [OS=Homo sapiens] | 43 | H1-3 |
| High | Q8IUE6 | Histone H2A type 2-B [OS=Homo sapiens] | 71 | H2AC21 |
| High | P36578 | 60S ribosomal protein L4 [OS=Homo sapiens] | 50 | RPL4 |
| High | P16104 | Histone H2AX [OS=Homo sapiens] | 59 | H2AX |
| High | P30101 | Protein disulfide-isomerase A3 [OS=Homo sapiens] | 45 | PDIA3 |
| High | P14625 | Endoplasmin [OS=Homo sapiens] | 38 | HSP90B1 |
| High | Q14697 | Neutral alpha-glucosidase AB [OS=Homo sapiens] | 35 | GANAB |
| High | P62805 | Histone H4 [OS=Homo sapiens] | 63 | H4C1; H4C11; H4C12; H4C13; H4C14; H4C15; H4C16; H4C2; H4C3; H4C4; H4C5; H4C6; H4C8; H4C9 |
| High | P61978 | Heterogeneous nuclear ribonucleoprotein K [OS=Homo sapiens] | 50 | HNRNPK |
| High | P08758 | Annexin A5 [OS=Homo sapiens] | 72 | ANXA5 |
| High | P06576 | ATP synthase subunit beta, mitochondrial [OS=Homo sapiens] | 51 | ATP5F1B |
| High | Q13509 | Tubulin beta-3 chain [OS=Homo sapiens] | 43 | TUBB3 |
| High | P09651 | Heterogeneous nuclear ribonucleoprotein A1 [OS=Homo sapiens] | 50 | HNRNPA1 |
| High | P00338 | L-lactate dehydrogenase A chain [OS=Homo sapiens] | 76 | LDHA |
| High | P12956 | X-ray repair cross-complementing protein 6 [OS=Homo sapiens] | 40 | XRCC6 |
| High | P04083 | Annexin A1 [OS=Homo sapiens] | 53 | ANXA1 |
| High | P11021 | Endoplasmic reticulum chaperone BiP [OS=Homo sapiens] | 43 | HSPA5 |
| High | Q14974 | Importin subunit beta-1 [OS=Homo sapiens] | 38 | KPNB1 |
| High | Q07065 | Cytoskeleton-associated protein 4 [OS=Homo sapiens] | 56 | CKAP4 |
| High | P50454 | Serpin H1 [OS=Homo sapiens] | 46 | SERPINH1 |
| High | P26038 | Moesin [OS=Homo sapiens] | 43 | MSN |
| High | P25705 | ATP synthase subunit alpha, mitochondrial [OS=Homo sapiens] | 33 | ATP5F1A |
| High | P26599 | Polypyrimidine tract-binding protein 1 [OS=Homo sapiens] | 48 | PTBP1 |
| High | P63104 | 14-3-3 protein zeta/delta [OS=Homo sapiens] | 53 | YWHAZ |
| High | Q09666 | Neuroblast differentiation-associated protein AHNAK [OS=Homo sapiens] | 16 | AHNAK |
| High | P06748 | Nucleophosmin [OS=Homo sapiens] | 58 | NPM1 |
| High | P52292 | Importin subunit alpha-1 [OS=Homo sapiens] | 57 | KPNA2 |
| High | P16401 | Histone H1.5 [OS=Homo sapiens] | 42 | H1-5 |
| High | P29401 | Transketolase [OS=Homo sapiens] | 46 | TKT |
| High | P09382 | Galectin-1 [OS=Homo sapiens] | 81 | LGALS1 |
| High | P46940 | Ras GTPase-activating-like protein IQGAP1 [OS=Homo sapiens] | 19 | IQGAP1 |
| High | P07951 | Tropomyosin beta chain [OS=Homo sapiens] | 33 | TPM2 |
| High | P62937 | Peptidyl-prolyl cis-trans isomerase A [OS=Homo sapiens] | 82 | PPIA |
| High | P0DMV9 | Heat shock 70 kDa protein 1B [OS=Homo sapiens] | 46 | HSPA1B |
| High | P27797 | Calreticulin [OS=Homo sapiens] | 56 | CALR |
| High | P35580 | Myosin-10 [OS=Homo sapiens] | 11 | MYH10 |
| High | P53396 | ATP-citrate synthase [OS=Homo sapiens] | 22 | ACLY |
| High | P13489 | Ribonuclease inhibitor [OS=Homo sapiens] | 59 | RNH1 |
| High | Q92616 | eIF-2-alpha kinase activator GCN1 [OS=Homo sapiens] | 11 | GCN1 |
| High | Q9BUF5 | Tubulin beta-6 chain [OS=Homo sapiens] | 46 | TUBB6 |
| High | P06753 | Tropomyosin alpha-3 chain [OS=Homo sapiens] | 34 | TPM3 |
| High | Q00839 | Heterogeneous nuclear ribonucleoprotein U [OS=Homo sapiens] | 30 | HNRNPU |
| High | P68431 | Histone H3.1 [OS=Homo sapiens] | 71 | H3C1; H3C10; H3C11; H3C12; H3C2; H3C3; H3C4; H3C6; H3C7; H3C8 |
| High | Q71DI3 | Histone H3.2 [OS=Homo sapiens] | 71 | H3C13; H3C14; H3C15 |
| High | Q14315 | Filamin-C [OS=Homo sapiens] | 11 | FLNC |
| High | Q99497 | Parkinson disease protein 7 [OS=Homo sapiens] | 63 | PARK7 |
| High | P55060 | Exportin-2 [OS=Homo sapiens] | 28 | CSE1L |
| High | P13010 | X-ray repair cross-complementing protein 5 [OS=Homo sapiens] | 40 | XRCC5 |
| High | P60842 | Eukaryotic initiation factor 4A-I [OS=Homo sapiens] | 61 | EIF4A1 |
| High | P23528 | Cofilin-1 [OS=Homo sapiens] | 63 | CFL1 |
| High | P78371 | T-complex protein 1 subunit beta [OS=Homo sapiens] | 51 | CCT2 |
| High | P09493 | Tropomyosin alpha-1 chain [OS=Homo sapiens] | 26 | TPM1 |
| High | Q15149 | Plectin [OS=Homo sapiens] | 8 | PLEC |
| High | P62424 | 60S ribosomal protein L7a [OS=Homo sapiens] | 58 | RPL7A |
| High | P07195 | L-lactate dehydrogenase B chain [OS=Homo sapiens] | 60 | LDHB |
| High | O75369 | Filamin-B [OS=Homo sapiens] | 12 | FLNB |
| High | P04792 | Heat shock protein beta-1 [OS=Homo sapiens] | 71 | HSPB1 |
| High | P40227 | T-complex protein 1 subunit zeta [OS=Homo sapiens] | 28 | CCT6A |
| High | P23246 | Splicing factor, proline- and glutamine-rich [OS=Homo sapiens] | 23 | SFPQ |
| High | P49588 | Alanine--tRNA ligase, cytoplasmic [OS=Homo sapiens] | 21 | AARS1 |
| High | Q15084 | Protein disulfide-isomerase A6 [OS=Homo sapiens] | 36 | PDIA6 |
| High | P18669 | Phosphoglycerate mutase 1 [OS=Homo sapiens] | 56 | PGAM1 |
| High | P17987 | T-complex protein 1 subunit alpha [OS=Homo sapiens] | 47 | TCP1 |
| High | O43175 | D-3-phosphoglycerate dehydrogenase [OS=Homo sapiens] | 43 | PHGDH |
| High | Q02878 | 60S ribosomal protein L6 [OS=Homo sapiens] | 54 | RPL6 |
| High | P37802 | Transgelin-2 [OS=Homo sapiens] | 71 | TAGLN2 |
| High | P62258 | 14-3-3 protein epsilon [OS=Homo sapiens] | 40 | YWHAE |
| High | P04844 | Dolichyl-diphosphooligosaccharide--protein glycosyltransferase subunit 2 [OS=Homo sapiens] | 33 | RPN2 |
| High | P30153 | Serine/threonine-protein phosphatase 2A 65 kDa regulatory subunit A alpha isoform [OS=Homo sapiens] | 30 | PPP2R1A |
| High | O00299 | Chloride intracellular channel protein 1 [OS=Homo sapiens] | 67 | CLIC1 |
| High | Q71UI9 | Histone H2A.V [OS=Homo sapiens] | 63 | H2AZ2 |
| High | P50991 | T-complex protein 1 subunit delta [OS=Homo sapiens] | 34 | CCT4 |
| High | P07737 | Profilin-1 [OS=Homo sapiens] | 74 | PFN1 |
| High | Q08211 | ATP-dependent RNA helicase A [OS=Homo sapiens] | 19 | DHX9 |
| High | P63244 | Receptor of activated protein C kinase 1 [OS=Homo sapiens] | 70 | RACK1 |
| High | P49411 | Elongation factor Tu, mitochondrial [OS=Homo sapiens] | 37 | TUFM |
| High | P31946 | 14-3-3 protein beta/alpha [OS=Homo sapiens] | 52 | YWHAB |
| High | P50395 | Rab GDP dissociation inhibitor beta [OS=Homo sapiens] | 29 | GDI2 |
| High | P27348 | 14-3-3 protein theta [OS=Homo sapiens] | 50 | YWHAQ |
| High | P35232 | Prohibitin 1 [OS=Homo sapiens] | 69 | PHB1 |
| High | P26641 | Elongation factor 1-gamma [OS=Homo sapiens] | 30 | EEF1G |
| High | P84243 | Histone H3.3 [OS=Homo sapiens] | 60 | H3-3A; H3-3B |
| High | P27816 | Microtubule-associated protein 4 [OS=Homo sapiens] | 19 | MAP4 |
| High | Q01518 | Adenylyl cyclase-associated protein 1 [OS=Homo sapiens] | 34 | CAP1 |
| High | P15311 | Ezrin [OS=Homo sapiens] | 31 | EZR |
| High | P62241 | 40S ribosomal protein S8 [OS=Homo sapiens] | 59 | RPS8 |
| High | Q12905 | Interleukin enhancer-binding factor 2 [OS=Homo sapiens] | 53 | ILF2 |
| High | Q86VP6 | Cullin-associated NEDD8-dissociated protein 1 [OS=Homo sapiens] | 17 | CAND1 |
| High | O00159 | Unconventional myosin-Ic [OS=Homo sapiens] | 26 | MYO1C |
| High | Q13200 | 26S proteasome non-ATPase regulatory subunit 2 [OS=Homo sapiens] | 18 | PSMD2 |
| High | P46782 | 40S ribosomal protein S5 [OS=Homo sapiens] | 48 | RPS5 |
| High | Q06830 | Peroxiredoxin-1 [OS=Homo sapiens] | 58 | PRDX1 |
| High | P13797 | Plastin-3 [OS=Homo sapiens] | 26 | PLS3 |
| High | P20700 | Lamin-B1 [OS=Homo sapiens] | 26 | LMNB1 |
| High | Q13813 | Spectrin alpha chain, non-erythrocytic 1 [OS=Homo sapiens] | 12 | SPTAN1 |
| High | P40926 | Malate dehydrogenase, mitochondrial [OS=Homo sapiens] | 42 | MDH2 |
| High | Q562R1 | Beta-actin-like protein 2 [OS=Homo sapiens] | 25 | ACTBL2 |
| High | O75643 | U5 small nuclear ribonucleoprotein 200 kDa helicase [OS=Homo sapiens] | 8 | SNRNP200 |
| High | Q96P70 | Importin-9 [OS=Homo sapiens] | 13 | IPO9 |
| High | P31150 | Rab GDP dissociation inhibitor alpha [OS=Homo sapiens] | 27 | GDI1 |
| High | P48643 | T-complex protein 1 subunit epsilon [OS=Homo sapiens] | 29 | CCT5 |
| High | P08133 | Annexin A6 [OS=Homo sapiens] | 28 | ANXA6 |
| High | P61313 | 60S ribosomal protein L15 [OS=Homo sapiens] | 45 | RPL15 |
| High | Q99832 | T-complex protein 1 subunit eta [OS=Homo sapiens] | 31 | CCT7 |
| High | Q96AG4 | Leucine-rich repeat-containing protein 59 [OS=Homo sapiens] | 42 | LRRC59 |
| High | P31939 | Bifunctional purine biosynthesis protein ATIC [OS=Homo sapiens] | 33 | ATIC |
| High | P38646 | Stress-70 protein, mitochondrial [OS=Homo sapiens] | 25 | HSPA9 |
| High | Q04917 | 14-3-3 protein eta [OS=Homo sapiens] | 48 | YWHAH |
| High | P52209 | 6-phosphogluconate dehydrogenase, decarboxylating [OS=Homo sapiens] | 28 | PGD |
| High | P63241 | Eukaryotic translation initiation factor 5A-1 [OS=Homo sapiens] | 51 | EIF5A |
| High | O60664 | Perilipin-3 [OS=Homo sapiens] | 51 | PLIN3 |
| High | P67809 | Y-box-binding protein 1 [OS=Homo sapiens] | 35 | YBX1 |
| High | O75533 | Splicing factor 3B subunit 1 [OS=Homo sapiens] | 13 | SF3B1 |
| High | P17844 | Probable ATP-dependent RNA helicase DDX5 [OS=Homo sapiens] | 32 | DDX5 |
| High | P62917 | 60S ribosomal protein L8 [OS=Homo sapiens] | 21 | RPL8 |
| High | P23396 | 40S ribosomal protein S3 [OS=Homo sapiens] | 56 | RPS3 |
| High | Q01082 | Spectrin beta chain, non-erythrocytic 1 [OS=Homo sapiens] | 8 | SPTBN1 |
| High | P18124 | 60S ribosomal protein L7 [OS=Homo sapiens] | 46 | RPL7 |
| High | P05388 | 60S acidic ribosomal protein P0 [OS=Homo sapiens] | 47 | RPLP0 |
| High | Q07020 | 60S ribosomal protein L18 [OS=Homo sapiens] | 41 | RPL18 |
| High | O15067 | Phosphoribosylformylglycinamidine synthase [OS=Homo sapiens] | 17 | PFAS |
| High | Q9BWD1 | Acetyl-CoA acetyltransferase, cytosolic [OS=Homo sapiens] | 32 | ACAT2 |
| High | P08865 | 40S ribosomal protein SA [OS=Homo sapiens] | 33 | RPSA |
| High | P01889 | HLA class I histocompatibility antigen, B alpha chain [OS=Homo sapiens] | 32 | HLA-B |
| High | P62701 | 40S ribosomal protein S4, X isoform [OS=Homo sapiens] | 47 | RPS4X |
| High | P42167 | Lamina-associated polypeptide 2, isoforms beta/gamma [OS=Homo sapiens] | 38 | TMPO |
| High | P29692 | Elongation factor 1-delta [OS=Homo sapiens] | 38 | EEF1D |
| High | P26640 | Valine--tRNA ligase [OS=Homo sapiens] | 15 | VARS1 |
| High | P62081 | 40S ribosomal protein S7 [OS=Homo sapiens] | 51 | RPS7 |
| High | Q99623 | Prohibitin-2 [OS=Homo sapiens] | 44 | PHB2 |
| High | P27824 | Calnexin [OS=Homo sapiens] | 25 | CANX |
| High | Q13838 | Spliceosome RNA helicase DDX39B [OS=Homo sapiens] | 30 | DDX39B |
| High | P10321 | HLA class I histocompatibility antigen, C alpha chain [OS=Homo sapiens] | 33 | HLA-C |
| High | O43390 | Heterogeneous nuclear ribonucleoprotein R [OS=Homo sapiens] | 26 | HNRNPR |
| High | P30041 | Peroxiredoxin-6 [OS=Homo sapiens] | 52 | PRDX6 |
| High | P51991 | Heterogeneous nuclear ribonucleoprotein A3 [OS=Homo sapiens] | 32 | HNRNPA3 |
| High | O75083 | WD repeat-containing protein 1 [OS=Homo sapiens] | 33 | WDR1 |
| High | P09874 | Poly [ADP-ribose] polymerase 1 [OS=Homo sapiens] | 14 | PARP1 |
| High | O00571 | ATP-dependent RNA helicase DDX3X [OS=Homo sapiens] | 15 | DDX3X |
| High | P62826 | GTP-binding nuclear protein Ran [OS=Homo sapiens] | 44 | RAN |
| High | P09104 | Gamma-enolase [OS=Homo sapiens] | 23 | ENO2 |
| High | P05204 | Non-histone chromosomal protein HMG-17 [OS=Homo sapiens] | 54 | HMGN2 |
| High | P61981 | 14-3-3 protein gamma [OS=Homo sapiens] | 35 | YWHAG |
| High | Q15365 | Poly(rC)-binding protein 1 [OS=Homo sapiens] | 43 | PCBP1 |
| High | P12236 | ADP/ATP translocase 3 [OS=Homo sapiens] | 39 | SLC25A6 |
| High | P06744 | Glucose-6-phosphate isomerase [OS=Homo sapiens] | 30 | GPI |
| High | O14980 | Exportin-1 [OS=Homo sapiens] | 14 | XPO1 |
| High | Q9ULV4 | Coronin-1C [OS=Homo sapiens] | 26 | CORO1C |
| High | O60506 | Heterogeneous nuclear ribonucleoprotein Q [OS=Homo sapiens] | 27 | SYNCRIP |
| High | Q12906 | Interleukin enhancer-binding factor 3 [OS=Homo sapiens] | 18 | ILF3 |
| High | P30044 | Peroxiredoxin-5, mitochondrial [OS=Homo sapiens] | 54 | PRDX5 |
| High | Q15233 | Non-POU domain-containing octamer-binding protein [OS=Homo sapiens] | 28 | NONO |
| High | P41250 | Glycine--tRNA ligase [OS=Homo sapiens] | 16 | GARS1 |
| High | Q9BSJ8 | Extended synaptotagmin-1 [OS=Homo sapiens] | 16 | ESYT1 |
| High | P05387 | 60S acidic ribosomal protein P2 [OS=Homo sapiens] | 77 | RPLP2 |
| High | P61247 | 40S ribosomal protein S3a [OS=Homo sapiens] | 34 | RPS3A |
| High | P54819 | Adenylate kinase 2, mitochondrial [OS=Homo sapiens] | 29 | AK2 |
| High | Q9UJZ1 | Stomatin-like protein 2, mitochondrial [OS=Homo sapiens] | 29 | STOML2 |
| High | O60701 | UDP-glucose 6-dehydrogenase [OS=Homo sapiens] | 31 | UGDH |
| High | Q15366 | Poly(rC)-binding protein 2 [OS=Homo sapiens] | 41 | PCBP2 |
| High | Q5TEC6 | Histone H3-7 [OS=Homo sapiens] | 35 | H3-7 |
| High | P43243 | Matrin-3 [OS=Homo sapiens] | 11 | MATR3 |
| High | P42166 | Lamina-associated polypeptide 2, isoform alpha [OS=Homo sapiens] | 30 | TMPO |
| High | P31943 | Heterogeneous nuclear ribonucleoprotein H [OS=Homo sapiens] | 24 | HNRNPH1 |
| High | Q9UHB6 | LIM domain and actin-binding protein 1 [OS=Homo sapiens] | 23 | LIMA1 |
| High | Q7KZF4 | Staphylococcal nuclease domain-containing protein 1 [OS=Homo sapiens] | 16 | SND1 |
| High | P39023 | 60S ribosomal protein L3 [OS=Homo sapiens] | 29 | RPL3 |
| High | P30050 | 60S ribosomal protein L12 [OS=Homo sapiens] | 45 | RPL12 |
| High | P00387 | NADH-cytochrome b5 reductase 3 [OS=Homo sapiens] | 40 | CYB5R3 |
| High | Q01813 | ATP-dependent 6-phosphofructokinase, platelet type [OS=Homo sapiens] | 25 | PFKP |
| High | P52272 | Heterogeneous nuclear ribonucleoprotein M [OS=Homo sapiens] | 15 | HNRNPM |
| High | P05141 | ADP/ATP translocase 2 [OS=Homo sapiens] | 34 | SLC25A5 |
| High | Q99829 | Copine-1 [OS=Homo sapiens] | 12 | CPNE1 |
| High | P49748 | Very long-chain specific acyl-CoA dehydrogenase, mitochondrial [OS=Homo sapiens] | 25 | ACADVL |
| High | P18206 | Vinculin [OS=Homo sapiens] | 12 | VCL |
| High | P62277 | 40S ribosomal protein S13 [OS=Homo sapiens] | 32 | RPS13 |
| High | Q14103 | Heterogeneous nuclear ribonucleoprotein D0 [OS=Homo sapiens] | 25 | HNRNPD |
| High | P11940 | Polyadenylate-binding protein 1 [OS=Homo sapiens] | 18 | PABPC1 |
| High | P04439 | HLA class I histocompatibility antigen, A alpha chain [OS=Homo sapiens] | 28 | HLA-A |
| High | Q6NZI2 | Caveolae-associated protein 1 [OS=Homo sapiens] | 14 | CAVIN1 |
| High | P22234 | Bifunctional phosphoribosylaminoimidazole carboxylase/phosphoribosylaminoimidazole succinocarboxamide synthetase [OS=Homo sapiens] | 28 | PAICS |
| High | O95373 | Importin-7 [OS=Homo sapiens] | 13 | IPO7 |
| High | P22392 | Nucleoside diphosphate kinase B [OS=Homo sapiens] | 57 | NME2 |
| High | P45974 | Ubiquitin carboxyl-terminal hydrolase 5 [OS=Homo sapiens] | 19 | USP5 |
| High | P52597 | Heterogeneous nuclear ribonucleoprotein F [OS=Homo sapiens] | 22 | HNRNPF |
| High | P30154 | Serine/threonine-protein phosphatase 2A 65 kDa regulatory subunit A beta isoform [OS=Homo sapiens] | 14 | PPP2R1B |
| High | P12111 | Collagen alpha-3(VI) chain [OS=Homo sapiens] | 6 | COL6A3 |
| High | P60660 | Myosin light polypeptide 6 [OS=Homo sapiens] | 48 | MYL6 |
| High | O14950 | Myosin regulatory light chain 12B [OS=Homo sapiens] | 56 | MYL12B |
| High | Q13263 | Transcription intermediary factor 1-beta [OS=Homo sapiens] | 22 | TRIM28 |
| High | P84103 | Serine/arginine-rich splicing factor 3 [OS=Homo sapiens] | 45 | SRSF3 |
| High | P15880 | 40S ribosomal protein S2 [OS=Homo sapiens] | 33 | RPS2 |
| High | P47756 | F-actin-capping protein subunit beta [OS=Homo sapiens] | 26 | CAPZB |
| High | P52565 | Rho GDP-dissociation inhibitor 1 [OS=Homo sapiens] | 35 | ARHGDIA |
| High | P34897 | Serine hydroxymethyltransferase, mitochondrial [OS=Homo sapiens] | 33 | SHMT2 |
| High | Q05682 | Caldesmon [OS=Homo sapiens] | 14 | CALD1 |
| High | P32969 | 60S ribosomal protein L9 [OS=Homo sapiens] | 39 | RPL9; RPL9P7; RPL9P8; RPL9P9 |
| High | P49368 | T-complex protein 1 subunit gamma [OS=Homo sapiens] | 33 | CCT3 |
| High | P62987 | Ubiquitin-60S ribosomal protein L40 [OS=Homo sapiens] | 48 | UBA52 |
| High | P18085 | ADP-ribosylation factor 4 [OS=Homo sapiens] | 67 | ARF4 |
| High | P30086 | Phosphatidylethanolamine-binding protein 1 [OS=Homo sapiens] | 73 | PEBP1 |
| High | P50990 | T-complex protein 1 subunit theta [OS=Homo sapiens] | 22 | CCT8 |
| High | Q14764 | Major vault protein [OS=Homo sapiens] | 17 | MVP |
| High | O00148 | ATP-dependent RNA helicase DDX39A [OS=Homo sapiens] | 25 | DDX39A |
| High | Q99714 | 3-hydroxyacyl-CoA dehydrogenase type-2 [OS=Homo sapiens] | 28 | HSD17B10 |
| High | P61604 | 10 kDa heat shock protein, mitochondrial [OS=Homo sapiens] | 67 | HSPE1 |
| High | P04843 | Dolichyl-diphosphooligosaccharide--protein glycosyltransferase subunit 1 [OS=Homo sapiens] | 22 | RPN1 |
| High | P0CG39 | POTE ankyrin domain family member J [OS=Homo sapiens] | 6 | POTEJ |
| High | Q8WUM4 | Programmed cell death 6-interacting protein [OS=Homo sapiens] | 13 | PDCD6IP |
| High | P09429 | High mobility group protein B1 [OS=Homo sapiens] | 32 | HMGB1 |
| High | P40925 | Malate dehydrogenase, cytoplasmic [OS=Homo sapiens] | 37 | MDH1 |
| High | P26373 | 60S ribosomal protein L13 [OS=Homo sapiens] | 39 | RPL13 |
| High | O60684 | Importin subunit alpha-7 [OS=Homo sapiens] | 21 | KPNA6 |
| High | O75475 | PC4 and SFRS1-interacting protein [OS=Homo sapiens] | 21 | PSIP1 |
| High | P61158 | Actin-related protein 3 [OS=Homo sapiens] | 29 | ACTR3 |
| High | O43852 | Calumenin [OS=Homo sapiens] | 29 | CALU |
| High | P46776 | 60S ribosomal protein L27a [OS=Homo sapiens] | 32 | RPL27A |
| High | P38919 | Eukaryotic initiation factor 4A-III [OS=Homo sapiens] | 25 | EIF4A3 |
| High | Q14152 | Eukaryotic translation initiation factor 3 subunit A [OS=Homo sapiens] | 8 | EIF3A |
| High | Q92499 | ATP-dependent RNA helicase DDX1 [OS=Homo sapiens] | 16 | DDX1 |
| High | Q15942 | Zyxin [OS=Homo sapiens] | 21 | ZYX |
| High | P05386 | 60S acidic ribosomal protein P1 [OS=Homo sapiens] | 52 | RPLP1 |
| High | P62280 | 40S ribosomal protein S11 [OS=Homo sapiens] | 46 | RPS11 |
| High | Q9UL46 | Proteasome activator complex subunit 2 [OS=Homo sapiens] | 28 | PSME2 |
| High | O00410 | Importin-5 [OS=Homo sapiens] | 15 | IPO5 |
| High | Q15056 | Eukaryotic translation initiation factor 4H [OS=Homo sapiens] | 26 | EIF4H |
| High | P47897 | Glutamine--tRNA ligase [OS=Homo sapiens] | 11 | QARS1 |
| High | P16949 | Stathmin [OS=Homo sapiens] | 37 | STMN1 |
| High | P16152 | Carbonyl reductase [NADPH] 1 [OS=Homo sapiens] | 39 | CBR1 |
| High | P42704 | Leucine-rich PPR motif-containing protein, mitochondrial [OS=Homo sapiens] | 8 | LRPPRC |
| High | Q92841 | Probable ATP-dependent RNA helicase DDX17 [OS=Homo sapiens] | 16 | DDX17 |
| High | P62829 | 60S ribosomal protein L23 [OS=Homo sapiens] | 40 | RPL23 |
| High | P31948 | Stress-induced-phosphoprotein 1 [OS=Homo sapiens] | 18 | STIP1 |
| High | Q00341 | Vigilin [OS=Homo sapiens] | 9 | HDLBP |
| High | Q9Y696 | Chloride intracellular channel protein 4 [OS=Homo sapiens] | 34 | CLIC4 |
| High | P61586 | Transforming protein RhoA [OS=Homo sapiens] | 27 | RHOA |
| High | P51149 | Ras-related protein Rab-7a [OS=Homo sapiens] | 47 | RAB7A |
| High | P09211 | Glutathione S-transferase P [OS=Homo sapiens] | 40 | GSTP1 |
| High | P04637 | Cellular tumor antigen p53 [OS=Homo sapiens] | 13 | TP53 |
| High | P26368 | Splicing factor U2AF 65 kDa subunit [OS=Homo sapiens] | 14 | U2AF2 |
| High | P00505 | Aspartate aminotransferase, mitochondrial [OS=Homo sapiens] | 21 | GOT2 |
| High | P30084 | Enoyl-CoA hydratase, mitochondrial [OS=Homo sapiens] | 21 | ECHS1 |
| High | Q96QK1 | Vacuolar protein sorting-associated protein 35 [OS=Homo sapiens] | 9 | VPS35 |
| High | P46821 | Microtubule-associated protein 1B [OS=Homo sapiens] | 6 | MAP1B |
| High | P62136 | Serine/threonine-protein phosphatase PP1-alpha catalytic subunit [OS=Homo sapiens] | 29 | PPP1CA |
| High | P43686 | 26S proteasome regulatory subunit 6B [OS=Homo sapiens] | 23 | PSMC4 |
| High | Q5SSJ5 | Heterochromatin protein 1-binding protein 3 [OS=Homo sapiens] | 16 | HP1BP3 |
| High | P22102 | Trifunctional purine biosynthetic protein adenosine-3 [OS=Homo sapiens] | 13 | GART |
| High | P33993 | DNA replication licensing factor MCM7 [OS=Homo sapiens] | 14 | MCM7 |
| High | Q9Y4L1 | Hypoxia up-regulated protein 1 [OS=Homo sapiens] | 11 | HYOU1 |
| High | P49736 | DNA replication licensing factor MCM2 [OS=Homo sapiens] | 14 | MCM2 |
| High | O00303 | Eukaryotic translation initiation factor 3 subunit F [OS=Homo sapiens] | 17 | EIF3F |
| High | P08134 | Rho-related GTP-binding protein RhoC [OS=Homo sapiens] | 34 | RHOC |
| High | Q9Y678 | Coatomer subunit gamma-1 [OS=Homo sapiens] | 9 | COPG1 |
| High | Q04637 | Eukaryotic translation initiation factor 4 gamma 1 [OS=Homo sapiens] | 7 | EIF4G1 |
| High | P49321 | Nuclear autoantigenic sperm protein [OS=Homo sapiens] | 20 | NASP |
| High | P09972 | Fructose-bisphosphate aldolase C [OS=Homo sapiens] | 21 | ALDOC |
| High | Q10567 | AP-1 complex subunit beta-1 [OS=Homo sapiens] | 6 | AP1B1 |
| High | P33176 | Kinesin-1 heavy chain [OS=Homo sapiens] | 6 | KIF5B |
| High | Q16531 | DNA damage-binding protein 1 [OS=Homo sapiens] | 6 | DDB1 |
| High | Q92945 | Far upstream element-binding protein 2 [OS=Homo sapiens] | 21 | KHSRP |
| High | P62263 | 40S ribosomal protein S14 [OS=Homo sapiens] | 24 | RPS14 |
| High | Q9Y224 | RNA transcription, translation and transport factor protein [OS=Homo sapiens] | 21 | RTRAF |
| High | P22695 | Cytochrome b-c1 complex subunit 2, mitochondrial [OS=Homo sapiens] | 36 | UQCRC2 |
| High | P33991 | DNA replication licensing factor MCM4 [OS=Homo sapiens] | 9 | MCM4 |
| High | Q86V81 | THO complex subunit 4 [OS=Homo sapiens] | 35 | ALYREF |
| High | P15531 | Nucleoside diphosphate kinase A [OS=Homo sapiens] | 47 | NME1 |
| High | P07910 | Heterogeneous nuclear ribonucleoproteins C1/C2 [OS=Homo sapiens] | 23 | HNRNPC |
| High | Q16555 | Dihydropyrimidinase-related protein 2 [OS=Homo sapiens] | 21 | DPYSL2 |
| High | P68036 | Ubiquitin-conjugating enzyme E2 L3 [OS=Homo sapiens] | 46 | UBE2L3 |
| High | P09543 | 2',3'-cyclic-nucleotide 3'-phosphodiesterase [OS=Homo sapiens] | 21 | CNP |
| High | Q13148 | TAR DNA-binding protein 43 [OS=Homo sapiens] | 28 | TARDBP |
| High | P40261 | Nicotinamide N-methyltransferase [OS=Homo sapiens] | 42 | NNMT |
| High | P48047 | ATP synthase subunit O, mitochondrial [OS=Homo sapiens] | 31 | ATP5PO |
| High | P63010 | AP-2 complex subunit beta [OS=Homo sapiens] | 6 | AP2B1 |
| High | P13674 | Prolyl 4-hydroxylase subunit alpha-1 [OS=Homo sapiens] | 12 | P4HA1 |
| High | P55209 | Nucleosome assembly protein 1-like 1 [OS=Homo sapiens] | 26 | NAP1L1 |
| High | P62888 | 60S ribosomal protein L30 [OS=Homo sapiens] | 41 | RPL30 |
| High | P12004 | Proliferating cell nuclear antigen [OS=Homo sapiens] | 30 | PCNA |
| High | P49915 | GMP synthase [glutamine-hydrolyzing] [OS=Homo sapiens] | 16 | GMPS |
| High | P60891 | Ribose-phosphate pyrophosphokinase 1 [OS=Homo sapiens] | 18 | PRPS1 |
| High | P42224 | Signal transducer and activator of transcription 1-alpha/beta [OS=Homo sapiens] | 14 | STAT1 |
| High | Q15393 | Splicing factor 3B subunit 3 [OS=Homo sapiens] | 9 | SF3B3 |
| High | Q15404 | Ras suppressor protein 1 [OS=Homo sapiens] | 24 | RSU1 |
| High | P35637 | RNA-binding protein FUS [OS=Homo sapiens] | 7 | FUS |
| High | O14979 | Heterogeneous nuclear ribonucleoprotein D-like [OS=Homo sapiens] | 19 | HNRNPDL |
| High | P36871 | Phosphoglucomutase-1 [OS=Homo sapiens] | 13 | PGM1 |
| High | P42766 | 60S ribosomal protein L35 [OS=Homo sapiens] | 38 | RPL35 |
| High | P80723 | Brain acid soluble protein 1 [OS=Homo sapiens] | 44 | BASP1 |
| High | P52294 | Importin subunit alpha-5 [OS=Homo sapiens] | 17 | KPNA1 |
| High | P11413 | Glucose-6-phosphate 1-dehydrogenase [OS=Homo sapiens] | 20 | G6PD |
| High | P62753 | 40S ribosomal protein S6 [OS=Homo sapiens] | 36 | RPS6 |
| High | P14866 | Heterogeneous nuclear ribonucleoprotein L [OS=Homo sapiens] | 23 | HNRNPL |
| High | O75390 | Citrate synthase, mitochondrial [OS=Homo sapiens] | 26 | CS |
| High | Q15019 | Septin-2 [OS=Homo sapiens] | 27 | SEPTIN2 |
| High | Q9UQ80 | Proliferation-associated protein 2G4 [OS=Homo sapiens] | 19 | PA2G4 |
| High | Q9Y265 | RuvB-like 1 [OS=Homo sapiens] | 24 | RUVBL1 |
| High | Q8NE71 | ATP-binding cassette sub-family F member 1 [OS=Homo sapiens] | 13 | ABCF1 |
| High | P61160 | Actin-related protein 2 [OS=Homo sapiens] | 19 | ACTR2 |
| High | P62266 | 40S ribosomal protein S23 [OS=Homo sapiens] | 49 | RPS23 |
| High | P61088 | Ubiquitin-conjugating enzyme E2 N [OS=Homo sapiens] | 49 | UBE2N |
| High | Q01105 | Protein SET [OS=Homo sapiens] | 20 | SET |
| High | Q9P0L0 | Vesicle-associated membrane protein-associated protein A [OS=Homo sapiens] | 18 | VAPA |
| High | P05023 | Sodium/potassium-transporting ATPase subunit alpha-1 [OS=Homo sapiens] | 9 | ATP1A1 |
| High | O95573 | Fatty acid CoA ligase Acsl3 [OS=Homo sapiens] | 21 | ACSL3 |
| High | P60953 | Cell division control protein 42 homolog [OS=Homo sapiens] | 36 | CDC42 |
| High | P15559 | NAD(P)H dehydrogenase [quinone] 1 [OS=Homo sapiens] | 26 | NQO1 |
| High | P26583 | High mobility group protein B2 [OS=Homo sapiens] | 21 | HMGB2 |
| High | P62906 | 60S ribosomal protein L10a [OS=Homo sapiens] | 28 | RPL10A |
| High | P62140 | Serine/threonine-protein phosphatase PP1-beta catalytic subunit [OS=Homo sapiens] | 18 | PPP1CB |
| High | Q5VYK3 | Proteasome adapter and scaffold protein ECM29 [OS=Homo sapiens] | 5 | ECPAS |
| High | P48735 | Isocitrate dehydrogenase [NADP], mitochondrial [OS=Homo sapiens] | 17 | IDH2 |
| High | P32119 | Peroxiredoxin-2 [OS=Homo sapiens] | 36 | PRDX2 |
| High | P56537 | Eukaryotic translation initiation factor 6 [OS=Homo sapiens] | 33 | EIF6 |
| High | Q6P2Q9 | Pre-mRNA-processing-splicing factor 8 [OS=Homo sapiens] | 4 | PRPF8 |
| High | P07741 | Adenine phosphoribosyltransferase [OS=Homo sapiens] | 36 | APRT |
| High | Q14019 | Coactosin-like protein [OS=Homo sapiens] | 47 | COTL1 |
| High | P46783 | 40S ribosomal protein S10 [OS=Homo sapiens] | 29 | RPS10 |
| High | P29966 | Myristoylated alanine-rich C-kinase substrate [OS=Homo sapiens] | 20 | MARCKS |
| High | O43169 | Cytochrome b5 type B [OS=Homo sapiens] | 31 | CYB5B |
| High | Q96T76 | MMS19 nucleotide excision repair protein homolog [OS=Homo sapiens] | 9 | MMS19 |
| High | O43237 | Cytoplasmic dynein 1 light intermediate chain 2 [OS=Homo sapiens] | 17 | DYNC1LI2 |
| High | Q07021 | Complement component 1 Q subcomponent-binding protein, mitochondrial [OS=Homo sapiens] | 32 | C1QBP |
| High | Q15021 | Condensin complex subunit 1 [OS=Homo sapiens] | 6 | NCAPD2 |
| High | O00469 | Procollagen-lysine,2-oxoglutarate 5-dioxygenase 2 [OS=Homo sapiens] | 16 | PLOD2 |
| High | P06132 | Uroporphyrinogen decarboxylase [OS=Homo sapiens] | 27 | UROD |
| High | P06493 | Cyclin-dependent kinase 1 [OS=Homo sapiens] | 30 | CDK1 |
| High | O75367 | Core histone macro-H2A.1 [OS=Homo sapiens] | 23 | MACROH2A1 |
| High | P43487 | Ran-specific GTPase-activating protein [OS=Homo sapiens] | 23 | RANBP1 |
| High | Q9NR30 | Nucleolar RNA helicase 2 [OS=Homo sapiens] | 12 | DDX21 |
| High | O43399 | Tumor protein D54 [OS=Homo sapiens] | 26 | TPD52L2 |
| High | Q92522 | Histone H1.10 [OS=Homo sapiens] | 14 | H1-10 |
| High | P46781 | 40S ribosomal protein S9 [OS=Homo sapiens] | 28 | RPS9 |
| High | P24534 | Elongation factor 1-beta [OS=Homo sapiens] | 40 | EEF1B2 |
| High | P61163 | Alpha-centractin [OS=Homo sapiens] | 25 | ACTR1A |
| High | P53621 | Coatomer subunit alpha [OS=Homo sapiens] | 9 | COPA |
| High | Q16629 | Serine/arginine-rich splicing factor 7 [OS=Homo sapiens] | 20 | SRSF7 |
| High | Q969E4 | Transcription elongation factor A protein-like 3 [OS=Homo sapiens] | 12 | TCEAL3 |
| High | P35241 | Radixin [OS=Homo sapiens] | 15 | RDX |
| High | Q07955 | Serine/arginine-rich splicing factor 1 [OS=Homo sapiens] | 30 | SRSF1 |
| High | B5ME19 | Eukaryotic translation initiation factor 3 subunit C-like protein [OS=Homo sapiens] | 11 | EIF3CL |
| High | Q58FF6 | Putative heat shock protein HSP 90-beta 4 [OS=Homo sapiens] | 11 | HSP90AB4P |
| High | P07814 | Bifunctional glutamate/proline--tRNA ligase [OS=Homo sapiens] | 8 | EPRS1 |
| High | O14880 | Microsomal glutathione S-transferase 3 [OS=Homo sapiens] | 28 | MGST3 |
| High | Q03252 | Lamin-B2 [OS=Homo sapiens] | 12 | LMNB2 |
| High | P02452 | Collagen alpha-1(I) chain [OS=Homo sapiens] | 6 | COL1A1 |
| High | P38159 | RNA-binding motif protein, X chromosome [OS=Homo sapiens] | 21 | RBMX |
| High | P05556 | Integrin beta-1 [OS=Homo sapiens] | 9 | ITGB1 |
| High | P31689 | DnaJ homolog subfamily A member 1 [OS=Homo sapiens] | 12 | DNAJA1 |
| High | P06396 | Gelsolin [OS=Homo sapiens] | 13 | GSN |
| High | P25205 | DNA replication licensing factor MCM3 [OS=Homo sapiens] | 10 | MCM3 |
| High | Q9Y262 | Eukaryotic translation initiation factor 3 subunit L [OS=Homo sapiens] | 10 | EIF3L |
| High | P39019 | 40S ribosomal protein S19 [OS=Homo sapiens] | 37 | RPS19 |
| High | Q16881 | Thioredoxin reductase 1, cytoplasmic [OS=Homo sapiens] | 14 | TXNRD1 |
| High | P62269 | 40S ribosomal protein S18 [OS=Homo sapiens] | 34 | RPS18 |
| High | Q9Y4G6 | Talin-2 [OS=Homo sapiens] | 5 | TLN2 |
| High | Q92688 | Acidic leucine-rich nuclear phosphoprotein 32 family member B [OS=Homo sapiens] | 43 | ANP32B |
| High | P52907 | F-actin-capping protein subunit alpha-1 [OS=Homo sapiens] | 22 | CAPZA1 |
| High | P62249 | 40S ribosomal protein S16 [OS=Homo sapiens] | 34 | RPS16 |
| High | Q9H9B4 | Sideroflexin-1 [OS=Homo sapiens] | 21 | SFXN1 |
| High | O60610 | Protein diaphanous homolog 1 [OS=Homo sapiens] | 5 | DIAPH1 |
| High | Q99798 | Aconitate hydratase, mitochondrial [OS=Homo sapiens] | 12 | ACO2 |
| High | O00629 | Importin subunit alpha-3 [OS=Homo sapiens] | 11 | KPNA4 |
| High | Q9NR31 | GTP-binding protein SAR1a [OS=Homo sapiens] | 35 | SAR1A |
| High | P62314 | Small nuclear ribonucleoprotein Sm D1 [OS=Homo sapiens] | 29 | SNRPD1 |
| High | O60888 | Protein CutA [OS=Homo sapiens] | 33 | CUTA |
| High | Q07666 | KH domain-containing, RNA-binding, signal transduction-associated protein 1 [OS=Homo sapiens] | 17 | KHDRBS1 |
| High | P61204 | ADP-ribosylation factor 3 [OS=Homo sapiens] | 38 | ARF3 |
| High | Q8NBS9 | Thioredoxin domain-containing protein 5 [OS=Homo sapiens] | 15 | TXNDC5 |
| High | Q86VI3 | Ras GTPase-activating-like protein IQGAP3 [OS=Homo sapiens] | 5 | IQGAP3 |
| High | P30048 | Thioredoxin-dependent peroxide reductase, mitochondrial [OS=Homo sapiens] | 34 | PRDX3 |
| High | Q15843 | NEDD8 [OS=Homo sapiens] | 41 | NEDD8 |
| High | P11216 | Glycogen phosphorylase, brain form [OS=Homo sapiens] | 8 | PYGB |
| High | Q9UII2 | ATPase inhibitor, mitochondrial [OS=Homo sapiens] | 18 | ATP5IF1 |
| High | Q15691 | Microtubule-associated protein RP/EB family member 1 [OS=Homo sapiens] | 25 | MAPRE1 |
| High | P61619 | Protein transport protein Sec61 subunit alpha isoform 1 [OS=Homo sapiens] | 14 | SEC61A1 |
| High | P31930 | Cytochrome b-c1 complex subunit 1, mitochondrial [OS=Homo sapiens] | 25 | UQCRC1 |
| High | P08708 | 40S ribosomal protein S17 [OS=Homo sapiens] | 49 | RPS17 |
| High | Q13283 | Ras GTPase-activating protein-binding protein 1 [OS=Homo sapiens] | 17 | G3BP1 |
| High | P27635 | 60S ribosomal protein L10 [OS=Homo sapiens] | 37 | RPL10 |
| High | P46777 | 60S ribosomal protein L5 [OS=Homo sapiens] | 18 | RPL5 |
| High | P42771 | Cyclin-dependent kinase inhibitor 2A [OS=Homo sapiens] | 29 | CDKN2A |
| High | Q7L1Q6 | eIF5-mimic protein 2 [OS=Homo sapiens] | 7 | BZW1 |
| High | P54577 | Tyrosine--tRNA ligase, cytoplasmic [OS=Homo sapiens] | 16 | YARS1 |
| High | Q9Y281 | Cofilin-2 [OS=Homo sapiens] | 31 | CFL2 |
| High | P14174 | Macrophage migration inhibitory factor [OS=Homo sapiens] | 13 | MIF |
| High | P17980 | 26S proteasome regulatory subunit 6A [OS=Homo sapiens] | 15 | PSMC3 |
| High | O95782 | AP-2 complex subunit alpha-1 [OS=Homo sapiens] | 11 | AP2A1 |
| High | O43809 | Cleavage and polyadenylation specificity factor subunit 5 [OS=Homo sapiens] | 13 | NUDT21 |
| High | Q9BTT0 | Acidic leucine-rich nuclear phosphoprotein 32 family member E [OS=Homo sapiens] | 25 | ANP32E |
| High | P34932 | Heat shock 70 kDa protein 4 [OS=Homo sapiens] | 12 | HSPA4 |
| High | P14314 | Glucosidase 2 subunit beta [OS=Homo sapiens] | 13 | PRKCSH |
| High | P50914 | 60S ribosomal protein L14 [OS=Homo sapiens] | 20 | RPL14 |
| High | Q9BQG0 | Myb-binding protein 1A [OS=Homo sapiens] | 8 | MYBBP1A |
| High | Q9NR45 | Sialic acid synthase [OS=Homo sapiens] | 28 | NANS |
| High | O96008 | Mitochondrial import receptor subunit TOM40 homolog [OS=Homo sapiens] | 17 | TOMM40 |
| High | Q15029 | 116 kDa U5 small nuclear ribonucleoprotein component [OS=Homo sapiens] | 9 | EFTUD2 |
| High | P17174 | Aspartate aminotransferase, cytoplasmic [OS=Homo sapiens] | 15 | GOT1 |
| High | P12268 | Inosine-5'-monophosphate dehydrogenase 2 [OS=Homo sapiens] | 22 | IMPDH2 |
| High | Q99536 | Synaptic vesicle membrane protein VAT-1 homolog [OS=Homo sapiens] | 15 | VAT1 |
| High | P68400 | Casein kinase II subunit alpha [OS=Homo sapiens] | 10 | CSNK2A1 |
| High | P37837 | Transaldolase [OS=Homo sapiens] | 17 | TALDO1 |
| High | O94925 | Glutaminase kidney isoform, mitochondrial [OS=Homo sapiens] | 11 | GLS |
| High | Q96D15 | Reticulocalbin-3 [OS=Homo sapiens] | 25 | RCN3 |
| High | P27695 | DNA-(apurinic or apyrimidinic site) endonuclease [OS=Homo sapiens] | 23 | APEX1 |
| High | P10599 | Thioredoxin [OS=Homo sapiens] | 46 | TXN |
| High | Q9P2E9 | Ribosome-binding protein 1 [OS=Homo sapiens] | 8 | RRBP1 |
| High | Q12931 | Heat shock protein 75 kDa, mitochondrial [OS=Homo sapiens] | 13 | TRAP1 |
| High | P25787 | Proteasome subunit alpha type-2 [OS=Homo sapiens] | 26 | PSMA2 |
| High | Q16401 | 26S proteasome non-ATPase regulatory subunit 5 [OS=Homo sapiens] | 11 | PSMD5 |
| High | O60884 | DnaJ homolog subfamily A member 2 [OS=Homo sapiens] | 17 | DNAJA2 |
| High | Q06210 | Glutamine--fructose-6-phosphate aminotransferase [isomerizing] 1 [OS=Homo sapiens] | 11 | GFPT1 |
| High | P23284 | Peptidyl-prolyl cis-trans isomerase B [OS=Homo sapiens] | 25 | PPIB |
| High | P39687 | Acidic leucine-rich nuclear phosphoprotein 32 family member A [OS=Homo sapiens] | 31 | ANP32A |
| High | O00151 | PDZ and LIM domain protein 1 [OS=Homo sapiens] | 28 | PDLIM1 |
| High | Q13765 | Nascent polypeptide-associated complex subunit alpha [OS=Homo sapiens] | 27 | NACA |
| High | P24752 | Acetyl-CoA acetyltransferase, mitochondrial [OS=Homo sapiens] | 10 | ACAT1 |
| High | P61224 | Ras-related protein Rap-1b [OS=Homo sapiens] | 14 | RAP1B |
| High | O00429 | Dynamin-1-like protein [OS=Homo sapiens] | 7 | DNM1L |
| High | Q16222 | UDP-N-acetylhexosamine pyrophosphorylase [OS=Homo sapiens] | 8 | UAP1 |
| High | P21796 | Voltage-dependent anion-selective channel protein 1 [OS=Homo sapiens] | 29 | VDAC1 |
| High | P11388 | DNA topoisomerase 2-alpha [OS=Homo sapiens] | 3 | TOP2A |
| High | Q14258 | E3 ubiquitin/ISG15 ligase TRIM25 [OS=Homo sapiens] | 8 | TRIM25 |
| High | Q99729 | Heterogeneous nuclear ribonucleoprotein A/B [OS=Homo sapiens] | 16 | HNRNPAB |
| High | O43818 | U3 small nucleolar RNA-interacting protein 2 [OS=Homo sapiens] | 11 | RRP9 |
| High | P21399 | Cytoplasmic aconitate hydratase [OS=Homo sapiens] | 11 | ACO1 |
| High | P84098 | 60S ribosomal protein L19 [OS=Homo sapiens] | 20 | RPL19 |
| High | Q00325 | Phosphate carrier protein, mitochondrial [OS=Homo sapiens] | 14 | SLC25A3 |
| High | P39656 | Dolichyl-diphosphooligosaccharide--protein glycosyltransferase 48 kDa subunit [OS=Homo sapiens] | 13 | DDOST |
| High | O95336 | 6-phosphogluconolactonase [OS=Homo sapiens] | 39 | PGLS |
| High | P18621 | 60S ribosomal protein L17 [OS=Homo sapiens] | 29 | RPL17 |
| High | P36776 | Lon protease homolog, mitochondrial [OS=Homo sapiens] | 12 | LONP1 |
| High | Q7Z6Z7 | E3 ubiquitin-protein ligase HUWE1 [OS=Homo sapiens] | 2 | HUWE1 |
| High | P04632 | Calpain small subunit 1 [OS=Homo sapiens] | 15 | CAPNS1 |
| High | P78417 | Glutathione S-transferase omega-1 [OS=Homo sapiens] | 17 | GSTO1 |
| High | P25398 | 40S ribosomal protein S12 [OS=Homo sapiens] | 43 | RPS12 |
| High | P40939 | Trifunctional enzyme subunit alpha, mitochondrial [OS=Homo sapiens] | 11 | HADHA |
| High | O75874 | Isocitrate dehydrogenase [NADP] cytoplasmic [OS=Homo sapiens] | 13 | IDH1 |
| High | P36957 | Dihydrolipoyllysine-residue succinyltransferase component of 2-oxoglutarate dehydrogenase complex, mitochondrial [OS=Homo sapiens] | 13 | DLST |
| High | P31942 | Heterogeneous nuclear ribonucleoprotein H3 [OS=Homo sapiens] | 11 | HNRNPH3 |
| High | Q14566 | DNA replication licensing factor MCM6 [OS=Homo sapiens] | 10 | MCM6 |
| High | Q92974 | Rho guanine nucleotide exchange factor 2 [OS=Homo sapiens] | 7 | ARHGEF2 |
| High | Q16576 | Histone-binding protein RBBP7 [OS=Homo sapiens] | 17 | RBBP7 |
| High | Q96AE4 | Far upstream element-binding protein 1 [OS=Homo sapiens] | 10 | FUBP1 |
| High | Q14247 | Src substrate cortactin [OS=Homo sapiens] | 8 | CTTN |
| High | Q14498 | RNA-binding protein 39 [OS=Homo sapiens] | 12 | RBM39 |
| High | P31949 | Protein S100-A11 [OS=Homo sapiens] | 44 | S100A11 |
| High | Q9NZN4 | EH domain-containing protein 2 [OS=Homo sapiens] | 14 | EHD2 |
| High | P23381 | Tryptophan--tRNA ligase, cytoplasmic [OS=Homo sapiens] | 8 | WARS1 |
| High | Q16643 | Drebrin [OS=Homo sapiens] | 13 | DBN1 |
| High | Q14651 | Plastin-1 [OS=Homo sapiens] | 7 | PLS1 |
| High | P04183 | Thymidine kinase, cytosolic [OS=Homo sapiens] | 25 | TK1 |
| High | P33992 | DNA replication licensing factor MCM5 [OS=Homo sapiens] | 12 | MCM5 |
| High | Q5T4S7 | E3 ubiquitin-protein ligase UBR4 [OS=Homo sapiens] | 1 | UBR4 |
| High | P20674 | Cytochrome c oxidase subunit 5A, mitochondrial [OS=Homo sapiens] | 36 | COX5A |
| High | Q16630 | Cleavage and polyadenylation specificity factor subunit 6 [OS=Homo sapiens] | 5 | CPSF6 |
| High | P19367 | Hexokinase-1 [OS=Homo sapiens] | 5 | HK1 |
| High | P39748 | Flap endonuclease 1 [OS=Homo sapiens] | 17 | FEN1 |
| High | P61077 | Ubiquitin-conjugating enzyme E2 D3 [OS=Homo sapiens] | 24 | UBE2D3 |
| High | Q96RT1 | Erbin [OS=Homo sapiens] | 3 | ERBIN |
| High | O15144 | Actin-related protein 2/3 complex subunit 2 [OS=Homo sapiens] | 16 | ARPC2 |
| High | Q7Z4W1 | L-xylulose reductase [OS=Homo sapiens] | 14 | DCXR |
| High | P60228 | Eukaryotic translation initiation factor 3 subunit E [OS=Homo sapiens] | 11 | EIF3E |
| High | Q9NYU2 | UDP-glucose:glycoprotein glucosyltransferase 1 [OS=Homo sapiens] | 4 | UGGT1 |
| High | Q92598 | Heat shock protein 105 kDa [OS=Homo sapiens] | 8 | HSPH1 |
| High | P60866 | 40S ribosomal protein S20 [OS=Homo sapiens] | 29 | RPS20 |
| High | P17858 | ATP-dependent 6-phosphofructokinase, liver type [OS=Homo sapiens] | 7 | PFKL |
| High | Q9BUJ2 | Heterogeneous nuclear ribonucleoprotein U-like protein 1 [OS=Homo sapiens] | 9 | HNRNPUL1 |
| High | P05455 | Lupus La protein [OS=Homo sapiens] | 9 | SSB |
| High | P07858 | Cathepsin B [OS=Homo sapiens] | 19 | CTSB |
| High | P0DP23 | Calmodulin-1 [OS=Homo sapiens] | 22 | CALM1 |
| High | P54727 | UV excision repair protein RAD23 homolog B [OS=Homo sapiens] | 7 | RAD23B |
| High | O75694 | Nuclear pore complex protein Nup155 [OS=Homo sapiens] | 4 | NUP155 |
| High | O95865 | N(G),N(G)-dimethylarginine dimethylaminohydrolase 2 [OS=Homo sapiens] | 30 | DDAH2 |
| High | O00764 | Pyridoxal kinase [OS=Homo sapiens] | 12 | PDXK |
| High | P53618 | Coatomer subunit beta [OS=Homo sapiens] | 9 | COPB1 |
| High | P63173 | 60S ribosomal protein L38 [OS=Homo sapiens] | 54 | RPL38 |
| High | P35268 | 60S ribosomal protein L22 [OS=Homo sapiens] | 40 | RPL22 |
| High | Q15185 | Prostaglandin E synthase 3 [OS=Homo sapiens] | 23 | PTGES3 |
| High | P41091 | Eukaryotic translation initiation factor 2 subunit 3 [OS=Homo sapiens] | 14 | EIF2S3 |
| High | P63313 | Thymosin beta-10 [OS=Homo sapiens] | 32 | TMSB10 |
| High | Q8N684 | Cleavage and polyadenylation specificity factor subunit 7 [OS=Homo sapiens] | 8 | CPSF7 |
| High | Q9H299 | SH3 domain-binding glutamic acid-rich-like protein 3 [OS=Homo sapiens] | 46 | SH3BGRL3 |
| High | P00441 | Superoxide dismutase [Cu-Zn] [OS=Homo sapiens] | 54 | SOD1 |
| High | O95831 | Apoptosis-inducing factor 1, mitochondrial [OS=Homo sapiens] | 6 | AIFM1 |
| High | P50502 | Hsc70-interacting protein [OS=Homo sapiens] | 16 | ST13 |
| High | P02768 | Albumin [OS=Homo sapiens] | 7 | ALB |
| High | P63000 | Ras-related C3 botulinum toxin substrate 1 [OS=Homo sapiens] | 27 | RAC1 |
| High | P51148 | Ras-related protein Rab-5C [OS=Homo sapiens] | 33 | RAB5C |
| High | Q07960 | Rho GTPase-activating protein 1 [OS=Homo sapiens] | 20 | ARHGAP1 |
| High | P40429 | 60S ribosomal protein L13a [OS=Homo sapiens] | 33 | RPL13A |
| High | P24844 | Myosin regulatory light polypeptide 9 [OS=Homo sapiens] | 39 | MYL9 |
| High | P61026 | Ras-related protein Rab-10 [OS=Homo sapiens] | 20 | RAB10 |
| High | P13804 | Electron transfer flavoprotein subunit alpha, mitochondrial [OS=Homo sapiens] | 17 | ETFA |
| High | P61019 | Ras-related protein Rab-2A [OS=Homo sapiens] | 20 | RAB2A |
| High | Q13724 | Mannosyl-oligosaccharide glucosidase [OS=Homo sapiens] | 7 | MOGS |
| High | P21291 | Cysteine and glycine-rich protein 1 [OS=Homo sapiens] | 26 | CSRP1 |
| High | Q13151 | Heterogeneous nuclear ribonucleoprotein A0 [OS=Homo sapiens] | 11 | HNRNPA0 |
| High | P23588 | Eukaryotic translation initiation factor 4B [OS=Homo sapiens] | 13 | EIF4B |
| High | P61353 | 60S ribosomal protein L27 [OS=Homo sapiens] | 30 | RPL27 |
| High | Q6PIU2 | Neutral cholesterol ester hydrolase 1 [OS=Homo sapiens] | 14 | NCEH1 |
| High | P30566 | Adenylosuccinate lyase [OS=Homo sapiens] | 17 | ADSL |
| High | Q96TA1 | Protein Niban 2 [OS=Homo sapiens] | 7 | NIBAN2 |
| High | P47914 | 60S ribosomal protein L29 [OS=Homo sapiens] | 15 | RPL29 |
| High | P23193 | Transcription elongation factor A protein 1 [OS=Homo sapiens] | 9 | TCEA1 |
| High | O43242 | 26S proteasome non-ATPase regulatory subunit 3 [OS=Homo sapiens] | 9 | PSMD3 |
| High | P62244 | 40S ribosomal protein S15a [OS=Homo sapiens] | 34 | RPS15A |
| High | P45880 | Voltage-dependent anion-selective channel protein 2 [OS=Homo sapiens] | 24 | VDAC2 |
| High | Q16181 | Septin-7 [OS=Homo sapiens] | 13 | SEPTIN7 |
| High | P33316 | Deoxyuridine 5'-triphosphate nucleotidohydrolase, mitochondrial [OS=Homo sapiens] | 19 | DUT |
| High | Q9Y3D6 | Mitochondrial fission 1 protein [OS=Homo sapiens] | 18 | FIS1 |
| High | Q9NYL9 | Tropomodulin-3 [OS=Homo sapiens] | 25 | TMOD3 |
| High | P38606 | V-type proton ATPase catalytic subunit A [OS=Homo sapiens] | 13 | ATP6V1A |
| High | Q15293 | Reticulocalbin-1 [OS=Homo sapiens] | 13 | RCN1 |
| High | Q04760 | Lactoylglutathione lyase [OS=Homo sapiens] | 30 | GLO1 |
| High | Q02543 | 60S ribosomal protein L18a [OS=Homo sapiens] | 30 | RPL18A |
| High | P23526 | Adenosylhomocysteinase [OS=Homo sapiens] | 11 | AHCY |
| High | P06737 | Glycogen phosphorylase, liver form [OS=Homo sapiens] | 4 | PYGL |
| High | P99999 | Cytochrome c [OS=Homo sapiens] | 50 | CYCS |
| High | O15460 | Prolyl 4-hydroxylase subunit alpha-2 [OS=Homo sapiens] | 8 | P4HA2 |
| High | Q9Y3F4 | Serine-threonine kinase receptor-associated protein [OS=Homo sapiens] | 26 | STRAP |
| High | P09622 | Dihydrolipoyl dehydrogenase, mitochondrial [OS=Homo sapiens] | 18 | DLD |
| High | P07305 | Histone H1.0 [OS=Homo sapiens] | 17 | H1-0 |
| High | P63172 | Dynein light chain Tctex-type 1 [OS=Homo sapiens] | 35 | DYNLT1 |
| High | O95340 | Bifunctional 3'-phosphoadenosine 5'-phosphosulfate synthase 2 [OS=Homo sapiens] | 10 | PAPSS2 |
| High | P35244 | Replication protein A 14 kDa subunit [OS=Homo sapiens] | 33 | RPA3 |
| High | Q9NTK5 | Obg-like ATPase 1 [OS=Homo sapiens] | 4 | OLA1 |
| High | Q8IZ83 | Aldehyde dehydrogenase family 16 member A1 [OS=Homo sapiens] | 7 | ALDH16A1 |
| High | P67775 | Serine/threonine-protein phosphatase 2A catalytic subunit alpha isoform [OS=Homo sapiens] | 28 | PPP2CA |
| High | P14324 | Farnesyl pyrophosphate synthase [OS=Homo sapiens] | 15 | FDPS |
| High | Q9UBQ7 | Glyoxylate reductase/hydroxypyruvate reductase [OS=Homo sapiens] | 8 | GRHPR |
| High | Q96AQ6 | Pre-B-cell leukemia transcription factor-interacting protein 1 [OS=Homo sapiens] | 4 | PBXIP1 |
| High | O75131 | Copine-3 [OS=Homo sapiens] | 10 | CPNE3 |
| High | Q16891 | MICOS complex subunit MIC60 [OS=Homo sapiens] | 9 | IMMT |
| High | Q9H0C8 | Integrin-linked kinase-associated serine/threonine phosphatase 2C [OS=Homo sapiens] | 9 | ILKAP |
| High | O94979 | Protein transport protein Sec31A [OS=Homo sapiens] | 6 | SEC31A |
| High | Q92621 | Nuclear pore complex protein Nup205 [OS=Homo sapiens] | 1 | NUP205 |
| High | Q9NZ32 | Actin-related protein 10 [OS=Homo sapiens] | 11 | ACTR10 |
| High | Q9C005 | Protein dpy-30 homolog [OS=Homo sapiens] | 36 | DPY30 |
| High | Q7Z7H8 | 39S ribosomal protein L10, mitochondrial [OS=Homo sapiens] | 23 | MRPL10 |
| High | Q5JWF2 | Guanine nucleotide-binding protein G(s) subunit alpha isoforms XLas [OS=Homo sapiens] | 6 | GNAS |
| High | P62191 | 26S proteasome regulatory subunit 4 [OS=Homo sapiens] | 11 | PSMC1 |
| High | P21964 | Catechol O-methyltransferase [OS=Homo sapiens] | 30 | COMT |
| High | Q15417 | Calponin-3 [OS=Homo sapiens] | 15 | CNN3 |
| High | O76021 | Ribosomal L1 domain-containing protein 1 [OS=Homo sapiens] | 8 | RSL1D1 |
| High | Q01995 | Transgelin [OS=Homo sapiens] | 31 | TAGLN |
| High | P27694 | Replication protein A 70 kDa DNA-binding subunit [OS=Homo sapiens] | 21 | RPA1 |
| High | Q10713 | Mitochondrial-processing peptidase subunit alpha [OS=Homo sapiens] | 3 | PMPCA |
| High | P30040 | Endoplasmic reticulum resident protein 29 [OS=Homo sapiens] | 23 | ERP29 |
| High | O60488 | Long-chain-fatty-acid--CoA ligase 4 [OS=Homo sapiens] | 10 | ACSL4 |
| High | P16615 | Sarcoplasmic/endoplasmic reticulum calcium ATPase 2 [OS=Homo sapiens] | 3 | ATP2A2 |
| High | P04264 | Keratin, type II cytoskeletal 1 [OS=Homo sapiens] | 8 | KRT1 |
| High | Q9P0S9 | Transmembrane protein 14C [OS=Homo sapiens] | 26 | TMEM14C |
| High | P08574 | Cytochrome c1, heme protein, mitochondrial [OS=Homo sapiens] | 12 | CYC1 |
| High | Q14444 | Caprin-1 [OS=Homo sapiens] | 6 | CAPRIN1 |
| High | P62820 | Ras-related protein Rab-1A [OS=Homo sapiens] | 19 | RAB1A |
| High | P53999 | Activated RNA polymerase II transcriptional coactivator p15 [OS=Homo sapiens] | 19 | SUB1 |
| High | P07602 | Prosaposin [OS=Homo sapiens] | 5 | PSAP |
| High | P12955 | Xaa-Pro dipeptidase [OS=Homo sapiens] | 8 | PEPD |
| High | P62851 | 40S ribosomal protein S25 [OS=Homo sapiens] | 31 | RPS25 |
| High | Q08945 | FACT complex subunit SSRP1 [OS=Homo sapiens] | 7 | SSRP1 |
| High | Q15436 | Protein transport protein Sec23A [OS=Homo sapiens] | 12 | SEC23A |
| High | P50570 | Dynamin-2 [OS=Homo sapiens] | 5 | DNM2 |
| High | Q13185 | Chromobox protein homolog 3 [OS=Homo sapiens] | 30 | CBX3 |
| High | P08195 | 4F2 cell-surface antigen heavy chain [OS=Homo sapiens] | 7 | SLC3A2 |
| High | Q14318 | Peptidyl-prolyl cis-trans isomerase FKBP8 [OS=Homo sapiens] | 11 | FKBP8 |
| High | Q8TCG1 | Protein CIP2A [OS=Homo sapiens] | 3 | CIP2A |
| High | Q9Y295 | Developmentally-regulated GTP-binding protein 1 [OS=Homo sapiens] | 17 | DRG1 |
| High | P51665 | 26S proteasome non-ATPase regulatory subunit 7 [OS=Homo sapiens] | 14 | PSMD7 |
| High | Q13162 | Peroxiredoxin-4 [OS=Homo sapiens] | 8 | PRDX4 |
| High | Q9H3P2 | Negative elongation factor A [OS=Homo sapiens] | 5 | NELFA |
| High | P08240 | Signal recognition particle receptor subunit alpha [OS=Homo sapiens] | 8 | SRPRA |
| High | O00178 | GTP-binding protein 1 [OS=Homo sapiens] | 6 | GTPBP1 |
| High | P62995 | Transformer-2 protein homolog beta [OS=Homo sapiens] | 14 | TRA2B |
| High | P49720 | Proteasome subunit beta type-3 [OS=Homo sapiens] | 17 | PSMB3 |
| High | Q8NBT2 | Kinetochore protein Spc24 [OS=Homo sapiens] | 9 | SPC24 |
| High | Q9BQ39 | ATP-dependent RNA helicase DDX50 [OS=Homo sapiens] | 6 | DDX50 |
| High | O15260 | Surfeit locus protein 4 [OS=Homo sapiens] | 11 | SURF4 |
| High | Q9UBF2 | Coatomer subunit gamma-2 [OS=Homo sapiens] | 6 | COPG2 |
| High | Q92820 | Gamma-glutamyl hydrolase [OS=Homo sapiens] | 17 | GGH |
| High | Q14847 | LIM and SH3 domain protein 1 [OS=Homo sapiens] | 12 | LASP1 |
| High | P55786 | Puromycin-sensitive aminopeptidase [OS=Homo sapiens] | 8 | NPEPPS |
| High | Q8N1G4 | Leucine-rich repeat-containing protein 47 [OS=Homo sapiens] | 13 | LRRC47 |
| High | O94826 | Mitochondrial import receptor subunit TOM70 [OS=Homo sapiens] | 3 | TOMM70 |
| High | Q99439 | Calponin-2 [OS=Homo sapiens] | 9 | CNN2 |
| High | P62857 | 40S ribosomal protein S28 [OS=Homo sapiens] | 41 | RPS28 |
| High | P30085 | UMP-CMP kinase [OS=Homo sapiens] | 19 | CMPK1 |
| High | Q99622 | Protein C10 [OS=Homo sapiens] | 29 | C12orf57 |
| High | P16989 | Y-box-binding protein 3 [OS=Homo sapiens] | 10 | YBX3 |
| High | P11586 | C-1-tetrahydrofolate synthase, cytoplasmic [OS=Homo sapiens] | 8 | MTHFD1 |
| High | P20290 | Transcription factor BTF3 [OS=Homo sapiens] | 24 | BTF3 |
| High | P28838 | Cytosol aminopeptidase [OS=Homo sapiens] | 11 | LAP3 |
| High | P55957 | BH3-interacting domain death agonist [OS=Homo sapiens] | 8 | BID |
| High | Q9P2J5 | Leucine--tRNA ligase, cytoplasmic [OS=Homo sapiens] | 5 | LARS1 |
| High | P83731 | 60S ribosomal protein L24 [OS=Homo sapiens] | 26 | RPL24 |
| High | P28066 | Proteasome subunit alpha type-5 [OS=Homo sapiens] | 24 | PSMA5 |
| High | Q9UKM9 | RNA-binding protein Raly [OS=Homo sapiens] | 16 | RALY |
| High | P35250 | Replication factor C subunit 2 [OS=Homo sapiens] | 19 | RFC2 |
| High | Q9BQE5 | Apolipoprotein L2 [OS=Homo sapiens] | 8 | APOL2 |
| High | P52815 | 39S ribosomal protein L12, mitochondrial [OS=Homo sapiens] | 13 | MRPL12 |
| High | P43246 | DNA mismatch repair protein Msh2 [OS=Homo sapiens] | 3 | MSH2 |
| High | P62841 | 40S ribosomal protein S15 [OS=Homo sapiens] | 24 | RPS15 |
| High | P40121 | Macrophage-capping protein [OS=Homo sapiens] | 19 | CAPG |
| High | P62491 | Ras-related protein Rab-11A [OS=Homo sapiens] | 25 | RAB11A |
| High | P46778 | 60S ribosomal protein L21 [OS=Homo sapiens] | 18 | RPL21 |
| High | Q9NVI7 | ATPase family AAA domain-containing protein 3A [OS=Homo sapiens] | 8 | ATAD3A |
| High | P11279 | Lysosome-associated membrane glycoprotein 1 [OS=Homo sapiens] | 6 | LAMP1 |
| High | Q13409 | Cytoplasmic dynein 1 intermediate chain 2 [OS=Homo sapiens] | 8 | DYNC1I2 |
| High | P55263 | Adenosine kinase [OS=Homo sapiens] | 13 | ADK |
| High | Q15717 | ELAV-like protein 1 [OS=Homo sapiens] | 15 | ELAVL1 |
| High | P27708 | CAD protein [OS=Homo sapiens] | 3 | CAD |
| High | P17812 | CTP synthase 1 [OS=Homo sapiens] | 3 | CTPS1 |
| High | O95347 | Structural maintenance of chromosomes protein 2 [OS=Homo sapiens] | 4 | SMC2 |
| High | P30520 | Adenylosuccinate synthetase isozyme 2 [OS=Homo sapiens] | 8 | ADSS2 |
| High | P54725 | UV excision repair protein RAD23 homolog A [OS=Homo sapiens] | 8 | RAD23A |
| High | P09661 | U2 small nuclear ribonucleoprotein A' [OS=Homo sapiens] | 28 | SNRPA1 |
| High | P17655 | Calpain-2 catalytic subunit [OS=Homo sapiens] | 7 | CAPN2 |
| High | P62993 | Growth factor receptor-bound protein 2 [OS=Homo sapiens] | 13 | GRB2 |
| High | Q15651 | High mobility group nucleosome-binding domain-containing protein 3 [OS=Homo sapiens] | 15 | HMGN3 |
| High | Q9H0A0 | RNA cytidine acetyltransferase [OS=Homo sapiens] | 7 | NAT10 |
| High | P41252 | Isoleucine--tRNA ligase, cytoplasmic [OS=Homo sapiens] | 3 | IARS1 |
| High | P48163 | NADP-dependent malic enzyme [OS=Homo sapiens] | 10 | ME1 |
| High | P51114 | RNA-binding protein FXR1 [OS=Homo sapiens] | 5 | FXR1 |
| High | P61769 | Beta-2-microglobulin [OS=Homo sapiens] | 27 | B2M |
| High | Q15907 | Ras-related protein Rab-11B [OS=Homo sapiens] | 24 | RAB11B |
| High | Q15738 | Sterol-4-alpha-carboxylate 3-dehydrogenase, decarboxylating [OS=Homo sapiens] | 17 | NSDHL |
| High | P35611 | Alpha-adducin [OS=Homo sapiens] | 6 | ADD1 |
| High | P68402 | Platelet-activating factor acetylhydrolase IB subunit alpha2 [OS=Homo sapiens] | 12 | PAFAH1B2 |
| High | P49419 | Alpha-aminoadipic semialdehyde dehydrogenase [OS=Homo sapiens] | 14 | ALDH7A1 |
| High | P17096 | High mobility group protein HMG-I/HMG-Y [OS=Homo sapiens] | 23 | HMGA1 |
| High | Q09161 | Nuclear cap-binding protein subunit 1 [OS=Homo sapiens] | 7 | NCBP1 |
| High | Q14978 | Nucleolar and coiled-body phosphoprotein 1 [OS=Homo sapiens] | 5 | NOLC1 |
| High | P18858 | DNA ligase 1 [OS=Homo sapiens] | 6 | LIG1 |
| High | P62318 | Small nuclear ribonucleoprotein Sm D3 [OS=Homo sapiens] | 32 | SNRPD3 |
| High | Q96HY6 | DDRGK domain-containing protein 1 [OS=Homo sapiens] | 10 | DDRGK1 |
| High | Q02218 | 2-oxoglutarate dehydrogenase complex component E1 [OS=Homo sapiens] | 7 | OGDH |
| High | Q9H0S4 | Probable ATP-dependent RNA helicase DDX47 [OS=Homo sapiens] | 4 | DDX47 |
| High | O00560 | Syntenin-1 [OS=Homo sapiens] | 8 | SDCBP |
| High | Q14839 | Chromodomain-helicase-DNA-binding protein 4 [OS=Homo sapiens] | 2 | CHD4 |
| High | P25789 | Proteasome subunit alpha type-4 [OS=Homo sapiens] | 12 | PSMA4 |
| High | Q9Y2V2 | Calcium-regulated heat-stable protein 1 [OS=Homo sapiens] | 11 | CARHSP1 |
| High | Q9H3U1 | Protein unc-45 homolog A [OS=Homo sapiens] | 2 | UNC45A |
| High | P10644 | cAMP-dependent protein kinase type I-alpha regulatory subunit [OS=Homo sapiens] | 12 | PRKAR1A |
| High | P55884 | Eukaryotic translation initiation factor 3 subunit B [OS=Homo sapiens] | 10 | EIF3B |
| High | P09496 | Clathrin light chain A [OS=Homo sapiens] | 15 | CLTA |
| High | Q03426 | Mevalonate kinase [OS=Homo sapiens] | 6 | MVK |
| High | P11766 | Alcohol dehydrogenase class-3 [OS=Homo sapiens] | 29 | ADH5 |
| High | Q01581 | Hydroxymethylglutaryl-CoA synthase, cytoplasmic [OS=Homo sapiens] | 14 | HMGCS1 |
| High | O75347 | Tubulin-specific chaperone A [OS=Homo sapiens] | 27 | TBCA |
| High | Q8IZL8 | Proline-, glutamic acid- and leucine-rich protein 1 [OS=Homo sapiens] | 1 | PELP1 |
| High | O14579 | Coatomer subunit epsilon [OS=Homo sapiens] | 22 | COPE |
| High | P61006 | Ras-related protein Rab-8A [OS=Homo sapiens] | 17 | RAB8A |
| High | Q9C0B1 | Alpha-ketoglutarate-dependent dioxygenase FTO [OS=Homo sapiens] | 3 | FTO |
| High | P62195 | 26S proteasome regulatory subunit 8 [OS=Homo sapiens] | 8 | PSMC5 |
| High | Q15813 | Tubulin-specific chaperone E [OS=Homo sapiens] | 8 | TBCE |
| High | Q02952 | A-kinase anchor protein 12 [OS=Homo sapiens] | 3 | AKAP12 |
| High | P11310 | Medium-chain specific acyl-CoA dehydrogenase, mitochondrial [OS=Homo sapiens] | 7 | ACADM |
| High | P61254 | 60S ribosomal protein L26 [OS=Homo sapiens] | 23 | RPL26 |
| High | P12270 | Nucleoprotein TPR [OS=Homo sapiens] | 1 | TPR |
| High | P62910 | 60S ribosomal protein L32 [OS=Homo sapiens] | 20 | RPL32 |
| High | Q9UHB9 | Signal recognition particle subunit SRP68 [OS=Homo sapiens] | 9 | SRP68 |
| High | Q9BSD7 | Cancer-related nucleoside-triphosphatase [OS=Homo sapiens] | 11 | NTPCR |
| High | Q9UNM6 | 26S proteasome non-ATPase regulatory subunit 13 [OS=Homo sapiens] | 4 | PSMD13 |
| High | Q13561 | Dynactin subunit 2 [OS=Homo sapiens] | 14 | DCTN2 |
| High | Q9BZZ5 | Apoptosis inhibitor 5 [OS=Homo sapiens] | 3 | API5 |
| High | P00568 | Adenylate kinase isoenzyme 1 [OS=Homo sapiens] | 7 | AK1 |
| High | P25786 | Proteasome subunit alpha type-1 [OS=Homo sapiens] | 17 | PSMA1 |
| High | P54136 | Arginine--tRNA ligase, cytoplasmic [OS=Homo sapiens] | 7 | RARS1 |
| High | Q12765 | Secernin-1 [OS=Homo sapiens] | 7 | SCRN1 |
| High | P20073 | Annexin A7 [OS=Homo sapiens] | 11 | ANXA7 |
| High | P09960 | Leukotriene A-4 hydrolase [OS=Homo sapiens] | 4 | LTA4H |
| High | P15586 | N-acetylglucosamine-6-sulfatase [OS=Homo sapiens] | 8 | GNS |
| High | Q9P0M6 | Core histone macro-H2A.2 [OS=Homo sapiens] | 15 | MACROH2A2 |
| High | Q13435 | Splicing factor 3B subunit 2 [OS=Homo sapiens] | 4 | SF3B2 |
| High | Q14137 | Ribosome biogenesis protein BOP1 [OS=Homo sapiens] | 5 | BOP1 |
| High | O14818 | Proteasome subunit alpha type-7 [OS=Homo sapiens] | 25 | PSMA7 |
| High | O43776 | Asparagine--tRNA ligase, cytoplasmic [OS=Homo sapiens] | 7 | NARS1 |
| High | Q92504 | Zinc transporter SLC39A7 [OS=Homo sapiens] | 9 | SLC39A7 |
| High | P52788 | Spermine synthase [OS=Homo sapiens] | 16 | SMS |
| High | P22087 | rRNA 2'-O-methyltransferase fibrillarin [OS=Homo sapiens] | 13 | FBL |
| High | Q9UMS4 | Pre-mRNA-processing factor 19 [OS=Homo sapiens] | 13 | PRPF19 |
| High | Q01970 | 1-phosphatidylinositol 4,5-bisphosphate phosphodiesterase beta-3 [OS=Homo sapiens] | 3 | PLCB3 |
| High | P62873 | Guanine nucleotide-binding protein G(I)/G(S)/G(T) subunit beta-1 [OS=Homo sapiens] | 12 | GNB1 |
| High | P26639 | Threonine--tRNA ligase 1, cytoplasmic [OS=Homo sapiens] | 8 | TARS1 |
| High | P35606 | Coatomer subunit beta' [OS=Homo sapiens] | 8 | COPB2 |
| High | O95819 | Mitogen-activated protein kinase kinase kinase kinase 4 [OS=Homo sapiens] | 1 | MAP4K4 |
| High | P35658 | Nuclear pore complex protein Nup214 [OS=Homo sapiens] | 1 | NUP214 |
| High | P56192 | Methionine--tRNA ligase, cytoplasmic [OS=Homo sapiens] | 7 | MARS1 |
| High | Q96EP5 | DAZ-associated protein 1 [OS=Homo sapiens] | 6 | DAZAP1 |
| High | P07919 | Cytochrome b-c1 complex subunit 6, mitochondrial [OS=Homo sapiens] | 20 | UQCRH |
| High | P10619 | Lysosomal protective protein [OS=Homo sapiens] | 5 | CTSA |
| High | Q15435 | Protein phosphatase 1 regulatory subunit 7 [OS=Homo sapiens] | 8 | PPP1R7 |
| High | Q9Y285 | Phenylalanine--tRNA ligase alpha subunit [OS=Homo sapiens] | 3 | FARSA |
| High | P31040 | Succinate dehydrogenase [ubiquinone] flavoprotein subunit, mitochondrial [OS=Homo sapiens] | 2 | SDHA |
| High | Q16543 | Hsp90 co-chaperone Cdc37 [OS=Homo sapiens] | 8 | CDC37 |
| High | P49207 | 60S ribosomal protein L34 [OS=Homo sapiens] | 30 | RPL34 |
| High | Q9UBN7 | Histone deacetylase 6 [OS=Homo sapiens] | 6 | HDAC6 |
| High | O95837 | Guanine nucleotide-binding protein subunit alpha-14 [OS=Homo sapiens] | 6 | GNA14 |
| High | Q27J81 | Inverted formin-2 [OS=Homo sapiens] | 4 | INF2 |
| High | P35222 | Catenin beta-1 [OS=Homo sapiens] | 3 | CTNNB1 |
| High | Q92626 | Peroxidasin homolog [OS=Homo sapiens] | 2 | PXDN |
| High | Q8NC51 | Plasminogen activator inhibitor 1 RNA-binding protein [OS=Homo sapiens] | 7 | SERBP1 |
| High | P35754 | Glutaredoxin-1 [OS=Homo sapiens] | 38 | GLRX |
| High | P62913 | 60S ribosomal protein L11 [OS=Homo sapiens] | 20 | RPL11 |
| High | P00492 | Hypoxanthine-guanine phosphoribosyltransferase [OS=Homo sapiens] | 29 | HPRT1 |
| High | Q9UN86 | Ras GTPase-activating protein-binding protein 2 [OS=Homo sapiens] | 7 | G3BP2 |
| High | Q06323 | Proteasome activator complex subunit 1 [OS=Homo sapiens] | 18 | PSME1 |
| High | P52926 | High mobility group protein HMGI-C [OS=Homo sapiens] | 21 | HMGA2 |
| High | O75494 | Serine/arginine-rich splicing factor 10 [OS=Homo sapiens] | 11 | SRSF10 |
| High | P46977 | Dolichyl-diphosphooligosaccharide--protein glycosyltransferase subunit STT3A [OS=Homo sapiens] | 8 | STT3A |
| High | Q14980 | Nuclear mitotic apparatus protein 1 [OS=Homo sapiens] | 3 | NUMA1 |
| High | Q9UBE0 | SUMO-activating enzyme subunit 1 [OS=Homo sapiens] | 12 | SAE1 |
| High | P51659 | Peroxisomal multifunctional enzyme type 2 [OS=Homo sapiens] | 6 | HSD17B4 |
| High | Q7L2H7 | Eukaryotic translation initiation factor 3 subunit M [OS=Homo sapiens] | 17 | EIF3M |
| High | O60784 | Target of Myb1 membrane trafficking protein [OS=Homo sapiens] | 6 | TOM1 |
| High | P27105 | Stomatin [OS=Homo sapiens] | 19 | STOM |
| High | O15355 | Protein phosphatase 1G [OS=Homo sapiens] | 11 | PPM1G |
| High | Q9P2R3 | Rabankyrin-5 [OS=Homo sapiens] | 2 | ANKFY1 |
| High | Q9Y266 | Nuclear migration protein nudC [OS=Homo sapiens] | 10 | NUDC |
| High | P07686 | Beta-hexosaminidase subunit beta [OS=Homo sapiens] | 9 | HEXB |
| High | P57764 | Gasdermin-D [OS=Homo sapiens] | 10 | GSDMD |
| High | O00233 | 26S proteasome non-ATPase regulatory subunit 9 [OS=Homo sapiens] | 11 | PSMD9 |
| High | O14662 | Syntaxin-16 [OS=Homo sapiens] | 6 | STX16 |
| High | P29353 | SHC-transforming protein 1 [OS=Homo sapiens] | 4 | SHC1 |
| High | P51570 | Galactokinase [OS=Homo sapiens] | 16 | GALK1 |
| High | P61221 | ATP-binding cassette sub-family E member 1 [OS=Homo sapiens] | 9 | ABCE1 |
| High | P42285 | Exosome RNA helicase MTR4 [OS=Homo sapiens] | 5 | MTREX |
| High | Q8TEX9 | Importin-4 [OS=Homo sapiens] | 5 | IPO4 |
| High | Q6EMK4 | Vasorin [OS=Homo sapiens] | 4 | VASN |
| High | P13473 | Lysosome-associated membrane glycoprotein 2 [OS=Homo sapiens] | 7 | LAMP2 |
| High | Q8NDT2 | Putative RNA-binding protein 15B [OS=Homo sapiens] | 2 | RBM15B |
| High | P21281 | V-type proton ATPase subunit B, brain isoform [OS=Homo sapiens] | 10 | ATP6V1B2 |
| High | Q9Y5L0 | Transportin-3 [OS=Homo sapiens] | 4 | TNPO3 |
| High | P23258 | Tubulin gamma-1 chain [OS=Homo sapiens] | 10 | TUBG1 |
| High | Q9GZS3 | SKI8 subunit of superkiller complex protein [OS=Homo sapiens] | 7 | SKIC8 |
| High | O43795 | Unconventional myosin-Ib [OS=Homo sapiens] | 3 | MYO1B |
| High | P16070 | CD44 antigen [OS=Homo sapiens] | 3 | CD44 |
| High | Q9ULC4 | Malignant T-cell-amplified sequence 1 [OS=Homo sapiens] | 20 | MCTS1 |
| High | Q6P2E9 | Enhancer of mRNA-decapping protein 4 [OS=Homo sapiens] | 2 | EDC4 |
| High | O00231 | 26S proteasome non-ATPase regulatory subunit 11 [OS=Homo sapiens] | 6 | PSMD11 |
| High | O14920 | Inhibitor of nuclear factor kappa-B kinase subunit beta [OS=Homo sapiens] | 2 | IKBKB |
| High | P62633 | CCHC-type zinc finger nucleic acid binding protein [OS=Homo sapiens] | 17 | CNBP |
| High | Q5JTH9 | RRP12-like protein [OS=Homo sapiens] | 3 | RRP12 |
| High | Q16836 | Hydroxyacyl-coenzyme A dehydrogenase, mitochondrial [OS=Homo sapiens] | 10 | HADH |
| High | P19784 | Casein kinase II subunit alpha' [OS=Homo sapiens] | 5 | CSNK2A2 |
| High | Q13404 | Ubiquitin-conjugating enzyme E2 variant 1 [OS=Homo sapiens] | 18 | UBE2V1 |
| High | Q13126 | S-methyl-5'-thioadenosine phosphorylase [OS=Homo sapiens] | 14 | MTAP |
| High | P23610 | 40-kDa huntingtin-associated protein [OS=Homo sapiens] | 8 | F8A1; F8A2; F8A3 |
| High | P10768 | S-formylglutathione hydrolase [OS=Homo sapiens] | 22 | ESD |
| High | Q9H1E3 | Nuclear ubiquitous casein and cyclin-dependent kinase substrate 1 [OS=Homo sapiens] | 15 | NUCKS1 |
| High | O14617 | AP-3 complex subunit delta-1 [OS=Homo sapiens] | 2 | AP3D1 |
| High | P04899 | Guanine nucleotide-binding protein G(i) subunit alpha-2 [OS=Homo sapiens] | 10 | GNAI2 |
| High | Q99733 | Nucleosome assembly protein 1-like 4 [OS=Homo sapiens] | 14 | NAP1L4 |
| High | Q96FW1 | Ubiquitin thioesterase OTUB1 [OS=Homo sapiens] | 7 | OTUB1 |
| High | Q9Y263 | Phospholipase A-2-activating protein [OS=Homo sapiens] | 2 | PLAA |
| High | Q9Y230 | RuvB-like 2 [OS=Homo sapiens] | 9 | RUVBL2 |
| High | P62861 | FAU ubiquitin-like and ribosomal protein S30 [OS=Homo sapiens] | 8 | FAU |
| High | P00367 | Glutamate dehydrogenase 1, mitochondrial [OS=Homo sapiens] | 13 | GLUD1 |
| High | P62333 | 26S proteasome regulatory subunit 10B [OS=Homo sapiens] | 12 | PSMC6 |
| High | Q9Y4R8 | Telomere length regulation protein TEL2 homolog [OS=Homo sapiens] | 6 | TELO2 |
| High | O00232 | 26S proteasome non-ATPase regulatory subunit 12 [OS=Homo sapiens] | 3 | PSMD12 |
| High | P42356 | Phosphatidylinositol 4-kinase alpha [OS=Homo sapiens] | 2 | PI4KA |
| High | Q9NVI1 | Fanconi anemia group I protein [OS=Homo sapiens] | 1 | FANCI |
| High | P98179 | RNA-binding protein 3 [OS=Homo sapiens] | 20 | RBM3 |
| High | Q9BPX3 | Condensin complex subunit 3 [OS=Homo sapiens] | 3 | NCAPG |
| High | Q02241 | Kinesin-like protein KIF23 [OS=Homo sapiens] | 6 | KIF23 |
| High | P23921 | Ribonucleoside-diphosphate reductase large subunit [OS=Homo sapiens] | 5 | RRM1 |
| High | O95295 | SNARE-associated protein Snapin [OS=Homo sapiens] | 19 | SNAPIN |
| High | Q14157 | Ubiquitin-associated protein 2-like [OS=Homo sapiens] | 5 | UBAP2L |
| High | O75937 | DnaJ homolog subfamily C member 8 [OS=Homo sapiens] | 20 | DNAJC8 |
| High | Q8N726 | Tumor suppressor ARF [OS=Homo sapiens] | 14 | CDKN2A |
| High | P31350 | Ribonucleoside-diphosphate reductase subunit M2 [OS=Homo sapiens] | 6 | RRM2 |
| High | Q6NUK1 | Calcium-binding mitochondrial carrier protein SCaMC-1 [OS=Homo sapiens] | 6 | SLC25A24 |
| High | Q9BRA2 | Thioredoxin domain-containing protein 17 [OS=Homo sapiens] | 27 | TXNDC17 |
| High | Q99598 | Translin-associated protein X [OS=Homo sapiens] | 7 | TSNAX |
| High | Q9NXF1 | Testis-expressed protein 10 [OS=Homo sapiens] | 3 | TEX10 |
| High | Q9H910 | Jupiter microtubule associated homolog 2 [OS=Homo sapiens] | 15 | JPT2 |
| High | P13073 | Cytochrome c oxidase subunit 4 isoform 1, mitochondrial [OS=Homo sapiens] | 12 | COX4I1 |
| High | P62899 | 60S ribosomal protein L31 [OS=Homo sapiens] | 14 | RPL31 |
| High | Q9UH99 | SUN domain-containing protein 2 [OS=Homo sapiens] | 7 | SUN2 |
| High | O60936 | Nucleolar protein 3 [OS=Homo sapiens] | 9 | NOL3 |
| High | P35998 | 26S proteasome regulatory subunit 7 [OS=Homo sapiens] | 10 | PSMC2 |
| High | P28072 | Proteasome subunit beta type-6 [OS=Homo sapiens] | 21 | PSMB6 |
| High | Q13620 | Cullin-4B [OS=Homo sapiens] | 4 | CUL4B |
| High | O00567 | Nucleolar protein 56 [OS=Homo sapiens] | 8 | NOP56 |
| High | P14868 | Aspartate--tRNA ligase, cytoplasmic [OS=Homo sapiens] | 6 | DARS1 |
| High | Q13547 | Histone deacetylase 1 [OS=Homo sapiens] | 7 | HDAC1 |
| High | P05114 | Non-histone chromosomal protein HMG-14 [OS=Homo sapiens] | 28 | HMGN1 |
| High | Q9BV20 | Methylthioribose-1-phosphate isomerase [OS=Homo sapiens] | 14 | MRI1 |
| High | P54578 | Ubiquitin carboxyl-terminal hydrolase 14 [OS=Homo sapiens] | 9 | USP14 |
| High | P12109 | Collagen alpha-1(VI) chain [OS=Homo sapiens] | 2 | COL6A1 |
| High | P14209 | CD99 antigen [OS=Homo sapiens] | 19 | CD99 |
| High | P48637 | Glutathione synthetase [OS=Homo sapiens] | 8 | GSS |
| High | P48556 | 26S proteasome non-ATPase regulatory subunit 8 [OS=Homo sapiens] | 7 | PSMD8 |
| High | Q9UI26 | Importin-11 [OS=Homo sapiens] | 8 | IPO11 |
| High | Q9Y6B6 | GTP-binding protein SAR1b [OS=Homo sapiens] | 16 | SAR1B |
| High | Q15437 | Protein transport protein Sec23B [OS=Homo sapiens] | 5 | SEC23B |
| High | Q9NTJ3 | Structural maintenance of chromosomes protein 4 [OS=Homo sapiens] | 4 | SMC4 |
| High | Q96AB3 | Isochorismatase domain-containing protein 2 [OS=Homo sapiens] | 24 | ISOC2 |
| High | Q8IWE2 | Protein NOXP20 [OS=Homo sapiens] | 6 | FAM114A1 |
| High | P52306 | Rap1 GTPase-GDP dissociation stimulator 1 [OS=Homo sapiens] | 3 | RAP1GDS1 |
| High | Q14257 | Reticulocalbin-2 [OS=Homo sapiens] | 13 | RCN2 |
| High | P62750 | 60S ribosomal protein L23a [OS=Homo sapiens] | 24 | RPL23A |
| High | A6NHR9 | Structural maintenance of chromosomes flexible hinge domain-containing protein 1 [OS=Homo sapiens] | 2 | SMCHD1 |
| High | P61086 | Ubiquitin-conjugating enzyme E2 K [OS=Homo sapiens] | 19 | UBE2K |
| High | P53992 | Protein transport protein Sec24C [OS=Homo sapiens] | 4 | SEC24C |
| High | Q13347 | Eukaryotic translation initiation factor 3 subunit I [OS=Homo sapiens] | 16 | EIF3I |
| High | P62854 | 40S ribosomal protein S26 [OS=Homo sapiens] | 21 | RPS26 |
| High | P18583 | Protein SON [OS=Homo sapiens] | 1 | SON |
| High | Q8IYB3 | Serine/arginine repetitive matrix protein 1 [OS=Homo sapiens] | 4 | SRRM1 |
| High | O75947 | ATP synthase subunit d, mitochondrial [OS=Homo sapiens] | 20 | ATP5PD |
| High | P07384 | Calpain-1 catalytic subunit [OS=Homo sapiens] | 7 | CAPN1 |
| High | P61289 | Proteasome activator complex subunit 3 [OS=Homo sapiens] | 12 | PSME3 |
| High | Q96FJ2 | Dynein light chain 2, cytoplasmic [OS=Homo sapiens] | 12 | DYNLL2 |
| High | O95881 | Thioredoxin domain-containing protein 12 [OS=Homo sapiens] | 14 | TXNDC12 |
| High | P59998 | Actin-related protein 2/3 complex subunit 4 [OS=Homo sapiens] | 18 | ARPC4 |
| High | P05198 | Eukaryotic translation initiation factor 2 subunit 1 [OS=Homo sapiens] | 11 | EIF2S1 |
| High | O75396 | Vesicle-trafficking protein SEC22b [OS=Homo sapiens] | 12 | SEC22B |
| High | Q6DD88 | Atlastin-3 [OS=Homo sapiens] | 9 | ATL3 |
| High | Q15050 | Ribosome biogenesis regulatory protein homolog [OS=Homo sapiens] | 7 | RRS1 |
| High | P55265 | Double-stranded RNA-specific adenosine deaminase [OS=Homo sapiens] | 3 | ADAR |
| High | Q9UHX1 | Poly(U)-binding-splicing factor PUF60 [OS=Homo sapiens] | 7 | PUF60 |
| High | P28482 | Mitogen-activated protein kinase 1 [OS=Homo sapiens] | 13 | MAPK1 |
| High | P46779 | 60S ribosomal protein L28 [OS=Homo sapiens] | 15 | RPL28 |
| High | P43490 | Nicotinamide phosphoribosyltransferase [OS=Homo sapiens] | 7 | NAMPT |
| High | Q9UBT2 | SUMO-activating enzyme subunit 2 [OS=Homo sapiens] | 10 | UBA2 |
| High | Q6XQN6 | Nicotinate phosphoribosyltransferase [OS=Homo sapiens] | 4 | NAPRT |
| High | Q6YHK3 | CD109 antigen [OS=Homo sapiens] | 2 | CD109 |
| High | P35221 | Catenin alpha-1 [OS=Homo sapiens] | 6 | CTNNA1 |
| High | Q8IY67 | Ribonucleoprotein PTB-binding 1 [OS=Homo sapiens] | 6 | RAVER1 |
| High | P19387 | DNA-directed RNA polymerase II subunit RPB3 [OS=Homo sapiens] | 7 | POLR2C |
| High | Q14676 | Mediator of DNA damage checkpoint protein 1 [OS=Homo sapiens] | 1 | MDC1 |
| High | Q13363 | C-terminal-binding protein 1 [OS=Homo sapiens] | 8 | CTBP1 |
| High | P13667 | Protein disulfide-isomerase A4 [OS=Homo sapiens] | 6 | PDIA4 |
| High | P35270 | Sepiapterin reductase [OS=Homo sapiens] | 8 | SPR |
| High | P51571 | Translocon-associated protein subunit delta [OS=Homo sapiens] | 17 | SSR4 |
| High | Q12849 | G-rich sequence factor 1 [OS=Homo sapiens] | 5 | GRSF1 |
| High | P49750 | YLP motif-containing protein 1 [OS=Homo sapiens] | 1 | YLPM1 |
| High | Q14919 | Dr1-associated corepressor [OS=Homo sapiens] | 6 | DRAP1 |
| High | O95816 | BAG family molecular chaperone regulator 2 [OS=Homo sapiens] | 13 | BAG2 |
| High | O15371 | Eukaryotic translation initiation factor 3 subunit D [OS=Homo sapiens] | 4 | EIF3D |
| High | Q9Y5B9 | FACT complex subunit SPT16 [OS=Homo sapiens] | 5 | SUPT16H |
| High | Q9Y277 | Voltage-dependent anion-selective channel protein 3 [OS=Homo sapiens] | 11 | VDAC3 |
| High | Q9NTI5 | Sister chromatid cohesion protein PDS5 homolog B [OS=Homo sapiens] | 3 | PDS5B |
| High | O15427 | Monocarboxylate transporter 4 [OS=Homo sapiens] | 5 | SLC16A3 |
| High | Q96HQ2 | CDKN2AIP N-terminal-like protein [OS=Homo sapiens] | 16 | CDKN2AIPNL |
| High | Q9NQC3 | Reticulon-4 [OS=Homo sapiens] | 4 | RTN4 |
| High | Q14694 | Ubiquitin carboxyl-terminal hydrolase 10 [OS=Homo sapiens] | 6 | USP10 |
| High | Q6PL18 | ATPase family AAA domain-containing protein 2 [OS=Homo sapiens] | 2 | ATAD2 |
| High | P78559 | Microtubule-associated protein 1A [OS=Homo sapiens] | 1 | MAP1A |
| High | P36543 | V-type proton ATPase subunit E 1 [OS=Homo sapiens] | 15 | ATP6V1E1 |
| High | Q16666 | Gamma-interferon-inducible protein 16 [OS=Homo sapiens] | 4 | IFI16 |
| High | P46060 | Ran GTPase-activating protein 1 [OS=Homo sapiens] | 6 | RANGAP1 |
| High | Q07157 | Tight junction protein ZO-1 [OS=Homo sapiens] | 2 | TJP1 |
| High | P19525 | Interferon-induced, double-stranded RNA-activated protein kinase [OS=Homo sapiens] | 4 | EIF2AK2 |
| High | O60518 | Ran-binding protein 6 [OS=Homo sapiens] | 3 | RANBP6 |
| High | Q9UPN3 | Microtubule-actin cross-linking factor 1, isoforms 1/2/3/4/5 [OS=Homo sapiens] | 1 | MACF1 |
| High | Q96F86 | Enhancer of mRNA-decapping protein 3 [OS=Homo sapiens] | 5 | EDC3 |
| High | Q15369 | Elongin-C [OS=Homo sapiens] | 12 | ELOC |
| High | P32322 | Pyrroline-5-carboxylate reductase 1, mitochondrial [OS=Homo sapiens] | 8 | PYCR1 |
| High | O43592 | Exportin-T [OS=Homo sapiens] | 3 | XPOT |
| High | Q15370 | Elongin-B [OS=Homo sapiens] | 28 | ELOB |
| High | Q96G03 | Phosphopentomutase [OS=Homo sapiens] | 4 | PGM2 |
| High | Q8N163 | Cell cycle and apoptosis regulator protein 2 [OS=Homo sapiens] | 4 | CCAR2 |
| High | P62316 | Small nuclear ribonucleoprotein Sm D2 [OS=Homo sapiens] | 24 | SNRPD2 |
| High | Q9UHD8 | Septin-9 [OS=Homo sapiens] | 5 | SEPTIN9 |
| High | P98082 | Disabled homolog 2 [OS=Homo sapiens] | 3 | DAB2 |
| High | P50995 | Annexin A11 [OS=Homo sapiens] | 5 | ANXA11 |
| High | P09936 | Ubiquitin carboxyl-terminal hydrolase isozyme L1 [OS=Homo sapiens] | 16 | UCHL1 |
| High | Q9BQ52 | Zinc phosphodiesterase ELAC protein 2 [OS=Homo sapiens] | 6 | ELAC2 |
| High | Q9BVP2 | Guanine nucleotide-binding protein-like 3 [OS=Homo sapiens] | 4 | GNL3 |
| High | Q92526 | T-complex protein 1 subunit zeta-2 [OS=Homo sapiens] | 5 | CCT6B |
| High | Q99873 | Protein arginine N-methyltransferase 1 [OS=Homo sapiens] | 4 | PRMT1 |
| High | Q8TCD5 | 5'(3')-deoxyribonucleotidase, cytosolic type [OS=Homo sapiens] | 9 | NT5C |
| High | P08243 | Asparagine synthetase [glutamine-hydrolyzing] [OS=Homo sapiens] | 5 | ASNS |
| High | P46063 | ATP-dependent DNA helicase Q1 [OS=Homo sapiens] | 5 | RECQL |
| High | Q9NSD9 | Phenylalanine--tRNA ligase beta subunit [OS=Homo sapiens] | 6 | FARSB |
| High | P58546 | Myotrophin [OS=Homo sapiens] | 14 | MTPN |
| High | Q9Y371 | Endophilin-B1 [OS=Homo sapiens] | 6 | SH3GLB1 |
| High | O00505 | Importin subunit alpha-4 [OS=Homo sapiens] | 11 | KPNA3 |
| High | Q92973 | Transportin-1 [OS=Homo sapiens] | 5 | TNPO1 |
| High | P20618 | Proteasome subunit beta type-1 [OS=Homo sapiens] | 11 | PSMB1 |
| High | O95486 | Protein transport protein Sec24A [OS=Homo sapiens] | 1 | SEC24A |
| High | Q9UNZ2 | NSFL1 cofactor p47 [OS=Homo sapiens] | 14 | NSFL1C |
| High | Q96AC1 | Fermitin family homolog 2 [OS=Homo sapiens] | 6 | FERMT2 |
| High | O75608 | Acyl-protein thioesterase 1 [OS=Homo sapiens] | 17 | LYPLA1 |
| High | P35659 | Protein DEK [OS=Homo sapiens] | 6 | DEK |
| High | Q9H8Y8 | Golgi reassembly-stacking protein 2 [OS=Homo sapiens] | 8 | GORASP2 |
| High | O60287 | Nucleolar pre-ribosomal-associated protein 1 [OS=Homo sapiens] | 2 | URB1 |
| High | Q3KQV9 | UDP-N-acetylhexosamine pyrophosphorylase-like protein 1 [OS=Homo sapiens] | 6 | UAP1L1 |
| High | Q8WXX5 | DnaJ homolog subfamily C member 9 [OS=Homo sapiens] | 8 | DNAJC9 |
| High | O95232 | Luc7-like protein 3 [OS=Homo sapiens] | 3 | LUC7L3 |
| High | Q9Y617 | Phosphoserine aminotransferase [OS=Homo sapiens] | 6 | PSAT1 |
| High | P55735 | Protein SEC13 homolog [OS=Homo sapiens] | 11 | SEC13 |
| High | Q92747 | Actin-related protein 2/3 complex subunit 1A [OS=Homo sapiens] | 9 | ARPC1A |
| High | P78344 | Eukaryotic translation initiation factor 4 gamma 2 [OS=Homo sapiens] | 1 | EIF4G2 |
| High | Q96FS4 | Signal-induced proliferation-associated protein 1 [OS=Homo sapiens] | 6 | SIPA1 |
| High | Q15046 | Lysine--tRNA ligase [OS=Homo sapiens] | 3 | KARS1 |
| High | Q15631 | Translin [OS=Homo sapiens] | 19 | TSN |
| High | Q9NVP1 | ATP-dependent RNA helicase DDX18 [OS=Homo sapiens] | 2 | DDX18 |
| High | Q16718 | NADH dehydrogenase [ubiquinone] 1 alpha subcomplex subunit 5 [OS=Homo sapiens] | 31 | NDUFA5 |
| High | P49773 | Adenosine 5'-monophosphoramidase HINT1 [OS=Homo sapiens] | 16 | HINT1 |
| High | Q13242 | Serine/arginine-rich splicing factor 9 [OS=Homo sapiens] | 10 | SRSF9 |
| High | Q9NPI6 | mRNA-decapping enzyme 1A [OS=Homo sapiens] | 6 | DCP1A |
| High | Q9NRG9 | Aladin [OS=Homo sapiens] | 3 | AAAS |
| High | O00479 | High mobility group nucleosome-binding domain-containing protein 4 [OS=Homo sapiens] | 17 | HMGN4 |
| High | Q96EY1 | DnaJ homolog subfamily A member 3, mitochondrial [OS=Homo sapiens] | 5 | DNAJA3 |
| High | Q9UL25 | Ras-related protein Rab-21 [OS=Homo sapiens] | 5 | RAB21 |
| High | O00425 | Insulin-like growth factor 2 mRNA-binding protein 3 [OS=Homo sapiens] | 5 | IGF2BP3 |
| High | Q15003 | Condensin complex subunit 2 [OS=Homo sapiens] | 6 | NCAPH |
| High | P49755 | Transmembrane emp24 domain-containing protein 10 [OS=Homo sapiens] | 9 | TMED10 |
| High | Q7Z4V5 | Hepatoma-derived growth factor-related protein 2 [OS=Homo sapiens] | 6 | HDGFL2 |
| High | Q8N1F7 | Nuclear pore complex protein Nup93 [OS=Homo sapiens] | 5 | NUP93 |
| High | O60568 | Multifunctional procollagen lysine hydroxylase and glycosyltransferase LH3 [OS=Homo sapiens] | 6 | PLOD3 |
| High | Q9NPF4 | tRNA N6-adenosine threonylcarbamoyltransferase [OS=Homo sapiens] | 8 | OSGEP |
| High | Q15758 | Neutral amino acid transporter B(0) [OS=Homo sapiens] | 4 | SLC1A5 |
| High | Q13492 | Phosphatidylinositol-binding clathrin assembly protein [OS=Homo sapiens] | 6 | PICALM |
| High | Q9BT78 | COP9 signalosome complex subunit 4 [OS=Homo sapiens] | 5 | COPS4 |
| High | O00170 | AH receptor-interacting protein [OS=Homo sapiens] | 8 | AIP |
| High | P35251 | Replication factor C subunit 1 [OS=Homo sapiens] | 2 | RFC1 |
| High | Q96CM8 | Medium-chain acyl-CoA ligase ACSF2, mitochondrial [OS=Homo sapiens] | 5 | ACSF2 |
| High | P49591 | Serine--tRNA ligase, cytoplasmic [OS=Homo sapiens] | 5 | SARS1 |
| High | O43823 | A-kinase anchor protein 8 [OS=Homo sapiens] | 3 | AKAP8 |
| High | Q86UP2 | Kinectin [OS=Homo sapiens] | 2 | KTN1 |
| High | P53007 | Tricarboxylate transport protein, mitochondrial [OS=Homo sapiens] | 15 | SLC25A1 |
| High | O60256 | Phosphoribosyl pyrophosphate synthase-associated protein 2 [OS=Homo sapiens] | 7 | PRPSAP2 |
| High | Q96FQ6 | Protein S100-A16 [OS=Homo sapiens] | 18 | S100A16 |
| High | O14578 | Citron Rho-interacting kinase [OS=Homo sapiens] | 1 | CIT |
| High | Q8NBP7 | Proprotein convertase subtilisin/kexin type 9 [OS=Homo sapiens] | 3 | PCSK9 |
| High | O75955 | Flotillin-1 [OS=Homo sapiens] | 7 | FLOT1 |
| High | P54619 | 5'-AMP-activated protein kinase subunit gamma-1 [OS=Homo sapiens] | 6 | PRKAG1 |
| High | Q8WX92 | Negative elongation factor B [OS=Homo sapiens] | 5 | NELFB |
| High | Q6ZS17 | Rho family-interacting cell polarization regulator 1 [OS=Homo sapiens] | 2 | RIPOR1 |
| High | O14737 | Programmed cell death protein 5 [OS=Homo sapiens] | 19 | PDCD5 |
| High | Q9BVI4 | Nucleolar complex protein 4 homolog [OS=Homo sapiens] | 8 | NOC4L |
| High | P16219 | Short-chain specific acyl-CoA dehydrogenase, mitochondrial [OS=Homo sapiens] | 6 | ACADS |
| High | P02792 | Ferritin light chain [OS=Homo sapiens] | 9 | FTL |
| High | O00264 | Membrane-associated progesterone receptor component 1 [OS=Homo sapiens] | 12 | PGRMC1 |
| High | O43684 | Mitotic checkpoint protein BUB3 [OS=Homo sapiens] | 12 | BUB3 |
| High | Q12797 | Aspartyl/asparaginyl beta-hydroxylase [OS=Homo sapiens] | 5 | ASPH |
| High | Q29RF7 | Sister chromatid cohesion protein PDS5 homolog A [OS=Homo sapiens] | 2 | PDS5A |
| High | Q9NPQ8 | Synembryn-A [OS=Homo sapiens] | 3 | RIC8A |
| High | Q96C90 | Protein phosphatase 1 regulatory subunit 14B [OS=Homo sapiens] | 15 | PPP1R14B |
| High | Q8NBX0 | Saccharopine dehydrogenase-like oxidoreductase [OS=Homo sapiens] | 7 | SCCPDH |
| High | Q96S52 | GPI transamidase component PIG-S [OS=Homo sapiens] | 4 | PIGS |
| High | Q15121 | Astrocytic phosphoprotein PEA-15 [OS=Homo sapiens] | 17 | PEA15 |
| High | P30419 | Glycylpeptide N-tetradecanoyltransferase 1 [OS=Homo sapiens] | 5 | NMT1 |
| High | P15927 | Replication protein A 32 kDa subunit [OS=Homo sapiens] | 9 | RPA2 |
| High | P61201 | COP9 signalosome complex subunit 2 [OS=Homo sapiens] | 6 | COPS2 |
| High | Q9HAV7 | GrpE protein homolog 1, mitochondrial [OS=Homo sapiens] | 9 | GRPEL1 |
| High | Q9H4A6 | Golgi phosphoprotein 3 [OS=Homo sapiens] | 13 | GOLPH3 |
| High | P26440 | Isovaleryl-CoA dehydrogenase, mitochondrial [OS=Homo sapiens] | 6 | IVD |
| High | Q9UHN6 | Cell surface hyaluronidase [OS=Homo sapiens] | 2 | CEMIP2 |
| High | Q99436 | Proteasome subunit beta type-7 [OS=Homo sapiens] | 10 | PSMB7 |
| High | O95379 | Tumor necrosis factor alpha-induced protein 8 [OS=Homo sapiens] | 10 | TNFAIP8 |
| High | Q5EB52 | Mesoderm-specific transcript homolog protein [OS=Homo sapiens] | 5 | MEST |
| High | Q05519 | Serine/arginine-rich splicing factor 11 [OS=Homo sapiens] | 3 | SRSF11 |
| High | P04179 | Superoxide dismutase [Mn], mitochondrial [OS=Homo sapiens] | 6 | SOD2 |
| High | Q9BS26 | Endoplasmic reticulum resident protein 44 [OS=Homo sapiens] | 9 | ERP44 |
| High | Q5RKV6 | Exosome complex component MTR3 [OS=Homo sapiens] | 13 | EXOSC6 |
| High | Q9Y276 | Mitochondrial chaperone BCS1 [OS=Homo sapiens] | 5 | BCS1L |
| High | P52732 | Kinesin-like protein KIF11 [OS=Homo sapiens] | 5 | KIF11 |
| High | O43432 | Eukaryotic translation initiation factor 4 gamma 3 [OS=Homo sapiens] | 1 | EIF4G3 |
| High | Q6P1J9 | Parafibromin [OS=Homo sapiens] | 4 | CDC73 |
| High | Q13443 | Disintegrin and metalloproteinase domain-containing protein 9 [OS=Homo sapiens] | 3 | ADAM9 |
| High | Q9BTW9 | Tubulin-specific chaperone D [OS=Homo sapiens] | 3 | TBCD |
| High | O75506 | Heat shock factor-binding protein 1 [OS=Homo sapiens] | 39 | HSBP1 |
| High | P36507 | Dual specificity mitogen-activated protein kinase kinase 2 [OS=Homo sapiens] | 6 | MAP2K2 |
| High | P63220 | 40S ribosomal protein S21 [OS=Homo sapiens] | 29 | RPS21 |
| High | Q9NVD7 | Alpha-parvin [OS=Homo sapiens] | 4 | PARVA |
| High | Q9NR09 | Baculoviral IAP repeat-containing protein 6 [OS=Homo sapiens] | 0 | BIRC6 |
| High | O14976 | Cyclin-G-associated kinase [OS=Homo sapiens] | 1 | GAK |
| High | Q5SRE5 | Nucleoporin NUP188 [OS=Homo sapiens] | 1 | NUP188 |
| High | O60841 | Eukaryotic translation initiation factor 5B [OS=Homo sapiens] | 3 | EIF5B |
| High | Q6P6C2 | RNA demethylase ALKBH5 [OS=Homo sapiens] | 7 | ALKBH5 |
| High | O43747 | AP-1 complex subunit gamma-1 [OS=Homo sapiens] | 4 | AP1G1 |
| High | P42677 | 40S ribosomal protein S27 [OS=Homo sapiens] | 25 | RPS27 |
| High | O14974 | Protein phosphatase 1 regulatory subunit 12A [OS=Homo sapiens] | 2 | PPP1R12A |
| High | P09486 | SPARC [OS=Homo sapiens] | 5 | SPARC |
| High | Q92769 | Histone deacetylase 2 [OS=Homo sapiens] | 7 | HDAC2 |
| High | Q96AT9 | Ribulose-phosphate 3-epimerase [OS=Homo sapiens] | 8 | RPE |
| High | Q6FI81 | Anamorsin [OS=Homo sapiens] | 9 | CIAPIN1 |
| High | Q969Q0 | 60S ribosomal protein L36a-like [OS=Homo sapiens] | 12 | RPL36AL |
| High | Q71SY5 | Mediator of RNA polymerase II transcription subunit 25 [OS=Homo sapiens] | 3 | MED25 |
| High | Q9Y6G9 | Cytoplasmic dynein 1 light intermediate chain 1 [OS=Homo sapiens] | 4 | DYNC1LI1 |
| High | P10155 | RNA-binding protein RO60 [OS=Homo sapiens] | 8 | RO60 |
| High | Q9Y496 | Kinesin-like protein KIF3A [OS=Homo sapiens] | 3 | KIF3A |
| High | P08397 | Porphobilinogen deaminase [OS=Homo sapiens] | 6 | HMBS |
| High | Q9BR76 | Coronin-1B [OS=Homo sapiens] | 8 | CORO1B |
| High | Q13247 | Serine/arginine-rich splicing factor 6 [OS=Homo sapiens] | 5 | SRSF6 |
| High | Q9UHI6 | Probable ATP-dependent RNA helicase DDX20 [OS=Homo sapiens] | 3 | DDX20 |
| High | Q9HB71 | Calcyclin-binding protein [OS=Homo sapiens] | 13 | CACYBP |
| High | P24941 | Cyclin-dependent kinase 2 [OS=Homo sapiens] | 7 | CDK2 |
| High | Q9Y2W2 | WW domain-binding protein 11 [OS=Homo sapiens] | 3 | WBP11 |
| High | Q9Y3I1 | F-box only protein 7 [OS=Homo sapiens] | 4 | FBXO7 |
| High | Q9BW60 | Elongation of very long chain fatty acids protein 1 [OS=Homo sapiens] | 5 | ELOVL1 |
| High | Q9NVA2 | Septin-11 [OS=Homo sapiens] | 7 | SEPTIN11 |
| High | P15121 | Aldo-keto reductase family 1 member B1 [OS=Homo sapiens] | 6 | AKR1B1 |
| High | O60869 | Endothelial differentiation-related factor 1 [OS=Homo sapiens] | 16 | EDF1 |
| High | P54886 | Delta-1-pyrroline-5-carboxylate synthase [OS=Homo sapiens] | 4 | ALDH18A1 |
| High | Q13564 | NEDD8-activating enzyme E1 regulatory subunit [OS=Homo sapiens] | 6 | NAE1 |
| High | P40938 | Replication factor C subunit 3 [OS=Homo sapiens] | 9 | RFC3 |
| High | Q13155 | Aminoacyl tRNA synthase complex-interacting multifunctional protein 2 [OS=Homo sapiens] | 20 | AIMP2 |
| High | Q8NEU8 | DCC-interacting protein 13-beta [OS=Homo sapiens] | 5 | APPL2 |
| High | Q8N1B4 | Vacuolar protein sorting-associated protein 52 homolog [OS=Homo sapiens] | 4 | VPS52 |
| High | Q5VW32 | BRO1 domain-containing protein BROX [OS=Homo sapiens] | 6 | BROX |
| High | P25788 | Proteasome subunit alpha type-3 [OS=Homo sapiens] | 10 | PSMA3 |
| High | Q9NVM6 | DnaJ homolog subfamily C member 17 [OS=Homo sapiens] | 7 | DNAJC17 |
| High | Q9NXR1 | Nuclear distribution protein nudE homolog 1 [OS=Homo sapiens] | 4 | NDE1 |
| High | P26358 | DNA (cytosine-5)-methyltransferase 1 [OS=Homo sapiens] | 2 | DNMT1 |
| High | P12081 | Histidine--tRNA ligase, cytoplasmic [OS=Homo sapiens] | 5 | HARS1 |
| High | P49593 | Protein phosphatase 1F [OS=Homo sapiens] | 6 | PPM1F |
| High | Q99471 | Prefoldin subunit 5 [OS=Homo sapiens] | 12 | PFDN5 |
| High | P30405 | Peptidyl-prolyl cis-trans isomerase F, mitochondrial [OS=Homo sapiens] | 21 | PPIF |
| High | Q9UI30 | Multifunctional methyltransferase subunit TRM112-like protein [OS=Homo sapiens] | 22 | TRMT112 |
| High | Q12792 | Twinfilin-1 [OS=Homo sapiens] | 5 | TWF1 |
| High | O00273 | DNA fragmentation factor subunit alpha [OS=Homo sapiens] | 15 | DFFA |
| High | O75251 | NADH dehydrogenase [ubiquinone] iron-sulfur protein 7, mitochondrial [OS=Homo sapiens] | 13 | NDUFS7 |
| High | Q14203 | Dynactin subunit 1 [OS=Homo sapiens] | 1 | DCTN1 |
| High | Q2M389 | WASH complex subunit 4 [OS=Homo sapiens] | 3 | WASHC4 |
| High | Q70UQ0 | Inhibitor of nuclear factor kappa-B kinase-interacting protein [OS=Homo sapiens] | 8 | IKBIP |
| High | P43304 | Glycerol-3-phosphate dehydrogenase, mitochondrial [OS=Homo sapiens] | 3 | GPD2 |
| High | Q13011 | Delta(3,5)-Delta(2,4)-dienoyl-CoA isomerase, mitochondrial [OS=Homo sapiens] | 8 | ECH1 |
| High | P55036 | 26S proteasome non-ATPase regulatory subunit 4 [OS=Homo sapiens] | 7 | PSMD4 |
| High | P62495 | Eukaryotic peptide chain release factor subunit 1 [OS=Homo sapiens] | 9 | ETF1 |
| High | Q06787 | Fragile X messenger ribonucleoprotein 1 [OS=Homo sapiens] | 2 | FMR1 |
| High | Q15459 | Splicing factor 3A subunit 1 [OS=Homo sapiens] | 3 | SF3A1 |
| High | P49721 | Proteasome subunit beta type-2 [OS=Homo sapiens] | 5 | PSMB2 |
| High | P0CG08 | Golgi pH regulator B [OS=Homo sapiens] | 6 | GPR89B |
| High | O14929 | Histone acetyltransferase type B catalytic subunit [OS=Homo sapiens] | 9 | HAT1 |
| High | Q9NQG5 | Regulation of nuclear pre-mRNA domain-containing protein 1B [OS=Homo sapiens] | 5 | RPRD1B |
| High | O75348 | V-type proton ATPase subunit G 1 [OS=Homo sapiens] | 22 | ATP6V1G1 |
| High | O60725 | Protein-S-isoprenylcysteine O-methyltransferase [OS=Homo sapiens] | 7 | ICMT |
| High | Q66LE6 | Serine/threonine-protein phosphatase 2A 55 kDa regulatory subunit B delta isoform [OS=Homo sapiens] | 3 | PPP2R2D |
| High | P55809 | Succinyl-CoA:3-ketoacid coenzyme A transferase 1, mitochondrial [OS=Homo sapiens] | 4 | OXCT1 |
| High | Q9BVC6 | Transmembrane protein 109 [OS=Homo sapiens] | 5 | TMEM109 |
| High | Q16186 | Proteasomal ubiquitin receptor ADRM1 [OS=Homo sapiens] | 4 | ADRM1 |
| High | Q9Y3C4 | EKC/KEOPS complex subunit TPRKB [OS=Homo sapiens] | 10 | TPRKB |
| High | P52701 | DNA mismatch repair protein Msh6 [OS=Homo sapiens] | 1 | MSH6 |
| High | P61923 | Coatomer subunit zeta-1 [OS=Homo sapiens] | 27 | COPZ1 |
| High | Q96PK6 | RNA-binding protein 14 [OS=Homo sapiens] | 4 | RBM14 |
| High | Q14011 | Cold-inducible RNA-binding protein [OS=Homo sapiens] | 12 | CIRBP |
| High | Q96EY7 | Pentatricopeptide repeat domain-containing protein 3, mitochondrial [OS=Homo sapiens] | 7 | PTCD3 |
| High | Q9UNF1 | Melanoma-associated antigen D2 [OS=Homo sapiens] | 2 | MAGED2 |
| High | O14773 | Tripeptidyl-peptidase 1 [OS=Homo sapiens] | 5 | TPP1 |
| High | P14550 | Aldo-keto reductase family 1 member A1 [OS=Homo sapiens] | 6 | AKR1A1 |
| High | P56134 | ATP synthase subunit f, mitochondrial [OS=Homo sapiens] | 14 | ATP5MF |
| High | O95433 | Activator of 90 kDa heat shock protein ATPase homolog 1 [OS=Homo sapiens] | 10 | AHSA1 |
| High | Q14195 | Dihydropyrimidinase-related protein 3 [OS=Homo sapiens] | 5 | DPYSL3 |
| High | P30046 | D-dopachrome decarboxylase [OS=Homo sapiens] | 19 | DDT |
| High | P11802 | Cyclin-dependent kinase 4 [OS=Homo sapiens] | 8 | CDK4 |
| High | O60502 | Protein O-GlcNAcase [OS=Homo sapiens] | 1 | OGA |
| High | P07954 | Fumarate hydratase, mitochondrial [OS=Homo sapiens] | 4 | FH |
| High | Q9Y3U8 | 60S ribosomal protein L36 [OS=Homo sapiens] | 10 | RPL36 |
| High | Q8NCF5 | NFATC2-interacting protein [OS=Homo sapiens] | 7 | NFATC2IP |
| High | Q03001 | Dystonin [OS=Homo sapiens] | 0 | DST |
| High | P00403 | Cytochrome c oxidase subunit 2 [OS=Homo sapiens] | 4 | MT-CO2 |
| High | Q1KMD3 | Heterogeneous nuclear ribonucleoprotein U-like protein 2 [OS=Homo sapiens] | 5 | HNRNPUL2 |
| High | P50402 | Emerin [OS=Homo sapiens] | 7 | EMD |
| High | P30049 | ATP synthase subunit delta, mitochondrial [OS=Homo sapiens] | 24 | ATP5F1D |
| High | Q13057 | Bifunctional coenzyme A synthase [OS=Homo sapiens] | 7 | COASY |
| High | P55854 | Small ubiquitin-related modifier 3 [OS=Homo sapiens] | 12 | SUMO3 |
| High | Q9UM00 | Calcium load-activated calcium channel [OS=Homo sapiens] | 6 | TMCO1 |
| High | Q14192 | Four and a half LIM domains protein 2 [OS=Homo sapiens] | 10 | FHL2 |
| High | Q9H008 | Phospholysine phosphohistidine inorganic pyrophosphate phosphatase [OS=Homo sapiens] | 8 | LHPP |
| High | Q8TF09 | Dynein light chain roadblock-type 2 [OS=Homo sapiens] | 13 | DYNLRB2 |
| High | Q15785 | Mitochondrial import receptor subunit TOM34 [OS=Homo sapiens] | 11 | TOMM34 |
| High | O75964 | ATP synthase subunit g, mitochondrial [OS=Homo sapiens] | 27 | ATP5MG |
| High | P49366 | Deoxyhypusine synthase [OS=Homo sapiens] | 5 | DHPS |
| High | O43143 | ATP-dependent RNA helicase DHX15 [OS=Homo sapiens] | 3 | DHX15 |
| High | Q8NBJ5 | Procollagen galactosyltransferase 1 [OS=Homo sapiens] | 4 | COLGALT1 |
| High | Q13895 | Bystin [OS=Homo sapiens] | 4 | BYSL |
| High | Q9ULZ3 | Apoptosis-associated speck-like protein containing a CARD [OS=Homo sapiens] | 9 | PYCARD |
| High | Q15257 | Serine/threonine-protein phosphatase 2A activator [OS=Homo sapiens] | 8 | PTPA |
| High | Q92922 | SWI/SNF complex subunit SMARCC1 [OS=Homo sapiens] | 2 | SMARCC1 |
| High | Q8TF05 | Serine/threonine-protein phosphatase 4 regulatory subunit 1 [OS=Homo sapiens] | 2 | PPP4R1 |
| High | Q96KG9 | N-terminal kinase-like protein [OS=Homo sapiens] | 5 | SCYL1 |
| High | Q9BW27 | Nuclear pore complex protein Nup85 [OS=Homo sapiens] | 2 | NUP85 |
| High | Q14008 | Cytoskeleton-associated protein 5 [OS=Homo sapiens] | 1 | CKAP5 |
| High | P52630 | Signal transducer and activator of transcription 2 [OS=Homo sapiens] | 1 | STAT2 |
| High | Q8WUX9 | Charged multivesicular body protein 7 [OS=Homo sapiens] | 4 | CHMP7 |
| High | P15170 | Eukaryotic peptide chain release factor GTP-binding subunit ERF3A [OS=Homo sapiens] | 7 | GSPT1 |
| High | Q96N66 | Lysophospholipid acyltransferase 7 [OS=Homo sapiens] | 5 | MBOAT7 |
| High | P80297 | Metallothionein-1X [OS=Homo sapiens] | 20 | MT1X |
| High | O75165 | DnaJ homolog subfamily C member 13 [OS=Homo sapiens] | 1 | DNAJC13 |
| High | P49354 | Protein farnesyltransferase/geranylgeranyltransferase type-1 subunit alpha [OS=Homo sapiens] | 5 | FNTA |
| High | P31483 | Cytotoxic granule associated RNA binding protein TIA1 [OS=Homo sapiens] | 5 | TIA1 |
| High | Q9UHV9 | Prefoldin subunit 2 [OS=Homo sapiens] | 9 | PFDN2 |
| High | P29317 | Ephrin type-A receptor 2 [OS=Homo sapiens] | 1 | EPHA2 |
| High | Q9UJ70 | N-acetyl-D-glucosamine kinase [OS=Homo sapiens] | 6 | NAGK |
| High | P28288 | ATP-binding cassette sub-family D member 3 [OS=Homo sapiens] | 2 | ABCD3 |
| High | Q92530 | Proteasome inhibitor PI31 subunit [OS=Homo sapiens] | 9 | PSMF1 |
| High | Q02978 | Mitochondrial 2-oxoglutarate/malate carrier protein [OS=Homo sapiens] | 6 | SLC25A11 |
| High | Q9H845 | Complex I assembly factor ACAD9, mitochondrial [OS=Homo sapiens] | 8 | ACAD9 |
| High | P35527 | Keratin, type I cytoskeletal 9 [OS=Homo sapiens] | 4 | KRT9 |
| High | Q53GS9 | U4/U6.U5 tri-snRNP-associated protein 2 [OS=Homo sapiens] | 3 | USP39 |
| High | Q7Z4H7 | HAUS augmin-like complex subunit 6 [OS=Homo sapiens] | 3 | HAUS6 |
| High | Q86U38 | Nucleolar protein 9 [OS=Homo sapiens] | 4 | NOP9 |
| High | Q13868 | Exosome complex component RRP4 [OS=Homo sapiens] | 9 | EXOSC2 |
| High | P46459 | Vesicle-fusing ATPase [OS=Homo sapiens] | 3 | NSF |
| High | Q9UNH7 | Sorting nexin-6 [OS=Homo sapiens] | 3 | SNX6 |
| High | Q9H9A6 | Leucine-rich repeat-containing protein 40 [OS=Homo sapiens] | 4 | LRRC40 |
| High | Q86Y56 | Dynein axonemal assembly factor 5 [OS=Homo sapiens] | 3 | DNAAF5 |
| High | P28799 | Progranulin [OS=Homo sapiens] | 5 | GRN |
| High | Q99460 | 26S proteasome non-ATPase regulatory subunit 1 [OS=Homo sapiens] | 1 | PSMD1 |
| High | Q9NQW6 | Anillin [OS=Homo sapiens] | 2 | ANLN |
| High | Q07812 | Apoptosis regulator BAX [OS=Homo sapiens] | 7 | BAX |
| High | P04216 | Thy-1 membrane glycoprotein [OS=Homo sapiens] | 9 | THY1 |
| High | P78347 | General transcription factor II-I [OS=Homo sapiens] | 4 | GTF2I |
| High | P51398 | 28S ribosomal protein S29, mitochondrial [OS=Homo sapiens] | 5 | DAP3 |
| High | O75821 | Eukaryotic translation initiation factor 3 subunit G [OS=Homo sapiens] | 5 | EIF3G |
| High | P24468 | COUP transcription factor 2 [OS=Homo sapiens] | 4 | NR2F2 |
| High | P43034 | Platelet-activating factor acetylhydrolase IB subunit beta [OS=Homo sapiens] | 3 | PAFAH1B1 |
| High | O75531 | Barrier-to-autointegration factor [OS=Homo sapiens] | 13 | BANF1 |
| High | Q7L576 | Cytoplasmic FMR1-interacting protein 1 [OS=Homo sapiens] | 2 | CYFIP1 |
| High | P36915 | Guanine nucleotide-binding protein-like 1 [OS=Homo sapiens] | 7 | GNL1 |
| High | Q92882 | Osteoclast-stimulating factor 1 [OS=Homo sapiens] | 6 | OSTF1 |
| High | Q8TBC4 | NEDD8-activating enzyme E1 catalytic subunit [OS=Homo sapiens] | 5 | UBA3 |
| High | Q9HC38 | Glyoxalase domain-containing protein 4 [OS=Homo sapiens] | 4 | GLOD4 |
| High | O95394 | Phosphoacetylglucosamine mutase [OS=Homo sapiens] | 10 | PGM3 |
| High | Q8IUR7 | Armadillo repeat-containing protein 8 [OS=Homo sapiens] | 3 | ARMC8 |
| High | Q15637 | Splicing factor 1 [OS=Homo sapiens] | 4 | SF1 |
| High | Q9H9Q4 | Non-homologous end-joining factor 1 [OS=Homo sapiens] | 7 | NHEJ1 |
| High | P62847 | 40S ribosomal protein S24 [OS=Homo sapiens] | 9 | RPS24 |
| High | O95777 | U6 snRNA-associated Sm-like protein LSm8 [OS=Homo sapiens] | 17 | LSM8 |
| High | P46379 | Large proline-rich protein BAG6 [OS=Homo sapiens] | 3 | BAG6 |
| High | Q6PKG0 | La-related protein 1 [OS=Homo sapiens] | 1 | LARP1 |
| High | Q9NRP0 | Oligosaccharyltransferase complex subunit OSTC [OS=Homo sapiens] | 8 | OSTC |
| High | Q9P258 | Protein RCC2 [OS=Homo sapiens] | 3 | RCC2 |
| High | Q02790 | Peptidyl-prolyl cis-trans isomerase FKBP4 [OS=Homo sapiens] | 4 | FKBP4 |
| High | P06454 | Prothymosin alpha [OS=Homo sapiens] | 9 | PTMA |
| High | Q96T37 | RNA-binding protein 15 [OS=Homo sapiens] | 2 | RBM15 |
| High | Q32P28 | Prolyl 3-hydroxylase 1 [OS=Homo sapiens] | 1 | P3H1 |
| High | Q14558 | Phosphoribosyl pyrophosphate synthase-associated protein 1 [OS=Homo sapiens] | 12 | PRPSAP1 |
| High | Q9UBW8 | COP9 signalosome complex subunit 7a [OS=Homo sapiens] | 11 | COPS7A |
| High | P36405 | ADP-ribosylation factor-like protein 3 [OS=Homo sapiens] | 6 | ARL3 |
| High | P45877 | Peptidyl-prolyl cis-trans isomerase C [OS=Homo sapiens] | 10 | PPIC |
| High | Q8WXF1 | Paraspeckle component 1 [OS=Homo sapiens] | 4 | PSPC1 |
| High | P31153 | S-adenosylmethionine synthase isoform type-2 [OS=Homo sapiens] | 4 | MAT2A |
| High | P23497 | Nuclear autoantigen Sp-100 [OS=Homo sapiens] | 2 | SP100 |
| High | Q9P2I0 | Cleavage and polyadenylation specificity factor subunit 2 [OS=Homo sapiens] | 2 | CPSF2 |
| High | O43765 | Small glutamine-rich tetratricopeptide repeat-containing protein alpha [OS=Homo sapiens] | 7 | SGTA |
| High | Q99661 | Kinesin-like protein KIF2C [OS=Homo sapiens] | 3 | KIF2C |
| High | P40616 | ADP-ribosylation factor-like protein 1 [OS=Homo sapiens] | 9 | ARL1 |
| High | P98160 | Basement membrane-specific heparan sulfate proteoglycan core protein [OS=Homo sapiens] | 1 | HSPG2 |
| High | Q9BY49 | Peroxisomal trans-2-enoyl-CoA reductase [OS=Homo sapiens] | 11 | PECR |
| High | Q9Y2B0 | Protein canopy homolog 2 [OS=Homo sapiens] | 18 | CNPY2 |
| High | Q86VS8 | Protein Hook homolog 3 [OS=Homo sapiens] | 3 | HOOK3 |
| High | Q9UJY4 | ADP-ribosylation factor-binding protein GGA2 [OS=Homo sapiens] | 5 | GGA2 |
| High | Q9H6R4 | Nucleolar protein 6 [OS=Homo sapiens] | 5 | NOL6 |
| High | P62310 | U6 snRNA-associated Sm-like protein LSm3 [OS=Homo sapiens] | 12 | LSM3 |
| High | Q08257 | Quinone oxidoreductase [OS=Homo sapiens] | 8 | CRYZ |
| High | Q9BZF1 | Oxysterol-binding protein-related protein 8 [OS=Homo sapiens] | 1 | OSBPL8 |
| High | O00762 | Ubiquitin-conjugating enzyme E2 C [OS=Homo sapiens] | 16 | UBE2C |
| High | Q9BTY7 | Protein HGH1 homolog [OS=Homo sapiens] | 6 | HGH1 |
| High | P14678 | Small nuclear ribonucleoprotein-associated proteins B and B' [OS=Homo sapiens] | 3 | SNRPB |
| High | Q96T23 | Remodeling and spacing factor 1 [OS=Homo sapiens] | 1 | RSF1 |
| High | Q99961 | Endophilin-A2 [OS=Homo sapiens] | 4 | SH3GL1 |
| High | Q9H930 | Nuclear body protein SP140-like protein [OS=Homo sapiens] | 3 | SP140L |
| High | O75146 | Huntingtin-interacting protein 1-related protein [OS=Homo sapiens] | 3 | HIP1R |
| High | P21359 | Neurofibromin [OS=Homo sapiens] | 1 | NF1 |
| High | P41226 | Ubiquitin-like modifier-activating enzyme 7 [OS=Homo sapiens] | 3 | UBA7 |
| High | Q969V6 | Myocardin-related transcription factor A [OS=Homo sapiens] | 3 | MRTFA |
| High | P35080 | Profilin-2 [OS=Homo sapiens] | 10 | PFN2 |
| High | P49023 | Paxillin [OS=Homo sapiens] | 3 | PXN |
| High | O95071 | E3 ubiquitin-protein ligase UBR5 [OS=Homo sapiens] | 1 | UBR5 |
| High | O75718 | Cartilage-associated protein [OS=Homo sapiens] | 4 | CRTAP |
| High | P62891 | 60S ribosomal protein L39 [OS=Homo sapiens] | 20 | RPL39 |
| High | P51946 | Cyclin-H [OS=Homo sapiens] | 8 | CCNH |
| High | Q3ZCQ8 | Mitochondrial import inner membrane translocase subunit TIM50 [OS=Homo sapiens] | 3 | TIMM50 |
| High | Q7L9L4 | MOB kinase activator 1B [OS=Homo sapiens] | 5 | MOB1B |
| High | Q9Y520 | Protein PRRC2C [OS=Homo sapiens] | 1 | PRRC2C |
| High | Q9HAV4 | Exportin-5 [OS=Homo sapiens] | 1 | XPO5 |
| High | P07339 | Cathepsin D [OS=Homo sapiens] | 2 | CTSD |
| High | Q99627 | COP9 signalosome complex subunit 8 [OS=Homo sapiens] | 10 | COPS8 |
| High | O75489 | NADH dehydrogenase [ubiquinone] iron-sulfur protein 3, mitochondrial [OS=Homo sapiens] | 11 | NDUFS3 |
| High | Q01081 | Splicing factor U2AF 35 kDa subunit [OS=Homo sapiens] | 8 | U2AF1 |
| High | Q12962 | Transcription initiation factor TFIID subunit 10 [OS=Homo sapiens] | 11 | TAF10 |
| High | P49903 | Selenide, water dikinase 1 [OS=Homo sapiens] | 4 | SEPHS1 |
| High | P79522 | Proline-rich protein 3 [OS=Homo sapiens] | 9 | PRR3 |
| High | Q9Y3I0 | RNA-splicing ligase RtcB homolog [OS=Homo sapiens] | 3 | RTCB |
| High | Q13045 | Protein flightless-1 homolog [OS=Homo sapiens] | 1 | FLII |
| High | Q9NY33 | Dipeptidyl peptidase 3 [OS=Homo sapiens] | 4 | DPP3 |
| High | Q9UIJ7 | GTP:AMP phosphotransferase AK3, mitochondrial [OS=Homo sapiens] | 6 | AK3 |
| High | O43324 | Eukaryotic translation elongation factor 1 epsilon-1 [OS=Homo sapiens] | 14 | EEF1E1 |
| High | Q9Y2X3 | Nucleolar protein 58 [OS=Homo sapiens] | 3 | NOP58 |
| High | Q14739 | Delta(14)-sterol reductase LBR [OS=Homo sapiens] | 2 | LBR |
| High | O15066 | Kinesin-like protein KIF3B [OS=Homo sapiens] | 3 | KIF3B |
| High | Q99426 | Tubulin-folding cofactor B [OS=Homo sapiens] | 10 | TBCB |
| High | O76071 | Probable cytosolic iron-sulfur protein assembly protein CIAO1 [OS=Homo sapiens] | 7 | CIAO1 |
| High | Q99575 | Ribonucleases P/MRP protein subunit POP1 [OS=Homo sapiens] | 3 | POP1 |
| High | Q9H0D6 | 5'-3' exoribonuclease 2 [OS=Homo sapiens] | 2 | XRN2 |
| High | P02461 | Collagen alpha-1(III) chain [OS=Homo sapiens] | 2 | COL3A1 |
| High | Q15102 | Platelet-activating factor acetylhydrolase IB subunit alpha1 [OS=Homo sapiens] | 4 | PAFAH1B3 |
| High | Q9NWU2 | Glucose-induced degradation protein 8 homolog [OS=Homo sapiens] | 11 | GID8 |
| High | Q9Y4E8 | Ubiquitin carboxyl-terminal hydrolase 15 [OS=Homo sapiens] | 2 | USP15 |
| High | P49643 | DNA primase large subunit [OS=Homo sapiens] | 2 | PRIM2 |
| High | Q9HB90 | Ras-related GTP-binding protein C [OS=Homo sapiens] | 6 | RRAGC |
| High | P28340 | DNA polymerase delta catalytic subunit [OS=Homo sapiens] | 3 | POLD1 |
| High | Q9UNS2 | COP9 signalosome complex subunit 3 [OS=Homo sapiens] | 5 | COPS3 |
| High | P55010 | Eukaryotic translation initiation factor 5 [OS=Homo sapiens] | 2 | EIF5 |
| High | Q13425 | Beta-2-syntrophin [OS=Homo sapiens] | 2 | SNTB2 |
| High | P83916 | Chromobox protein homolog 1 [OS=Homo sapiens] | 16 | CBX1 |
| High | Q9Y333 | U6 snRNA-associated Sm-like protein LSm2 [OS=Homo sapiens] | 20 | LSM2 |
| High | Q92538 | Golgi-specific brefeldin A-resistance guanine nucleotide exchange factor 1 [OS=Homo sapiens] | 2 | GBF1 |
| High | Q969Z0 | FAST kinase domain-containing protein 4 [OS=Homo sapiens] | 3 | TBRG4 |
| High | Q9Y446 | Plakophilin-3 [OS=Homo sapiens] | 2 | PKP3 |
| High | Q96FJ0 | AMSH-like protease [OS=Homo sapiens] | 5 | STAMBPL1 |
| High | Q9NXH9 | tRNA (guanine(26)-N(2))-dimethyltransferase [OS=Homo sapiens] | 4 | TRMT1 |
| High | Q16850 | Lanosterol 14-alpha demethylase [OS=Homo sapiens] | 4 | CYP51A1 |
| High | Q13546 | Receptor-interacting serine/threonine-protein kinase 1 [OS=Homo sapiens] | 1 | RIPK1 |
| High | Q96AY3 | Peptidyl-prolyl cis-trans isomerase FKBP10 [OS=Homo sapiens] | 2 | FKBP10 |
| High | P51608 | Methyl-CpG-binding protein 2 [OS=Homo sapiens] | 3 | MECP2 |
| High | Q9HCD5 | Nuclear receptor coactivator 5 [OS=Homo sapiens] | 3 | NCOA5 |
| High | Q9NVP2 | Histone chaperone ASF1B [OS=Homo sapiens] | 14 | ASF1B |
| High | Q12769 | Nuclear pore complex protein Nup160 [OS=Homo sapiens] | 2 | NUP160 |
| High | Q16851 | UTP--glucose-1-phosphate uridylyltransferase [OS=Homo sapiens] | 4 | UGP2 |
| High | Q5JTZ9 | Alanine--tRNA ligase, mitochondrial [OS=Homo sapiens] | 3 | AARS2 |
| High | P80303 | Nucleobindin-2 [OS=Homo sapiens] | 2 | NUCB2 |
| High | Q9P1F3 | Costars family protein ABRACL [OS=Homo sapiens] | 16 | ABRACL |
| High | Q9UBG0 | C-type mannose receptor 2 [OS=Homo sapiens] | 1 | MRC2 |
| High | O75822 | Eukaryotic translation initiation factor 3 subunit J [OS=Homo sapiens] | 5 | EIF3J |
| High | Q92896 | Golgi apparatus protein 1 [OS=Homo sapiens] | 1 | GLG1 |
| High | Q96N67 | Dedicator of cytokinesis protein 7 [OS=Homo sapiens] | 1 | DOCK7 |
| High | Q8NI27 | THO complex subunit 2 [OS=Homo sapiens] | 1 | THOC2 |
| High | Q9H4M9 | EH domain-containing protein 1 [OS=Homo sapiens] | 5 | EHD1 |
| High | O14734 | Acyl-coenzyme A thioesterase 8 [OS=Homo sapiens] | 6 | ACOT8 |
| High | Q8NBU5 | Outer mitochondrial transmembrane helix translocase [OS=Homo sapiens] | 4 | ATAD1 |
| High | P40763 | Signal transducer and activator of transcription 3 [OS=Homo sapiens] | 2 | STAT3 |
| High | A5YKK6 | CCR4-NOT transcription complex subunit 1 [OS=Homo sapiens] | 0 | CNOT1 |
| High | P56385 | ATP synthase subunit e, mitochondrial [OS=Homo sapiens] | 19 | ATP5ME |
| High | Q14160 | Protein scribble homolog [OS=Homo sapiens] | 2 | SCRIB |
| High | Q8TBQ9 | Protein kish-A [OS=Homo sapiens] | 13 | TMEM167A |
| High | Q16864 | V-type proton ATPase subunit F [OS=Homo sapiens] | 20 | ATP6V1F |
| High | Q71RC2 | La-related protein 4 [OS=Homo sapiens] | 2 | LARP4 |
| High | Q7Z3C6 | Autophagy-related protein 9A [OS=Homo sapiens] | 5 | ATG9A |
| High | P62328 | Thymosin beta-4 [OS=Homo sapiens] | 30 | TMSB4X |
| High | P35249 | Replication factor C subunit 4 [OS=Homo sapiens] | 2 | RFC4 |
| High | Q15008 | 26S proteasome non-ATPase regulatory subunit 6 [OS=Homo sapiens] | 4 | PSMD6 |
| High | Q92552 | 28S ribosomal protein S27, mitochondrial [OS=Homo sapiens] | 3 | MRPS27 |
| High | O14828 | Secretory carrier-associated membrane protein 3 [OS=Homo sapiens] | 4 | SCAMP3 |
| High | Q96SU4 | Oxysterol-binding protein-related protein 9 [OS=Homo sapiens] | 1 | OSBPL9 |
| High | Q9H223 | EH domain-containing protein 4 [OS=Homo sapiens] | 3 | EHD4 |
| High | O94804 | Serine/threonine-protein kinase 10 [OS=Homo sapiens] | 2 | STK10 |
| High | O75521 | Enoyl-CoA delta isomerase 2 [OS=Homo sapiens] | 4 | ECI2 |
| High | P04004 | Vitronectin [OS=Homo sapiens] | 3 | VTN |
| High | Q9NR28 | Diablo IAP-binding mitochondrial protein [OS=Homo sapiens] | 4 | DIABLO |
| High | Q8WUM0 | Nuclear pore complex protein Nup133 [OS=Homo sapiens] | 1 | NUP133 |
| High | P28070 | Proteasome subunit beta type-4 [OS=Homo sapiens] | 9 | PSMB4 |
| High | P62312 | U6 snRNA-associated Sm-like protein LSm6 [OS=Homo sapiens] | 33 | LSM6 |
| High | Q9Y2Z0 | Protein SGT1 homolog [OS=Homo sapiens] | 6 | SUGT1 |
| High | P48444 | Coatomer subunit delta [OS=Homo sapiens] | 2 | ARCN1 |
| High | P62304 | Small nuclear ribonucleoprotein E [OS=Homo sapiens] | 12 | SNRPE |
| High | P04181 | Ornithine aminotransferase, mitochondrial [OS=Homo sapiens] | 6 | OAT |
| High | Q9UI12 | V-type proton ATPase subunit H [OS=Homo sapiens] | 5 | ATP6V1H |
| High | Q969H8 | Myeloid-derived growth factor [OS=Homo sapiens] | 6 | MYDGF |
| High | Q9P0J0 | NADH dehydrogenase [ubiquinone] 1 alpha subcomplex subunit 13 [OS=Homo sapiens] | 9 | NDUFA13 |
| High | Q5GLZ8 | Probable E3 ubiquitin-protein ligase HERC4 [OS=Homo sapiens] | 2 | HERC4 |
| High | Q9UBB4 | Ataxin-10 [OS=Homo sapiens] | 2 | ATXN10 |
| High | Q96B26 | Exosome complex component RRP43 [OS=Homo sapiens] | 10 | EXOSC8 |
| High | Q9BQB6 | Vitamin K epoxide reductase complex subunit 1 [OS=Homo sapiens] | 8 | VKORC1 |
| High | O00442 | RNA 3'-terminal phosphate cyclase [OS=Homo sapiens] | 4 | RTCA |
| High | Q9NQ88 | Fructose-2,6-bisphosphatase TIGAR [OS=Homo sapiens] | 4 | TIGAR |
| High | Q13867 | Bleomycin hydrolase [OS=Homo sapiens] | 2 | BLMH |
| High | Q9BXF6 | Rab11 family-interacting protein 5 [OS=Homo sapiens] | 4 | RAB11FIP5 |
| High | Q9Y6M7 | Sodium bicarbonate cotransporter 3 [OS=Homo sapiens] | 2 | SLC4A7 |
| High | P43307 | Translocon-associated protein subunit alpha [OS=Homo sapiens] | 5 | SSR1 |
| High | Q9BRL6 | Serine/arginine-rich splicing factor 8 [OS=Homo sapiens] | 3 | SRSF8 |
| High | P19404 | NADH dehydrogenase [ubiquinone] flavoprotein 2, mitochondrial [OS=Homo sapiens] | 5 | NDUFV2 |
| High | P57088 | Transmembrane protein 33 [OS=Homo sapiens] | 5 | TMEM33 |
| High | Q9UIL1 | Short coiled-coil protein [OS=Homo sapiens] | 11 | SCOC |
| High | P46108 | Adapter molecule crk [OS=Homo sapiens] | 6 | CRK |
| High | O00154 | Cytosolic acyl coenzyme A thioester hydrolase [OS=Homo sapiens] | 3 | ACOT7 |
| High | Q8WUF5 | RelA-associated inhibitor [OS=Homo sapiens] | 3 | PPP1R13L |
| High | Q9H3N1 | Thioredoxin-related transmembrane protein 1 [OS=Homo sapiens] | 4 | TMX1 |
| High | Q9BZH6 | WD repeat-containing protein 11 [OS=Homo sapiens] | 1 | WDR11 |
| High | P31937 | 3-hydroxyisobutyrate dehydrogenase, mitochondrial [OS=Homo sapiens] | 4 | HIBADH |
| High | P06703 | Protein S100-A6 [OS=Homo sapiens] | 9 | S100A6 |
| High | Q9BV86 | N-terminal Xaa-Pro-Lys N-methyltransferase 1 [OS=Homo sapiens] | 3 | NTMT1 |
| High | Q96CS2 | HAUS augmin-like complex subunit 1 [OS=Homo sapiens] | 7 | HAUS1 |
| High | P37198 | Nuclear pore glycoprotein p62 [OS=Homo sapiens] | 3 | NUP62 |
| High | Q15654 | Thyroid receptor-interacting protein 6 [OS=Homo sapiens] | 5 | TRIP6 |
| High | O15173 | Membrane-associated progesterone receptor component 2 [OS=Homo sapiens] | 6 | PGRMC2 |
| High | Q9H444 | Charged multivesicular body protein 4b [OS=Homo sapiens] | 7 | CHMP4B |
| High | P19623 | Spermidine synthase [OS=Homo sapiens] | 3 | SRM |
| High | P36542 | ATP synthase subunit gamma, mitochondrial [OS=Homo sapiens] | 4 | ATP5F1C |
| High | Q9H773 | dCTP pyrophosphatase 1 [OS=Homo sapiens] | 12 | DCTPP1 |
| High | Q7Z4H8 | Protein O-glucosyltransferase 3 [OS=Homo sapiens] | 3 | POGLUT3 |
| High | Q7Z3E5 | LisH domain-containing protein ARMC9 [OS=Homo sapiens] | 3 | ARMC9 |
| High | Q14457 | Beclin-1 [OS=Homo sapiens] | 2 | BECN1 |
| High | Q01658 | Protein Dr1 [OS=Homo sapiens] | 14 | DR1 |
| High | P37268 | Squalene synthase [OS=Homo sapiens] | 4 | FDFT1 |
| High | Q7L7V1 | Putative pre-mRNA-splicing factor ATP-dependent RNA helicase DHX32 [OS=Homo sapiens] | 3 | DHX32 |
| High | P33240 | Cleavage stimulation factor subunit 2 [OS=Homo sapiens] | 5 | CSTF2 |
| High | Q13907 | Isopentenyl-diphosphate Delta-isomerase 1 [OS=Homo sapiens] | 9 | IDI1 |
| High | P26232 | Catenin alpha-2 [OS=Homo sapiens] | 2 | CTNNA2 |
| High | P54687 | Branched-chain-amino-acid aminotransferase, cytosolic [OS=Homo sapiens] | 5 | BCAT1 |
| High | O75477 | Erlin-1 [OS=Homo sapiens] | 3 | ERLIN1 |
| High | Q9NR12 | PDZ and LIM domain protein 7 [OS=Homo sapiens] | 3 | PDLIM7 |
| High | O43813 | Glutathione S-transferase LANCL1 [OS=Homo sapiens] | 6 | LANCL1 |
| High | Q8TD55 | Pleckstrin homology domain-containing family O member 2 [OS=Homo sapiens] | 5 | PLEKHO2 |
| High | P50336 | Protoporphyrinogen oxidase [OS=Homo sapiens] | 3 | PPOX |
| High | Q9BZJ0 | Crooked neck-like protein 1 [OS=Homo sapiens] | 2 | CRNKL1 |
| High | Q9H0A8 | COMM domain-containing protein 4 [OS=Homo sapiens] | 10 | COMMD4 |
| High | Q9BXS5 | AP-1 complex subunit mu-1 [OS=Homo sapiens] | 3 | AP1M1 |
| High | P63279 | SUMO-conjugating enzyme UBC9 [OS=Homo sapiens] | 6 | UBE2I |
| High | Q9NVZ3 | Adaptin ear-binding coat-associated protein 2 [OS=Homo sapiens] | 19 | NECAP2 |
| High | Q12874 | Splicing factor 3A subunit 3 [OS=Homo sapiens] | 2 | SF3A3 |
| High | Q6IPU0 | Centromere protein P [OS=Homo sapiens] | 6 | CENPP |
| High | P20810 | Calpastatin [OS=Homo sapiens] | 3 | CAST |
| High | Q96HY7 | 2-oxoadipate dehydrogenase complex component E1 [OS=Homo sapiens] | 1 | DHTKD1 |
| High | P21266 | Glutathione S-transferase Mu 3 [OS=Homo sapiens] | 8 | GSTM3 |
| High | P36959 | GMP reductase 1 [OS=Homo sapiens] | 6 | GMPR |
| High | P55769 | NHP2-like protein 1 [OS=Homo sapiens] | 9 | SNU13 |
| High | Q96ND0 | Protein FAM210A [OS=Homo sapiens] | 8 | FAM210A |
| High | Q9NRW7 | Vacuolar protein sorting-associated protein 45 [OS=Homo sapiens] | 2 | VPS45 |
| High | P41567 | Eukaryotic translation initiation factor 1 [OS=Homo sapiens] | 16 | EIF1 |
| High | Q9NUQ9 | CYFIP-related Rac1 interactor B [OS=Homo sapiens] | 4 | CYRIB |
| High | Q14232 | Translation initiation factor eIF-2B subunit alpha [OS=Homo sapiens] | 8 | EIF2B1 |
| High | Q9UIA9 | Exportin-7 [OS=Homo sapiens] | 1 | XPO7 |
| High | Q08379 | Golgin subfamily A member 2 [OS=Homo sapiens] | 1 | GOLGA2 |
| High | P36954 | DNA-directed RNA polymerase II subunit RPB9 [OS=Homo sapiens] | 18 | POLR2I |
| High | Q9UHY1 | Nuclear receptor-binding protein [OS=Homo sapiens] | 3 | NRBP1 |
| High | P09525 | Annexin A4 [OS=Homo sapiens] | 5 | ANXA4 |
| High | Q6IBS0 | Twinfilin-2 [OS=Homo sapiens] | 5 | TWF2 |
| High | Q86X76 | Deaminated glutathione amidase [OS=Homo sapiens] | 10 | NIT1 |
| High | Q9BXP5 | Serrate RNA effector molecule homolog [OS=Homo sapiens] | 1 | SRRT |
| High | Q99943 | 1-acyl-sn-glycerol-3-phosphate acyltransferase alpha [OS=Homo sapiens] | 7 | AGPAT1 |
| High | P46087 | Probable 28S rRNA (cytosine(4447)-C(5))-methyltransferase [OS=Homo sapiens] | 1 | NOP2 |
| High | Q9BWF3 | RNA-binding protein 4 [OS=Homo sapiens] | 9 | RBM4 |
| High | Q9Y4E1 | WASH complex subunit 2C [OS=Homo sapiens] | 2 | WASHC2C |
| High | Q92545 | Transmembrane protein 131 [OS=Homo sapiens] | 1 | TMEM131 |
| High | P54709 | Sodium/potassium-transporting ATPase subunit beta-3 [OS=Homo sapiens] | 5 | ATP1B3 |
| High | Q9GZR7 | ATP-dependent RNA helicase DDX24 [OS=Homo sapiens] | 3 | DDX24 |
| High | P42785 | Lysosomal Pro-X carboxypeptidase [OS=Homo sapiens] | 4 | PRCP |
| High | O43681 | ATPase GET3 [OS=Homo sapiens] | 3 | GET3 |
| High | Q9UBI6 | Guanine nucleotide-binding protein G(I)/G(S)/G(O) subunit gamma-12 [OS=Homo sapiens] | 22 | GNG12 |
| High | O00712 | Nuclear factor 1 B-type [OS=Homo sapiens] | 6 | NFIB |
| High | Q86U86 | Protein polybromo-1 [OS=Homo sapiens] | 1 | PBRM1 |
| High | Q86YV9 | BLOC-2 complex member HPS6 [OS=Homo sapiens] | 2 | HPS6 |
| High | P48147 | Prolyl endopeptidase [OS=Homo sapiens] | 2 | PREP |
| High | P26196 | Probable ATP-dependent RNA helicase DDX6 [OS=Homo sapiens] | 2 | DDX6 |
| High | Q15027 | Arf-GAP with coiled-coil, ANK repeat and PH domain-containing protein 1 [OS=Homo sapiens] | 2 | ACAP1 |
| High | P10301 | Ras-related protein R-Ras [OS=Homo sapiens] | 6 | RRAS |
| High | Q9BV38 | WD repeat-containing protein 18 [OS=Homo sapiens] | 3 | WDR18 |
| High | Q8N3U4 | Cohesin subunit SA-2 [OS=Homo sapiens] | 1 | STAG2 |
| High | Q8N2X6 | Uncharacterized protein EXOC3-AS1 [OS=Homo sapiens] | 18 | EXOC3-AS1 |
| High | Q9UMY4 | Sorting nexin-12 [OS=Homo sapiens] | 6 | SNX12 |
| High | P20020 | Plasma membrane calcium-transporting ATPase 1 [OS=Homo sapiens] | 2 | ATP2B1 |
| High | Q08752 | Peptidyl-prolyl cis-trans isomerase D [OS=Homo sapiens] | 3 | PPID |
| High | P56539 | Caveolin-3 [OS=Homo sapiens] | 7 | CAV3 |
| High | P49458 | Signal recognition particle 9 kDa protein [OS=Homo sapiens] | 13 | SRP9 |
| High | Q5T1J5 | Putative coiled-coil-helix-coiled-coil-helix domain-containing protein CHCHD2P9, mitochondrial [OS=Homo sapiens] | 19 | CHCHD2P9 |
| High | Q8WYA6 | Beta-catenin-like protein 1 [OS=Homo sapiens] | 1 | CTNNBL1 |
| High | P15291 | Beta-1,4-galactosyltransferase 1 [OS=Homo sapiens] | 2 | B4GALT1 |
| High | O60762 | Dolichol-phosphate mannosyltransferase subunit 1 [OS=Homo sapiens] | 8 | DPM1 |
| High | Q32P41 | tRNA (guanine(37)-N1)-methyltransferase [OS=Homo sapiens] | 5 | TRMT5 |
| High | O43264 | Centromere/kinetochore protein zw10 homolog [OS=Homo sapiens] | 3 | ZW10 |
| High | P49247 | Ribose-5-phosphate isomerase [OS=Homo sapiens] | 3 | RPIA |
| High | Q12841 | Follistatin-related protein 1 [OS=Homo sapiens] | 3 | FSTL1 |
| High | Q2TAY7 | WD40 repeat-containing protein SMU1 [OS=Homo sapiens] | 2 | SMU1 |
| High | O75351 | Vacuolar protein sorting-associated protein 4B [OS=Homo sapiens] | 3 | VPS4B |
| High | P61601 | Neurocalcin-delta [OS=Homo sapiens] | 4 | NCALD |
| High | Q6NXS1 | Protein phosphatase inhibitor 2 family member B [OS=Homo sapiens] | 7 | PPP1R2B |
| High | Q9UPT8 | Zinc finger CCCH domain-containing protein 4 [OS=Homo sapiens] | 1 | ZC3H4 |
| High | O96013 | Serine/threonine-protein kinase PAK 4 [OS=Homo sapiens] | 3 | PAK4 |
| High | P51668 | Ubiquitin-conjugating enzyme E2 D1 [OS=Homo sapiens] | 18 | UBE2D1 |
| High | Q9BZE9 | Tether containing UBX domain for GLUT4 [OS=Homo sapiens] | 3 | ASPSCR1 |
| High | Q13535 | Serine/threonine-protein kinase ATR [OS=Homo sapiens] | 0 | ATR |
| High | P02786 | Transferrin receptor protein 1 [OS=Homo sapiens] | 1 | TFRC |
| High | Q9BQ69 | ADP-ribose glycohydrolase MACROD1 [OS=Homo sapiens] | 4 | MACROD1 |
| High | Q9UHG3 | Prenylcysteine oxidase 1 [OS=Homo sapiens] | 5 | PCYOX1 |
| High | P37173 | TGF-beta receptor type-2 [OS=Homo sapiens] | 3 | TGFBR2 |
| High | O75352 | Mannose-P-dolichol utilization defect 1 protein [OS=Homo sapiens] | 4 | MPDU1 |
| High | O43294 | Transforming growth factor beta-1-induced transcript 1 protein [OS=Homo sapiens] | 2 | TGFB1I1 |
| High | P11177 | Pyruvate dehydrogenase E1 component subunit beta, mitochondrial [OS=Homo sapiens] | 2 | PDHB |
| High | Q08AM6 | Protein VAC14 homolog [OS=Homo sapiens] | 2 | VAC14 |
| High | O14521 | Succinate dehydrogenase [ubiquinone] cytochrome b small subunit, mitochondrial [OS=Homo sapiens] | 7 | SDHD |
| High | Q9HDC9 | Adipocyte plasma membrane-associated protein [OS=Homo sapiens] | 6 | APMAP |
| High | Q16795 | NADH dehydrogenase [ubiquinone] 1 alpha subcomplex subunit 9, mitochondrial [OS=Homo sapiens] | 3 | NDUFA9 |
| High | Q99747 | Gamma-soluble NSF attachment protein [OS=Homo sapiens] | 4 | NAPG |
| High | Q9BZX2 | Uridine-cytidine kinase 2 [OS=Homo sapiens] | 10 | UCK2 |
| High | Q9NT62 | Ubiquitin-like-conjugating enzyme ATG3 [OS=Homo sapiens] | 4 | ATG3 |
| High | Q9BV73 | Centrosome-associated protein CEP250 [OS=Homo sapiens] | 1 | CEP250 |
| High | Q9UMX0 | Ubiquilin-1 [OS=Homo sapiens] | 3 | UBQLN1 |
| High | Q9UNL2 | Translocon-associated protein subunit gamma [OS=Homo sapiens] | 8 | SSR3 |
| High | Q9UKN8 | General transcription factor 3C polypeptide 4 [OS=Homo sapiens] | 2 | GTF3C4 |
| High | Q15126 | Phosphomevalonate kinase [OS=Homo sapiens] | 7 | PMVK |
| High | Q8WWY3 | U4/U6 small nuclear ribonucleoprotein Prp31 [OS=Homo sapiens] | 3 | PRPF31 |
| High | Q14680 | Maternal embryonic leucine zipper kinase [OS=Homo sapiens] | 3 | MELK |
| High | Q66K74 | Microtubule-associated protein 1S [OS=Homo sapiens] | 4 | MAP1S |
| High | Q9NRY5 | Protein FAM114A2 [OS=Homo sapiens] | 8 | FAM114A2 |
| High | P22307 | Sterol carrier protein 2 [OS=Homo sapiens] | 1 | SCP2 |
| High | P11172 | Uridine 5'-monophosphate synthase [OS=Homo sapiens] | 4 | UMPS |
| High | P24539 | ATP synthase F(0) complex subunit B1, mitochondrial [OS=Homo sapiens] | 6 | ATP5PB |
| High | Q96NE9 | FERM domain-containing protein 6 [OS=Homo sapiens] | 3 | FRMD6 |
| High | Q9Y2F9 | BTB/POZ domain-containing protein 3 [OS=Homo sapiens] | 5 | BTBD3 |
| High | Q16539 | Mitogen-activated protein kinase 14 [OS=Homo sapiens] | 6 | MAPK14 |
| High | Q96JJ7 | Protein disulfide-isomerase TMX3 [OS=Homo sapiens] | 6 | TMX3 |
| High | O43633 | Charged multivesicular body protein 2a [OS=Homo sapiens] | 3 | CHMP2A |
| High | P51572 | B-cell receptor-associated protein 31 [OS=Homo sapiens] | 4 | BCAP31 |
| High | Q14166 | Tubulin--tyrosine ligase-like protein 12 [OS=Homo sapiens] | 4 | TTLL12 |
| High | P13995 | Bifunctional methylenetetrahydrofolate dehydrogenase/cyclohydrolase, mitochondrial [OS=Homo sapiens] | 7 | MTHFD2 |
| High | Q8IWX8 | Calcium homeostasis endoplasmic reticulum protein [OS=Homo sapiens] | 3 | CHERP |
| High | Q68CP9 | AT-rich interactive domain-containing protein 2 [OS=Homo sapiens] | 0 | ARID2 |
| High | P54105 | Methylosome subunit pICln [OS=Homo sapiens] | 11 | CLNS1A |
| High | Q9BUT1 | Dehydrogenase/reductase SDR family member 6 [OS=Homo sapiens] | 9 | BDH2 |
| High | P60059 | Protein transport protein Sec61 subunit gamma [OS=Homo sapiens] | 18 | SEC61G |
| High | P40394 | All-trans-retinol dehydrogenase [NAD(+)] ADH7 [OS=Homo sapiens] | 7 | ADH7 |
| High | O43290 | U4/U6.U5 tri-snRNP-associated protein 1 [OS=Homo sapiens] | 2 | SART1 |
| High | Q1ED39 | Lysine-rich nucleolar protein 1 [OS=Homo sapiens] | 2 | KNOP1 |
| High | P10515 | Dihydrolipoyllysine-residue acetyltransferase component of pyruvate dehydrogenase complex, mitochondrial [OS=Homo sapiens] | 3 | DLAT |
| High | P48507 | Glutamate--cysteine ligase regulatory subunit [OS=Homo sapiens] | 6 | GCLM |
| High | Q7Z7L1 | Schlafen family member 11 [OS=Homo sapiens] | 2 | SLFN11 |
| High | Q53GQ0 | Very-long-chain 3-oxoacyl-CoA reductase [OS=Homo sapiens] | 5 | HSD17B12 |
| High | Q9NRH2 | SNF-related serine/threonine-protein kinase [OS=Homo sapiens] | 4 | SNRK |
| High | P67870 | Casein kinase II subunit beta [OS=Homo sapiens] | 4 | CSNK2B |
| High | Q6ZXV5 | Protein O-mannosyl-transferase TMTC3 [OS=Homo sapiens] | 1 | TMTC3 |
| High | Q9C0C2 | 182 kDa tankyrase-1-binding protein [OS=Homo sapiens] | 3 | TNKS1BP1 |
| High | P80217 | Interferon-induced 35 kDa protein [OS=Homo sapiens] | 7 | IFI35 |
| High | Q96CW1 | AP-2 complex subunit mu [OS=Homo sapiens] | 2 | AP2M1 |
| High | Q9NZL4 | Hsp70-binding protein 1 [OS=Homo sapiens] | 3 | HSPBP1 |
| High | Q14254 | Flotillin-2 [OS=Homo sapiens] | 3 | FLOT2 |
| High | Q14997 | Proteasome activator complex subunit 4 [OS=Homo sapiens] | 1 | PSME4 |
| High | Q9Y2D5 | A-kinase anchor protein 2 [OS=Homo sapiens] | 2 | AKAP2 |
| High | Q9NQT8 | Kinesin-like protein KIF13B [OS=Homo sapiens] | 1 | KIF13B |
| High | P61960 | Ubiquitin-fold modifier 1 [OS=Homo sapiens] | 18 | UFM1 |
| High | Q99459 | Cell division cycle 5-like protein [OS=Homo sapiens] | 1 | CDC5L |
| High | Q14683 | Structural maintenance of chromosomes protein 1A [OS=Homo sapiens] | 1 | SMC1A |
| High | P21912 | Succinate dehydrogenase [ubiquinone] iron-sulfur subunit, mitochondrial [OS=Homo sapiens] | 3 | SDHB |
| High | Q9UKB1 | F-box/WD repeat-containing protein 11 [OS=Homo sapiens] | 5 | FBXW11 |
| High | Q9BX68 | Adenosine 5'-monophosphoramidase HINT2 [OS=Homo sapiens] | 12 | HINT2 |
| High | Q92572 | AP-3 complex subunit sigma-1 [OS=Homo sapiens] | 9 | AP3S1 |
| High | P11166 | Solute carrier family 2, facilitated glucose transporter member 1 [OS=Homo sapiens] | 2 | SLC2A1 |
| High | Q9NZM1 | Myoferlin [OS=Homo sapiens] | 1 | MYOF |
| High | Q8WTU0 | Protein DDI1 homolog 1 [OS=Homo sapiens] | 3 | DDI1 |
| High | Q8TDD1 | ATP-dependent RNA helicase DDX54 [OS=Homo sapiens] | 2 | DDX54 |
| High | Q96HC4 | PDZ and LIM domain protein 5 [OS=Homo sapiens] | 2 | PDLIM5 |
| High | Q8N9N2 | Activating signal cointegrator 1 complex subunit 1 [OS=Homo sapiens] | 4 | ASCC1 |
| High | Q9HD33 | 39S ribosomal protein L47, mitochondrial [OS=Homo sapiens] | 5 | MRPL47 |
| High | P09012 | U1 small nuclear ribonucleoprotein A [OS=Homo sapiens] | 3 | SNRPA |
| High | Q3LIE5 | Manganese-dependent ADP-ribose/CDP-alcohol diphosphatase [OS=Homo sapiens] | 7 | ADPRM |
| High | Q9UQ35 | Serine/arginine repetitive matrix protein 2 [OS=Homo sapiens] | 1 | SRRM2 |
| High | O14776 | Transcription elongation regulator 1 [OS=Homo sapiens] | 1 | TCERG1 |
| High | P23919 | Thymidylate kinase [OS=Homo sapiens] | 4 | DTYMK |
| High | O43396 | Thioredoxin-like protein 1 [OS=Homo sapiens] | 2 | TXNL1 |
| High | P53680 | AP-2 complex subunit sigma [OS=Homo sapiens] | 5 | AP2S1 |
| High | A6ND91 | Aspartate dehydrogenase domain-containing protein [OS=Homo sapiens] | 3 | ASPDH |
| High | P13861 | cAMP-dependent protein kinase type II-alpha regulatory subunit [OS=Homo sapiens] | 4 | PRKAR2A |
| High | O43617 | Trafficking protein particle complex subunit 3 [OS=Homo sapiens] | 4 | TRAPPC3 |
| High | Q9NZV5 | Selenoprotein N [OS=Homo sapiens] | 3 | SELENON |
| High | P53004 | Biliverdin reductase A [OS=Homo sapiens] | 6 | BLVRA |
| High | Q96JB5 | CDK5 regulatory subunit-associated protein 3 [OS=Homo sapiens] | 3 | CDK5RAP3 |
| High | Q9BPX5 | Actin-related protein 2/3 complex subunit 5-like protein [OS=Homo sapiens] | 8 | ARPC5L |
| High | Q9BZQ8 | Protein Niban 1 [OS=Homo sapiens] | 2 | NIBAN1 |
| High | Q9Y5Y2 | Cytosolic Fe-S cluster assembly factor NUBP2 [OS=Homo sapiens] | 10 | NUBP2 |
| High | O00625 | Pirin [OS=Homo sapiens] | 4 | PIR |
| High | O60493 | Sorting nexin-3 [OS=Homo sapiens] | 6 | SNX3 |
| High | O75431 | Metaxin-2 [OS=Homo sapiens] | 8 | MTX2 |
| High | P10253 | Lysosomal alpha-glucosidase [OS=Homo sapiens] | 2 | GAA |
| High | Q5JPE7 | BOS complex subunit NOMO2 [OS=Homo sapiens] | 2 | NOMO2 |
| High | P84090 | Enhancer of rudimentary homolog [OS=Homo sapiens] | 6 | ERH |
| High | O75368 | Adapter SH3BGRL [OS=Homo sapiens] | 16 | SH3BGRL |
| High | Q5JRA6 | Transport and Golgi organization protein 1 homolog [OS=Homo sapiens] | 1 | MIA3 |
| High | Q13573 | SNW domain-containing protein 1 [OS=Homo sapiens] | 3 | SNW1 |
| High | Q9BXJ9 | N-alpha-acetyltransferase 15, NatA auxiliary subunit [OS=Homo sapiens] | 2 | NAA15 |
| High | Q9Y5P6 | Mannose-1-phosphate guanyltransferase beta [OS=Homo sapiens] | 6 | GMPPB |
| High | P61326 | Protein mago nashi homolog [OS=Homo sapiens] | 14 | MAGOH |
| High | Q9H3K6 | BolA-like protein 2 [OS=Homo sapiens] | 19 | BOLA2; BOLA2B |
| High | Q9UKV3 | Apoptotic chromatin condensation inducer in the nucleus [OS=Homo sapiens] | 1 | ACIN1 |
| High | P37108 | Signal recognition particle 14 kDa protein [OS=Homo sapiens] | 13 | SRP14 |
| High | Q99490 | Arf-GAP with GTPase, ANK repeat and PH domain-containing protein 2 [OS=Homo sapiens] | 1 | AGAP2 |
| High | Q8WXQ8 | Carboxypeptidase A5 [OS=Homo sapiens] | 2 | CPA5 |
| High | Q14432 | cGMP-inhibited 3',5'-cyclic phosphodiesterase 3A [OS=Homo sapiens] | 2 | PDE3A |
| High | P55283 | Cadherin-4 [OS=Homo sapiens] | 2 | CDH4 |
| High | O75381 | Peroxisomal membrane protein PEX14 [OS=Homo sapiens] | 10 | PEX14 |
| High | Q96RS6 | NudC domain-containing protein 1 [OS=Homo sapiens] | 2 | NUDCD1 |
| Medium | Q7Z7G8 | Intermembrane lipid transfer protein VPS13B [OS=Homo sapiens] | 0 | VPS13B |
| Medium | P62308 | Small nuclear ribonucleoprotein G [OS=Homo sapiens] | 9 | SNRPG |
| Medium | P08F94 | Fibrocystin [OS=Homo sapiens] | 0 | PKHD1 |
| Medium | Q6PCB7 | Long-chain fatty acid transport protein 1 [OS=Homo sapiens] | 4 | SLC27A1 |
| Medium | Q9P2F8 | Signal-induced proliferation-associated 1-like protein 2 [OS=Homo sapiens] | 1 | SIPA1L2 |
| Medium | Q5XPI4 | E3 ubiquitin-protein ligase RNF123 [OS=Homo sapiens] | 1 | RNF123 |
| Medium | Q15024 | Exosome complex component RRP42 [OS=Homo sapiens] | 8 | EXOSC7 |
| Medium | Q96C45 | Serine/threonine-protein kinase ULK4 [OS=Homo sapiens] | 1 | ULK4 |
| Medium | P60900 | Proteasome subunit alpha type-6 [OS=Homo sapiens] | 7 | PSMA6 |
| Medium | P49790 | Nuclear pore complex protein Nup153 [OS=Homo sapiens] | 1 | NUP153 |
| Medium | Q8TD16 | Protein bicaudal D homolog 2 [OS=Homo sapiens] | 3 | BICD2 |
| Medium | Q9NZZ3 | Charged multivesicular body protein 5 [OS=Homo sapiens] | 7 | CHMP5 |
| Medium | Q969S9 | Ribosome-releasing factor 2, mitochondrial [OS=Homo sapiens] | 4 | GFM2 |
| Medium | P32456 | Guanylate-binding protein 2 [OS=Homo sapiens] | 3 | GBP2 |
| Medium | Q14146 | Unhealthy ribosome biogenesis protein 2 homolog [OS=Homo sapiens] | 2 | URB2 |
| Medium | P09234 | U1 small nuclear ribonucleoprotein C [OS=Homo sapiens] | 11 | SNRPC |
| Medium | Q15032 | R3H domain-containing protein 1 [OS=Homo sapiens] | 1 | R3HDM1 |
| Medium | Q8IXB1 | DnaJ homolog subfamily C member 10 [OS=Homo sapiens] | 2 | DNAJC10 |
| Medium | Q8NF91 | Nesprin-1 [OS=Homo sapiens] | 0 | SYNE1 |
| Medium | O43865 | S-adenosylhomocysteine hydrolase-like protein 1 [OS=Homo sapiens] | 2 | AHCYL1 |
| Medium | P49642 | DNA primase small subunit [OS=Homo sapiens] | 4 | PRIM1 |
| Medium | P07108 | Acyl-CoA-binding protein [OS=Homo sapiens] | 11 | DBI |
| Medium | Q13625 | Apoptosis-stimulating of p53 protein 2 [OS=Homo sapiens] | 2 | TP53BP2 |
| Medium | P28331 | NADH-ubiquinone oxidoreductase 75 kDa subunit, mitochondrial [OS=Homo sapiens] | 2 | NDUFS1 |
| Medium | Q96JM3 | Chromosome alignment-maintaining phosphoprotein 1 [OS=Homo sapiens] | 1 | CHAMP1 |
| Medium | Q96CS3 | FAS-associated factor 2 [OS=Homo sapiens] | 4 | FAF2 |
| Medium | Q9BYT3 | Serine/threonine-protein kinase 33 [OS=Homo sapiens] | 2 | STK33 |
| Medium | Q6KC79 | Nipped-B-like protein [OS=Homo sapiens] | 1 | NIPBL |
| Medium | Q8IXQ3 | Uncharacterized protein C9orf40 [OS=Homo sapiens] | 14 | C9orf40 |
| Medium | Q8N1F8 | Serine/threonine-protein kinase 11-interacting protein [OS=Homo sapiens] | 2 | STK11IP |
| Medium | Q8N766 | ER membrane protein complex subunit 1 [OS=Homo sapiens] | 2 | EMC1 |
| Medium | P18754 | Regulator of chromosome condensation [OS=Homo sapiens] | 2 | RCC1 |
| Medium | O43156 | TELO2-interacting protein 1 homolog [OS=Homo sapiens] | 2 | TTI1 |
| Medium | Q9UMR2 | ATP-dependent RNA helicase DDX19B [OS=Homo sapiens] | 4 | DDX19B |
| Medium | Q92890 | Ubiquitin recognition factor in ER-associated degradation protein 1 [OS=Homo sapiens] | 5 | UFD1 |
| Medium | P11387 | DNA topoisomerase 1 [OS=Homo sapiens] | 2 | TOP1 |
| Medium | Q15392 | Delta(24)-sterol reductase [OS=Homo sapiens] | 2 | DHCR24 |
| Medium | Q8N137 | Centrobin [OS=Homo sapiens] | 1 | CNTROB |
| Medium | Q9Y6E0 | Serine/threonine-protein kinase 24 [OS=Homo sapiens] | 3 | STK24 |
| Medium | P54259 | Atrophin-1 [OS=Homo sapiens] | 1 | ATN1 |
| Medium | Q99848 | Probable rRNA-processing protein EBP2 [OS=Homo sapiens] | 4 | EBNA1BP2 |
| Medium | P0C7P4 | Putative cytochrome b-c1 complex subunit Rieske-like protein 1 [OS=Homo sapiens] | 9 | UQCRFS1P1 |
| Medium | P05161 | Ubiquitin-like protein ISG15 [OS=Homo sapiens] | 26 | ISG15 |
| Medium | O43464 | Serine protease HTRA2, mitochondrial [OS=Homo sapiens] | 6 | HTRA2 |
| Medium | Q63HN8 | E3 ubiquitin-protein ligase RNF213 [OS=Homo sapiens] | 0 | RNF213 |
| Medium | Q9Y6V0 | Protein piccolo [OS=Homo sapiens] | 0 | PCLO |
| Medium | P08648 | Integrin alpha-5 [OS=Homo sapiens] | 1 | ITGA5 |
| Medium | P07942 | Laminin subunit beta-1 [OS=Homo sapiens] | 1 | LAMB1 |
| Medium | O00217 | NADH dehydrogenase [ubiquinone] iron-sulfur protein 8, mitochondrial [OS=Homo sapiens] | 4 | NDUFS8 |
| Medium | A6NDG6 | Glycerol-3-phosphate phosphatase [OS=Homo sapiens] | 14 | PGP |
| Medium | O60828 | Polyglutamine-binding protein 1 [OS=Homo sapiens] | 12 | PQBP1 |
| Medium | Q8WXX0 | Dynein axonemal heavy chain 7 [OS=Homo sapiens] | 0 | DNAH7 |
| Medium | Q8TAT6 | Nuclear protein localization protein 4 homolog [OS=Homo sapiens] | 3 | NPLOC4 |
| Medium | P30043 | Flavin reductase (NADPH) [OS=Homo sapiens] | 5 | BLVRB |
| Medium | Q0VDF9 | Heat shock 70 kDa protein 14 [OS=Homo sapiens] | 6 | HSPA14 |
| Medium | Q5VWQ0 | Lysine-specific demethylase 9 [OS=Homo sapiens] | 2 | RSBN1 |
| Medium | O15116 | U6 snRNA-associated Sm-like protein LSm1 [OS=Homo sapiens] | 12 | LSM1 |
| Medium | P17931 | Galectin-3 [OS=Homo sapiens] | 3 | LGALS3 |
| Medium | Q6NUM9 | All-trans-retinol 13,14-reductase [OS=Homo sapiens] | 2 | RETSAT |
| Medium | Q9H9J2 | 39S ribosomal protein L44, mitochondrial [OS=Homo sapiens] | 5 | MRPL44 |
| Medium | Q13107 | Ubiquitin carboxyl-terminal hydrolase 4 [OS=Homo sapiens] | 2 | USP4 |
| Medium | Q8IWA0 | WD repeat-containing protein 75 [OS=Homo sapiens] | 2 | WDR75 |
| Medium | P04062 | Lysosomal acid glucosylceramidase [OS=Homo sapiens] | 4 | GBA1 |
| Medium | O95936 | Eomesodermin homolog [OS=Homo sapiens] | 4 | EOMES |
| Medium | Q9H207 | Olfactory receptor 10A5 [OS=Homo sapiens] | 7 | OR10A5 |
| Medium | P42330 | Aldo-keto reductase family 1 member C3 [OS=Homo sapiens] | 6 | AKR1C3 |
| Medium | Q14728 | Major facilitator superfamily domain-containing protein 10 [OS=Homo sapiens] | 3 | MFSD10 |
| Medium | Q9GZQ3 | COMM domain-containing protein 5 [OS=Homo sapiens] | 13 | COMMD5 |
| Medium | P46109 | Crk-like protein [OS=Homo sapiens] | 4 | CRKL |
| Medium | Q8NE01 | Metal transporter CNNM3 [OS=Homo sapiens] | 5 | CNNM3 |
| Medium | P27144 | Adenylate kinase 4, mitochondrial [OS=Homo sapiens] | 8 | AK4 |
| Medium | Q16658 | Fascin [OS=Homo sapiens] | 2 | FSCN1 |
| Medium | Q15181 | Inorganic pyrophosphatase [OS=Homo sapiens] | 5 | PPA1 |
| Medium | Q99417 | c-Myc-binding protein [OS=Homo sapiens] | 19 | MYCBP |
| Medium | P61513 | 60S ribosomal protein L37a [OS=Homo sapiens] | 9 | RPL37A |
| Medium | P60983 | Glia maturation factor beta [OS=Homo sapiens] | 18 | GMFB |
| Medium | O60437 | Periplakin [OS=Homo sapiens] | 0 | PPL |
| Medium | Q9ULT8 | E3 ubiquitin-protein ligase HECTD1 [OS=Homo sapiens] | 1 | HECTD1 |
| Medium | Q9GZT8 | NIF3-like protein 1 [OS=Homo sapiens] | 3 | NIF3L1 |
| Medium | Q08AE8 | Protein spire homolog 1 [OS=Homo sapiens] | 1 | SPIRE1 |
| Medium | O15294 | UDP-N-acetylglucosamine--peptide N-acetylglucosaminyltransferase 110 kDa subunit [OS=Homo sapiens] | 2 | OGT |
| Medium | Q6NUQ4 | Transmembrane protein 214 [OS=Homo sapiens] | 1 | TMEM214 |
| Medium | Q96A49 | Synapse-associated protein 1 [OS=Homo sapiens] | 4 | SYAP1 |
| Medium | Q9H7B2 | Ribosome production factor 2 homolog [OS=Homo sapiens] | 3 | RPF2 |
| Medium | O43252 | Bifunctional 3'-phosphoadenosine 5'-phosphosulfate synthase 1 [OS=Homo sapiens] | 3 | PAPSS1 |
| Medium | Q2WGJ8 | Cation channel sperm-associated auxiliary subunit TMEM249 [OS=Homo sapiens] | 9 | TMEM249 |
| Medium | P46939 | Utrophin [OS=Homo sapiens] | 1 | UTRN |
| Medium | Q9P035 | Very-long-chain (3R)-3-hydroxyacyl-CoA dehydratase 3 [OS=Homo sapiens] | 6 | HACD3 |
| Medium | Q9H9H5 | MAP6 domain-containing protein 1 [OS=Homo sapiens] | 12 | MAP6D1 |
| Medium | Q96KS9 | Protein FAM167A [OS=Homo sapiens] | 16 | FAM167A |
| Medium | Q9UBQ5 | Eukaryotic translation initiation factor 3 subunit K [OS=Homo sapiens] | 6 | EIF3K |
| Medium | P63165 | Small ubiquitin-related modifier 1 [OS=Homo sapiens] | 7 | SUMO1 |
| Medium | Q13395 | Probable methyltransferase TARBP1 [OS=Homo sapiens] | 1 | TARBP1 |
| Medium | O76003 | Glutaredoxin-3 [OS=Homo sapiens] | 3 | GLRX3 |
| Medium | Q9Y6E2 | eIF5-mimic protein 1 [OS=Homo sapiens] | 4 | BZW2 |
| Medium | Q9HC44 | Vasculin-like protein 1 [OS=Homo sapiens] | 2 | GPBP1L1 |
| Medium | Q02108 | Guanylate cyclase soluble subunit alpha-1 [OS=Homo sapiens] | 3 | GUCY1A1 |
| Medium | Q8N8Z3 | Putative uncharacterized protein DIP2C-AS1 [OS=Homo sapiens] | 14 | DIP2C-AS1 |
| Medium | Q15836 | Vesicle-associated membrane protein 3 [OS=Homo sapiens] | 17 | VAMP3 |
| Medium | Q8WZ42 | Titin [OS=Homo sapiens] | 0 | TTN |
| Medium | O60716 | Catenin delta-1 [OS=Homo sapiens] | 2 | CTNND1 |
| Medium | P08123 | Collagen alpha-2(I) chain [OS=Homo sapiens] | 1 | COL1A2 |
| Medium | Q9UPU5 | Ubiquitin carboxyl-terminal hydrolase 24 [OS=Homo sapiens] | 1 | USP24 |
| Medium | Q9UHJ6 | Sedoheptulokinase [OS=Homo sapiens] | 4 | SHPK |
| Medium | Q96N87 | Inactive sodium-dependent neutral amino acid transporter B(0)AT3 [OS=Homo sapiens] | 6 | SLC6A18 |
| Medium | O94776 | Metastasis-associated protein MTA2 [OS=Homo sapiens] | 3 | MTA2 |
| Medium | O00422 | Histone deacetylase complex subunit SAP18 [OS=Homo sapiens] | 10 | SAP18 |
| Medium | P25940 | Collagen alpha-3(V) chain [OS=Homo sapiens] | 2 | COL5A3 |
| Medium | P08621 | U1 small nuclear ribonucleoprotein 70 kDa [OS=Homo sapiens] | 3 | SNRNP70 |
| Medium | P06756 | Integrin alpha-V [OS=Homo sapiens] | 2 | ITGAV |
| Medium | Q13469 | Nuclear factor of activated T-cells, cytoplasmic 2 [OS=Homo sapiens] | 1 | NFATC2 |
| Medium | P21589 | 5'-nucleotidase [OS=Homo sapiens] | 4 | NT5E |

**Figure S1:** Graphical representation of the 20 most important molecules for classification obtained from the analysis of the VIP scores of PLS-DA for the lipidomics dataset.

**Figure S2.** Graphical representation of the 20 most important molecules for classification obtained from the analysis of the VIP scores of PLS-DA for the metabolomics dataset.

**
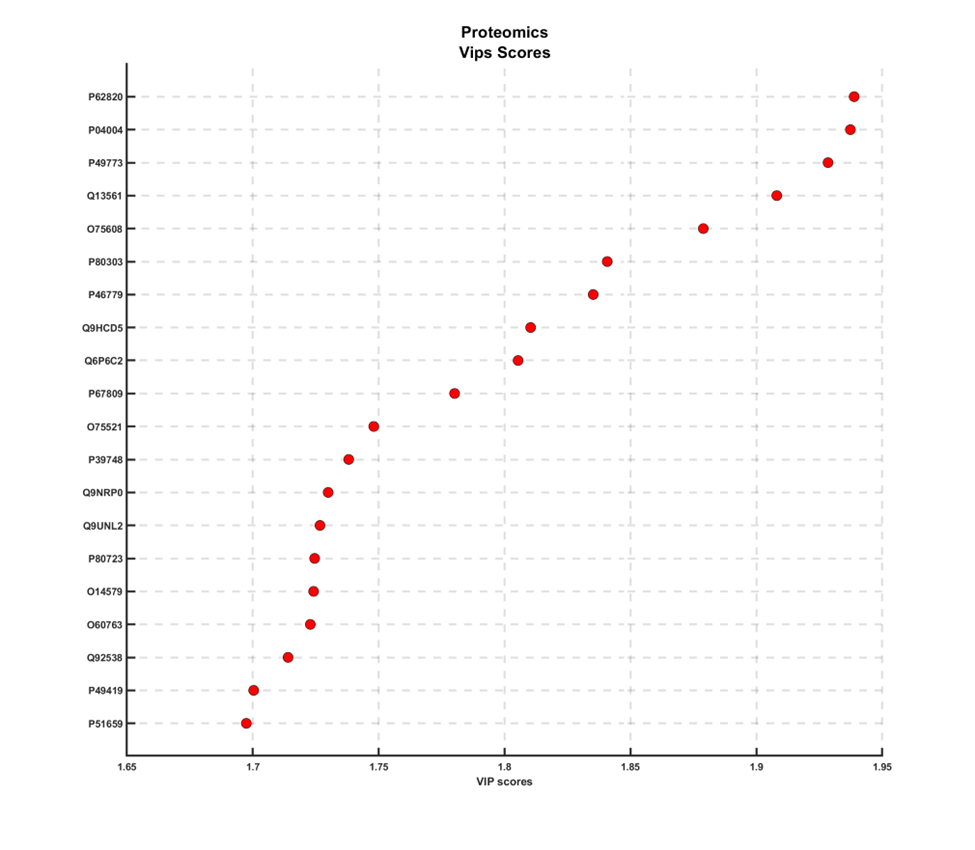
**

**Figure S3.** Graphical representation of the 20 most important molecules for classification obtained from the analysis of the VIP scores of PLS-DA for the Proteomics dataset.

**Table S4:** Values of PLS-DA performances. It reports the values of accuracy, sensitivity and specificity for the training set. Model built with all the modality independently. Four repetitions were used as a training set.

| Model | nLVs | CC (%) | Sensitivity | | | | Specificity | | | |
| --- | --- | --- | --- | --- | --- | --- | --- | --- | --- | --- |
| Training |  |  | CRTL | HG | EMPA | EMPA+HG | CRTL | HG | EMPA | EMPA+HG |
| Lipidomics | 4 | 100,00 | 100,00 | 100,00 | 100,00 | 100,00 | 100,00 | 100,00 | 100,00 | 100,00 |
| Metabolomics | 2 | 87,50 | 100,00 | 100,00 | 75,00 | 75,00 | 91,67 | 100,00 | 91,67 | 100,00 |
| Proteomics | 4 | 100,00 | 100,00 | 100,00 | 100,00 | 100,00 | 100,00 | 100,00 | 100,00 | 100,00 |

**Table S5:** Values of PLS-DA performances. It reports the values of accuracy, sensitivity and specificity for the cross-validation (leave one replicate out). Model built with all the modality independently.

| Model | nLVs | CC (%) | Sensitivity | | | | Specificity | | | |
| --- | --- | --- | --- | --- | --- | --- | --- | --- | --- | --- |
| Cross-validation |  |  | CRTL | HG | EMPA | EMPA+HG | CRTL | HG | EMPA | EMPA+HG |
| Lipidomics | 4 | 68,75 | 75,00 | 25,00 | 100,00 | 75,00 | 100,00 | 91,67 | 91,67 | 75,00 |
| Metabolomics | 2 | 75,00 | 50,00 | 100,00 | 75,00 | 75,00 | 83,00 | 100,00 | 83,33 | 100,00 |
| Proteomics | 45 | 100,00 | 100,00 | 100,00 | 100,00 | 100,00 | 100,00 | 100,00 | 100,00 | 100,00 |

**Table S6:** Values of PLS-DA performances. It reports the values of accuracy, sensitivity and specificity for the test set. Model built with all the modality independently. Two repetitions were used as an independent test set.

| Model | CC (%) | Sensitivity | | | | Specificity | | | |
| --- | --- | --- | --- | --- | --- | --- | --- | --- | --- |
| Test |  | CRTL | HG | EMPA | EMPA+HG | CRTL | HG | EMPA | EMPA+HG |
|  |  |  |  |  |  |  |  |  |  |
| Lipidomics | 100,00 | 100,00 | 100,00 | 100,00 | 100,00 | 100,00 | 100,00 | 100,00 | 100,00 |
| Metabolomics | 62,50 | 50,00 | 100,00 | 100,00 | 0 | 100,00 | 100,00 | 50,00 | 100,00 |
| Proteomics | 75,00 | 50,00 | 100,00 | 50,00 | 100,00 | 100,00 | 100,00 | 100,00 | 66,67 |
